# Navigator-3, a modulator of cell migration, may act as a suppressor of breast cancer progression

**DOI:** 10.15252/emmm.201404134

**Published:** 2015-02-12

**Authors:** Hadas Cohen-Dvashi, Nir Ben-Chetrit, Roslin Russell, Silvia Carvalho, Mattia Lauriola, Sophia Nisani, Maicol Mancini, Nishanth Nataraj, Merav Kedmi, Lee Roth, Wolfgang Köstler, Amit Zeisel, Assif Yitzhaky, Jacques Zylberg, Gabi Tarcic, Raya Eilam, Yoav Wigelman, Rainer Will, Sara Lavi, Ziv Porat, Stefan Wiemann, Sara Ricardo, Fernando Schmitt, Carlos Caldas, Yosef Yarden

**Affiliations:** 1 Department of Biological Regulation, Weizmann Institute of ScienceRehovot, Israel; 2 Cancer Research UK Cambridge Research Institute, Li Ka Shing CentreCambridge, UK; 3 Physics of Complex Systems, Weizmann Institute of ScienceRehovot, Israel; 4 Chemical Physics, Weizmann Institute of ScienceRehovot, Israel; 5 Division of Molecular Genome Analysis, German Cancer Research Centre (DKFZ)Heidelberg, Germany; 6 Biological Services, Weizmann Institute of ScienceRehovot, Israel; 7 IPATIMUP - Institute of Molecular Pathology and Immunology, Medical Faculty of the University of PortoPorto, Portugal

**Keywords:** cancer, cell migration, cytoskeleton, growth factor, microtubules

## Abstract

Dissemination of primary tumor cells depends on migratory and invasive attributes. Here, we identify *Navigator-3* (*NAV3*), a gene frequently mutated or deleted in human tumors, as a regulator of epithelial migration and invasion. Following induction by growth factors, NAV3 localizes to the plus ends of microtubules and enhances their polarized growth. Accordingly, *NAV3* depletion trimmed microtubule growth, prolonged growth factor signaling, prevented apoptosis and enhanced random cell migration. Mathematical modeling suggested that NAV3-depleted cells acquire an advantage in terms of the way they explore their environment. In animal models, silencing *NAV3* increased metastasis, whereas ectopic expression of the wild-type form, unlike expression of two, relatively unstable oncogenic mutants from human tumors, inhibited metastasis. Congruently, analyses of > 2,500 breast and lung cancer patients associated low *NAV3* with shorter survival. We propose that NAV3 inhibits breast cancer progression by regulating microtubule dynamics, biasing directionally persistent rather than random migration, and inhibiting locomotion of initiated cells.

## Introduction

In adult organisms, cell migration is essential for wound healing and for immune functions, whereas aberrant migration contributes to metastasis of cancer cells (Yamaguchi *et al*, 2005; Friedl *et al*, 2012). Cell migration might assume a random pattern, which allows cells to explore their local environment, or a directional (persistent) mode that rapidly transfers cells to new, distant environments. Directionality acquisition involves regulation by microtubules (Pegtel *et al*, 2007), integrins and Rho family GTPases like Rac1 (Nobes & Hall, 1999; Etienne-Manneville & Hall, 2003). For example, the Rac activator Tiam1 forms a complex with Par proteins. This association inhibits Tiam1 and thereby helps establish front–rear polarity and persistent migration (Pegtel *et al*, 2007). Conversely, Slit-Robo GAP 1 (srGAP1) possesses a GAP activity specific to Rac1 and modulates within lamellipodia both Rac1 and RhoA, which are mutually inhibitory (Yamazaki *et al*, 2013). Consistent with this, tuning of Rac1 activity might promote multiple lamellipodial protrusions, thereby enhancing the random type of migration (Pankov *et al*, 2005).

Multiple processes involved in cell motility are regulated by growth factors and their receptors, such as EGFR (Wells *et al*, 2006). These processes include transient activation of proteases, kinases and small GTPases (Ridley, 2011). In addition, EGF-induced transcriptional alterations instigate critical switches, such as loss of the epithelial cadherin (Nieman *et al*, 1999) and gain of Cten (Katz *et al*, 2007). The spatial organization of active EGFRs and resultant, locally restricted intracellular messengers appear to stabilize lamellipodia and thereby maintain the direction of movement (Maheshwari *et al*, 2001; Harms *et al*, 2005; Petrie *et al*, 2009). Congruently, it has been shown that inhibiting the polarized vesicular trafficking of EGFRs to the leading edge specifically reduced directionally persistent migration (Chibalina *et al*, 2010).

The present study was motivated by reports that associated EGFR ligands in metastasis (Minn *et al*, 2005). We employed mammary epithelial cells, for which EGF is a motogenic cue (Amit *et al*, 2007; Tarcic *et al*, 2012), and identified neuron navigator 3 (NAV3), a microtubule-binding protein aberrantly expressed in tumors (Karenko *et al*, 2005; Wood *et al*, 2007; Bleeker *et al*, 2009; Maliniemi *et al*, 2011; Carlsson *et al*, 2012, 2013), as a regulator of cell migration and invasion. NAV3 undergoes transcriptional induction by EGF, localizes to the tips of microtubules and enhances their growth. By silencing NAV3 expression and studying mutant forms in animal models, we concluded that NAV3 inhibits metastasis. This conclusion is consistent with the ability of relatively low NAV3 abundance to predict poorer prognosis of cancer patients. Conceivably, by regulating microtubule dynamics, NAV3 inhibits the random mode of cell migration and restrains dissemination of tumor cells.

## Results

### EGF-induced migration of mammary cells involves up-regulation of *NAV3*

Our previous study identified EGR1 and the upstream mitogen-activated protein kinase pathway (MAPK, specifically ERK) as drivers of EGF-induced migration of cultured MCF10A human mammary cells (Tarcic *et al*, 2012). Accordingly, inhibitors of either EGFR or MEK strongly impaired migration (Supplementary Fig S1A). Quantification of migration directionality relates the linear distance between the start and end points (D) to the total distance (T) traveled. The rose plots of individual tracks (Supplementary Fig S1B and 1C) indicated that EGF enhanced directional persistence (D/T) and also accelerated the rate of migration, in line with the well-characterized chemotactic action of this growth factor (Blay & Brown, 1985). Interestingly, inhibiting MEK increased directional persistence (Supplementary Fig S1C), whereas Rac1 inhibition (using NSC23766; data not shown) decelerated migration and increased persistence, in line with the reported ability of Rac1 to augment random migration (Pankov *et al*, 2005). Consistent with a requirement for newly synthesized macromolecules, when we inhibited transcription or translation, both velocity and persistence decreased (Supplementary Fig S1D and 1E).

**Figure 1:**
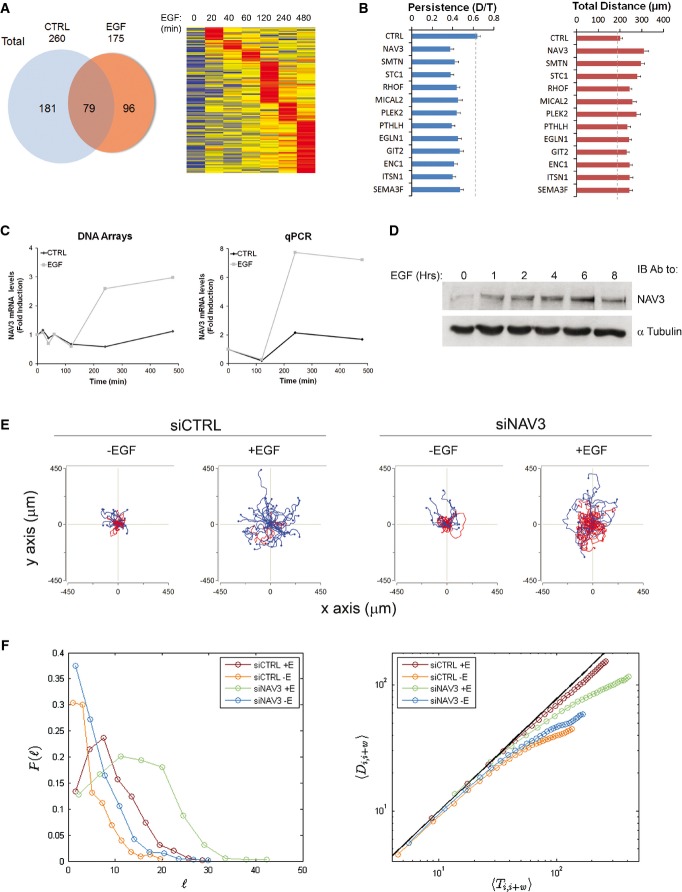
Inducible NAV3 decreases velocity and enhances persistence of mammary cell migration
The results of oligonucleotide array screens of EGF- or control-stimulated MCF10A cells (Amit *et al*, 2007) were re-analyzed, and genes induced by > twofold were included in the Venn diagram. The heatmap presents a time series of genes induced upon EGF treatment.siRNAs specific to the indicated genes were used to treat MCF10A cells, and migration tracks were subsequently quantified (see Supplementary Fig S1A). Shown are means ± SEM of three experiments; 60 cells were analyzed in each case. The results of statistical analyses (two-tailed Student's *t*-test) are shown in Supplementary Table S1.Changes in NAV3 expression levels in response to EGF were extracted from DNA arrays or determined by means of quantitative PCR.Serum-starved MCF10A cells were treated with EGF (10 ng/ml) for the indicated time intervals, and cell extracts were subjected to immunoblotting (IB), as indicated.Shown are trajectories of MCF10A cells expressing either siCTRL or siNAV3 and migrated on collagen in the absence or presence of EGF. Red tracks indicate low persistence (< 0.3). See quantification of the data in Supplementary Fig S2D.Quantitative analysis of the mode of migration of NAV3-depleted cells. The distribution of step sizes was computed, normalized and presented as probability distribution functions (left panel). Log-log plots indicating modes of migration for each of the four conditions are shown (right panel). Note that only EGF-treated siCTRL cells exhibit a persistent walk (diagonal), whereas the other three asymptote, as expected for random walks.
Source data are available online for this figure.

To identify newly synthesized molecules that regulate migration, we considered our previously reported set of EGF-induced genes (Amit *et al*, 2007). This set of genes emerged when we contrasted serum-activated cells (which undergo proliferation) and migrating, EGF-stimulated cells. Re-analysis of the data listed 96 EGF-specific genes (Supplementary Table S1 and Fig1A). Based on specificity to an EGF signal and motility annotation, we selected 12 genes from the list and individually silenced them (Fig1B; see part 3 of Supplementary Table S1). Two genes displayed strong effects on directional persistence: *PTHLH*, a well-known regulator of metastasis (Sloan & Anderson, 2002), and neuron navigator 3 (*NAV3*), the orthologue of *unc-53*, a gene involved in axon guidance in worms (Maes *et al*, 2002). Importantly, NAV3 binds microtubules (MTs) and its action is essential for axon guidance also in mammals (Martinez-Lopez *et al*, 2005; Stringham & Schmidt, 2009). Moreover, previous studies identified *NAV3* as a putative tumor suppressor in cutaneous T-cell lymphoma and in the associated lung tumors (Karenko *et al*, 2005; Hahtola *et al*, 2008). Although a later study was unable to confirm these observations (Marty *et al*, 2008), multiple point mutations were found within *NAV3* in colorectal, pancreatic and melanoma tumors (Wood *et al*, 2007; Bleeker *et al*, 2009). Notably, several EGFR ligands were able to up-regulate NAV3 (Supplementary Fig S2A and 2B), and induction was detectable at both mRNA and protein levels (Fig1C and D). In conclusion, stimulation of EGFR accelerated the speed of mammary cell migration and enhanced directional persistence. This response was associated with induction of a specific group of genes, including *NAV3*, a putative tumor suppressor whose mechanism of action is poorly understood.

### Migration assays and mathematical modeling imply that NAV3-depleted cells acquire a random mode of migration and higher probability of hitting rare targets

**Figure 2:**
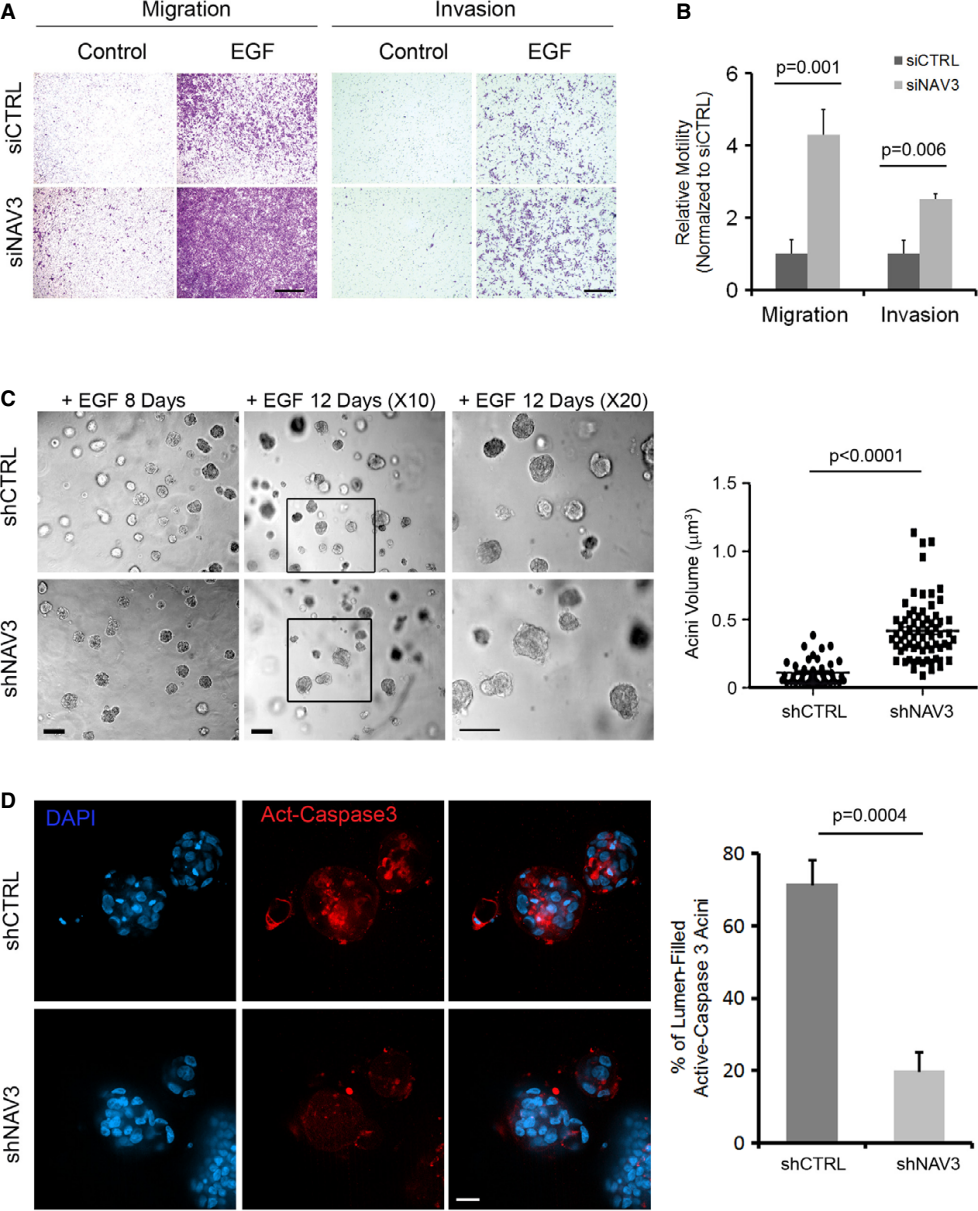
Depletion of NAV3 accelerates migration and invasion and alters 3D morphology of mammary cells
MCF10A cells, pre-transfected with the indicated siRNAs, were assayed for migration or invasion in the absence or presence of EGF. Cells that reached the filter's bottom were stained and photographed. Scale bars, 150 μm (migration) and 100 μm (invasion).The results from (A) are depicted as means ± SD (four experiments).Phase microscopy analysis of shCTRL and shNAV3 MCF10A cells (scale bars, 50 μm), which were plated in Matrigel and grown for 8 or 12 days in the presence of EGF (20 ng/ml). Acini were photographed and quantified for their volumes using ImageJ. The right panel shows analysis of acinus size after 8 days in culture (data represent three different experiments, and *P*-value was calculated using *t*-test).shCTRL or shNAV3 acini (day 10) were immunostained for activated caspase-3 (red) and DAPI (scale bar, 20 μm). Clusters positive for activated caspase-3 were counted and quantified in three experiments (means ± SD).

To study NAV3, we applied specific siRNA oligonucleotides and observed approximately 80% reduction in mRNA levels when using single oligonucleotides or their quadruplet mixture (Supplementary Fig S2C), which we adopted in further analyses. Unlike control MCF10A cells, which displayed EGF-induced enhancement of speed and persistence, NAV3-silenced cells exhibited relatively rapid migration, and they completely lost the ability to enhance persistence in response to EGF (Fig1E and 2 Supplementary Fig S2D). Conversely, ectopic expression of NAV3 enhanced directional persistence and slowed down the rate of cell migration (Supplementary Fig S2E). These observations prompted us to computationally model the effect of NAV3 on cell migration and relate NAV3 depletion to tumor progression. As input, we used experimental data from siRNA- and EGF-treated cells (see Supplementary Movie S1). To reduce complexity, we assumed a simplified 2D model, which ignored tissue barriers. Data for individual cells were provided in terms of position {*x*_*i*_, *y*_*i*_} for times i = 1, 2, 3,··· 31 (10 min intervals). This allowed us to compute the total length of each walk, 

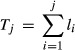
(*l*_i_ denotes the length of the *i*^th^ step), and the end-to-end distance, *D*_1,*j*_. Next, we computed the distribution of all normalized step sizes and plotted the respective probability distribution function (pdf; Fig1F, left). Likewise, by plotting log *D*_1,*j*_ as a function of log *T*_*j*_, we resolved the character of the respective trajectories (Fig1F, right). These analyses indicated that only EGF-treated siCTRL cells displayed a persistent walk; all other treatments yielded random walk patterns. To exemplify potential relevance to tumor progression, one might consider the best scenario to hit a rare target: In case the persistent walk goes in the right direction, from {0,0} to {*x*,*y*}, it would get there in the order of 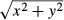 steps. Although a random walker would take much longer, of the order of 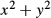 steps, according to the model, it would eventually hit the target with probability 1. In other words, the quantitative modeling we performed implied that loss of NAV3 would bias the random mode of cell migration. Because random walkers better explore their environment, the model predicted increased probability of NAV3-depleted cells to hit rare targets, such as transient openings within the basement membrane surrounding primary tumors.

### Loss of NAV3 induces invasiveness and alters patterns of 3D growth

Depletion of NAV3 using siRNA oligonucleotides increased not only migration rates but also the ability of mammary cells to invade through a layer of extracellular matrix (Fig2A and B). To assess relevance to tumorigenesis, we used 3D models of mammary cells. MCF10A cells stably expressing shNAV3 were incubated in a three-dimensional (3D) tissue-like matrix. Unlike the small acini formed by control cells, shNAV3 cells developed larger 3D structures (Fig2C), which displayed no lumen. Because lumen formation within acini requires apoptosis of centrally located cells (Debnath *et al*, 2002; Reginato *et al*, 2005), we examined the prediction that loss of NAV3 expression would inhibit apoptosis. Two lines of evidence supported this possibility: Staining of acini with an antibody specific to the cleaved form of caspase-3, a marker of apoptosis, detected reduced levels of the cleaved enzyme within the core of NAV3-depleted acini (Fig2D). Likewise, induction of apoptosis of MCF10A cells by using cisplatin, a cytotoxic agent, increased cleavage of caspase-3 in control cells, but cells stably expressing shNAV3 displayed only the intact form of the protease (Supplementary Fig S3A). Interestingly, bromo-deoxyuridine incorporation assays disfavored higher proliferation of shNAV3 cells (Supplementary Fig S3B). In conclusion, loss of NAV3 increases invasiveness and enlarges acini, probably by inhibiting apoptosis rather than by accelerating cell proliferation.

### *NAV3* silencing enhances invadopodia formation *in vitro* and metastasis *in vivo*

**Figure 3:**
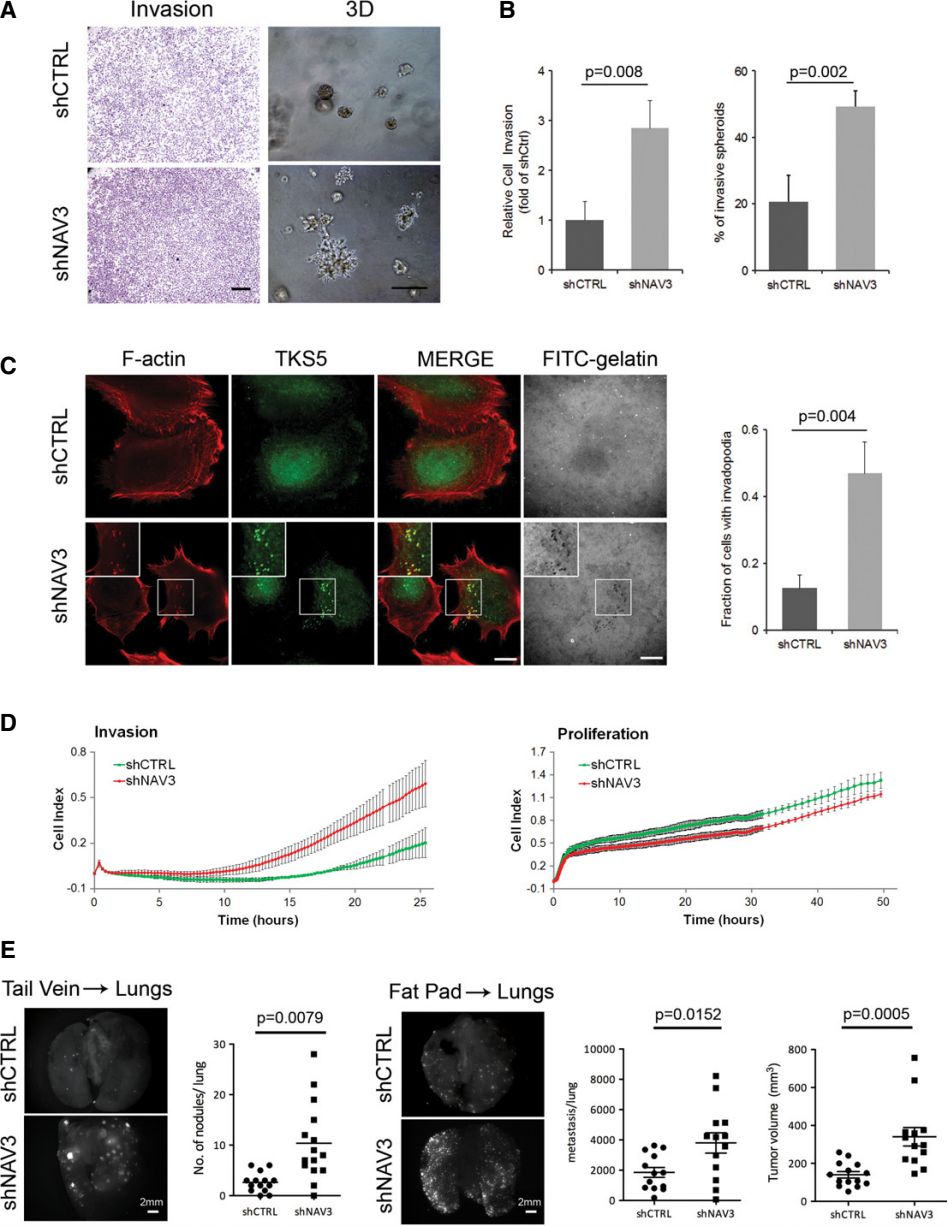
NAV3 knockdown enhances invasiveness of mammary cancer cells *in vitro* and their metastasis *in vivo*
shCTRL- and shNAV3-MDA-MB-231 cells were plated in invasion chambers, and 18 h later, invaded cells were stained. For 3D growth, cells were seeded in Matrigel and photos were taken 5 days later (scale bars, 200 μm and 50 μm).Histograms (means ± SD) of the results presented in (A) (from three experiments).MDA-MB-231 derivatives were plated on coverslips pre-coated with a fluorescent gelatin. Two hours later, cells were probed for actin and TKS5. The number of cells with invadopodia were quantified in three experiments. Shown are means ± SD. Each determination included 80 cells. Scale bar, 10 μm.MDA-MB-231 derivatives were monitored for invasion and proliferation using real-time impedance (xCELLigence).The indicated RFP-expressing MDA-MB-231 derivatives were injected either intravenously (1.5 × 10^5^ per mouse; tail vein), or into the mammary fat pad (2.5 × 10^6^ cells per mouse) of SCID mice. Four weeks later, lungs from the tail vein-injected mice were photographed and examined for RFP signals corresponding to relatively large nodules, which were quantified. Photographs and numbers of the relatively small metastases, as well as tumor size from mice injected into the fat pad, were obtained 8 weeks post-implantation (right panels).

To ascertain involvement of NAV3 in invasiveness, we selected a subline of the highly invasive mammary cancer MDA-MB-231 cells, which overexpresses the red fluorescent protein (RFP). NAV3-depleted derivatives of these cells displayed higher invasion in two assays: penetration through Matrigel-coated filters and development of invasive structures when grown in a 3D matrix (Fig3A and B). To examine matrix proteolysis, we plated cells on fluorescent gelatin and probed for the actin- and TKS5-enriched, matrix-degrading organelles called invadopodia (Murphy & Courtneidge, 2011). In this assay, shNAV3 cells displayed approximately threefold more proteolytic protrusions (Fig3C). These effects were likely due to strongly enhanced invasion and weakly inhibited proliferation, as reflected by impedance assays (Fig3D).

Next, we injected shCTRL and shNAV3 cells into the tail vein of mice and quantified lung metastases. NAV3 knockdown increased by approximately fourfold the numbers of fluorescent nodules in lungs (Fig3E, left panels). Using a second model, we implanted cells in the fat pad and 6 weeks later monitored both tumor size and lung metastases (Fig3E, right panels). Interestingly, despite relatively weak *in vitro* effects on cell proliferation, NAV3-depleted cells gave rise to larger tumors, which disseminated significantly more metastases to the lungs. Taken together, two animal models (along with a third, gain-of-function model; see Fig 6D and E) indicated that lung metastasis of mammary tumor cells might be accelerated when *NAV3* is depleted in tumors.

### NAV3 stabilizes growing microtubules

MTs control persistent migration by establishing front–rear polarity (Pegtel *et al*, 2007), which might relate to their ability to dictate the sites of both substrate adhesion (Ezratty *et al*, 2005) and lamellipodia formation (Waterman-Storer *et al*, 1999). The rapid switches of MTs between growth and shrinkage are controlled by +TIP proteins, such as the end binding (EB) family, including EB1 (Kaverina & Straube, 2011). Because the navigators are +TIPs (van Haren *et al*, 2009), we assumed that NAV3 controls migration by binding to the growing ends of MTs. Several lines of evidence supported this scenario: Ectopic expression of a peptide-tagged NAV3 showed localization of the tips of MTs (Supplementary Fig S4), and live cell imaging confirmed co-localization of GFP-NAV3 and RFP-EB1 to the end of MTs (Supplementary Fig S5). Likewise, co-expression of GFP-NAV3 and mCherry-α-tubulin demonstrated dynamic tracking of the tips of growing MTs by NAV3 (Supplementary Fig S6A and Supplementary Movie S2). In addition, by applying MT co-sedimentation assays, we validated physical interactions between NAV3 and MTs (Supplementary Fig S6B). Next, we tracked individual plus ends and found that ectopic NAV3 decreased catastrophe rates, as well as accelerated MT rescue rates (Supplementary Fig S6C). Correspondingly, the MTs of cells ectopically expressing NAV3 spent significantly longer time growing than shrinking (Supplementary Fig S6C, lower panel). The involvement of EB1 was confirmed by detecting co-localization of NAV3 and a fluorescent derivative of EB1 at plus tips, an observation we supported by using a pull-down assay (Supplementary Fig S6D).

**Figure 4:**
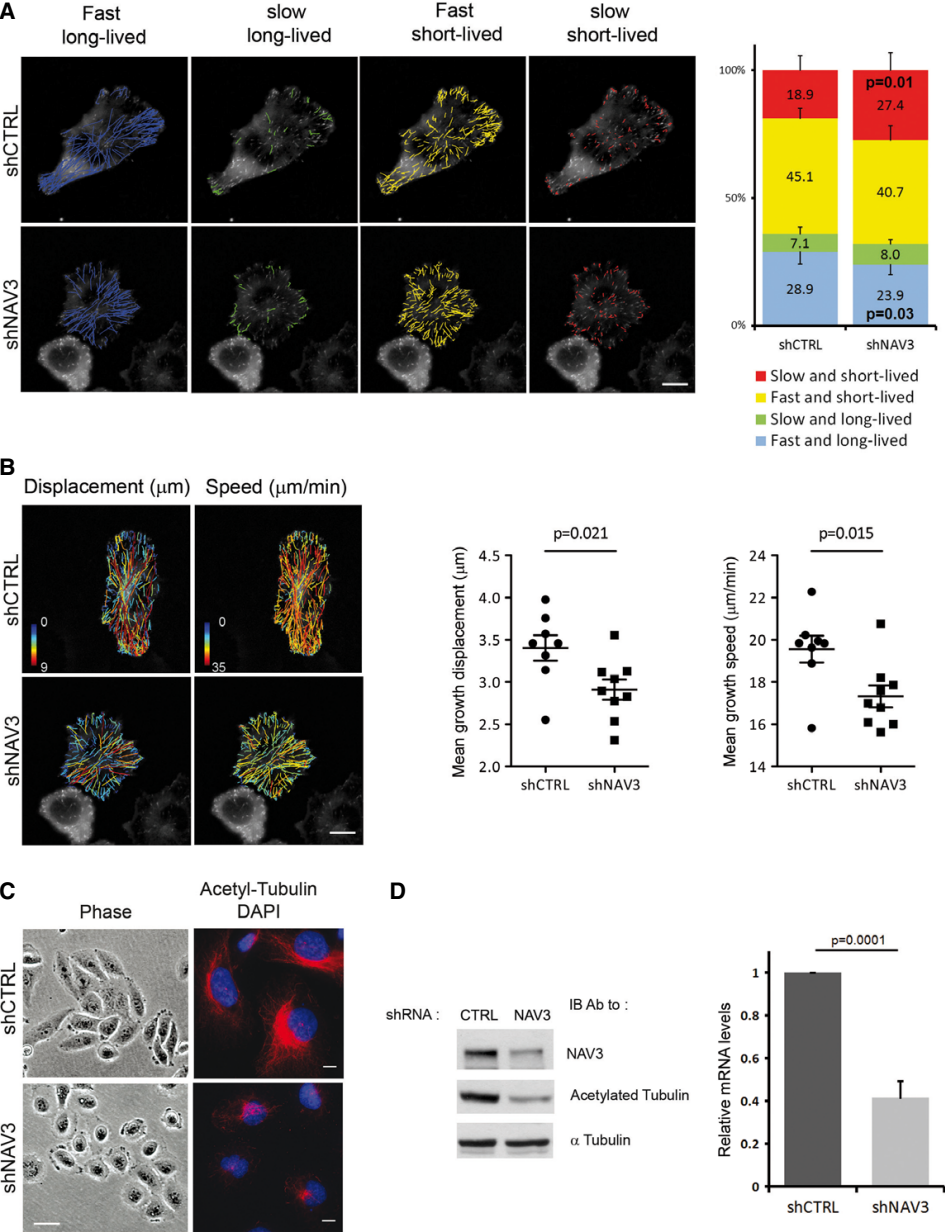
NAV3 regulates microtubule dynamics and stability
The indicated MDA-MB-231 derivatives were transfected with a GFP-EB1 plasmid. Time-lapse images were taken at 2-s intervals, and MT ends were tracked using plusTipTracker. Overlays of four MT subpopulations and representative cells are shown (scale bar, 10 μm), along with the relative proportions of subpopulations (right panel). Data (mean ± SD) were pooled from three experiments. *P*-values are shown for two groups.Cells from (A) were used to derive representative overlays of MT dynamics maps, depicting displacement and speed (scale bar, 10 μm). Scatter plots of mean displacement and speed of MT tracks are shown. Nine representative cells (three of each experiment) were selected for analysis of MT's mean displacement and speed.The indicated derivatives of MDA-MB-231 cells were photographed, either directly or following immunofluorescence analysis using the indicated antibodies. Nuclei were stained with DAPI (scale bars, 20 μm and 10 μm, left and right columns, respectively).The indicated stable derivatives of MDA-MB-231 cells were analyzed using RT–PCR for the levels of NAV3 expression (right panel). Shown are means ± SD from three independent analyses. Whole-cell extracts were prepared and subjected to immunoblotting with anti-NAV3, anti-acetylated tubulin and anti-α-tubulin antibodies (left panel).

Taken together, our results proposed that NAV3 stabilizes MTs. To corroborate this, we used shRNAs and MDA-MB-231 cells. In assays which are not presented (see also Fig4D), we found that shRNA-expressing cells lost approximately 60% of their endogenous NAV3 molecules. Hence, these cells were transfected with a GFP-EB1 plasmid, and trajectories were recorded by utilizing plusTipTracker, a software that permits tracking of +TIPs (Applegate *et al*, 2011) (Fig4A). Along with effects on morphology, we confirmed altered MT dynamics in shNAV3 cells: an increased presence of the slow and short-lived sub-population of MTs and diminution of the fast and long-lived class. In addition, we analyzed sub-tracks of MTs for their speed and displacement and found that these were reduced in NAV3-depleted cells (Fig4B). In conclusion, several independent lines of evidence indicated that NAV3 shifts MT dynamics from shrinking and pausing toward sustained growth.

### NAV3 silencing associates with reduced acetylation of tubulin, altered EGFR trafficking and disoriented chemotaxis

Long-lived MTs accumulate covalent modifications, such as acetylation (Hammond *et al*, 2008). Using nocodazole, an inhibitor of MT polymerization, we observed an almost complete disappearance of acetylated MTs in COS-7 cells, but acetylated MTs were still detectable in nocodazole-treated cells overexpressing NAV3 (Supplementary Fig S6E). Depletion of NAV3 using shRNA further supported the notion that this protein stabilizes MTs: Markedly reduced levels of acetylated tubulin were observed using immunofluorescence and immunoblotting of NAV3-depleted MDA-MB-231 cells (Fig4C and D; note that the elongated morphology of control cells was replaced by roundish and dispersed patterns of shNAV3 cells).

**Figure 5:**
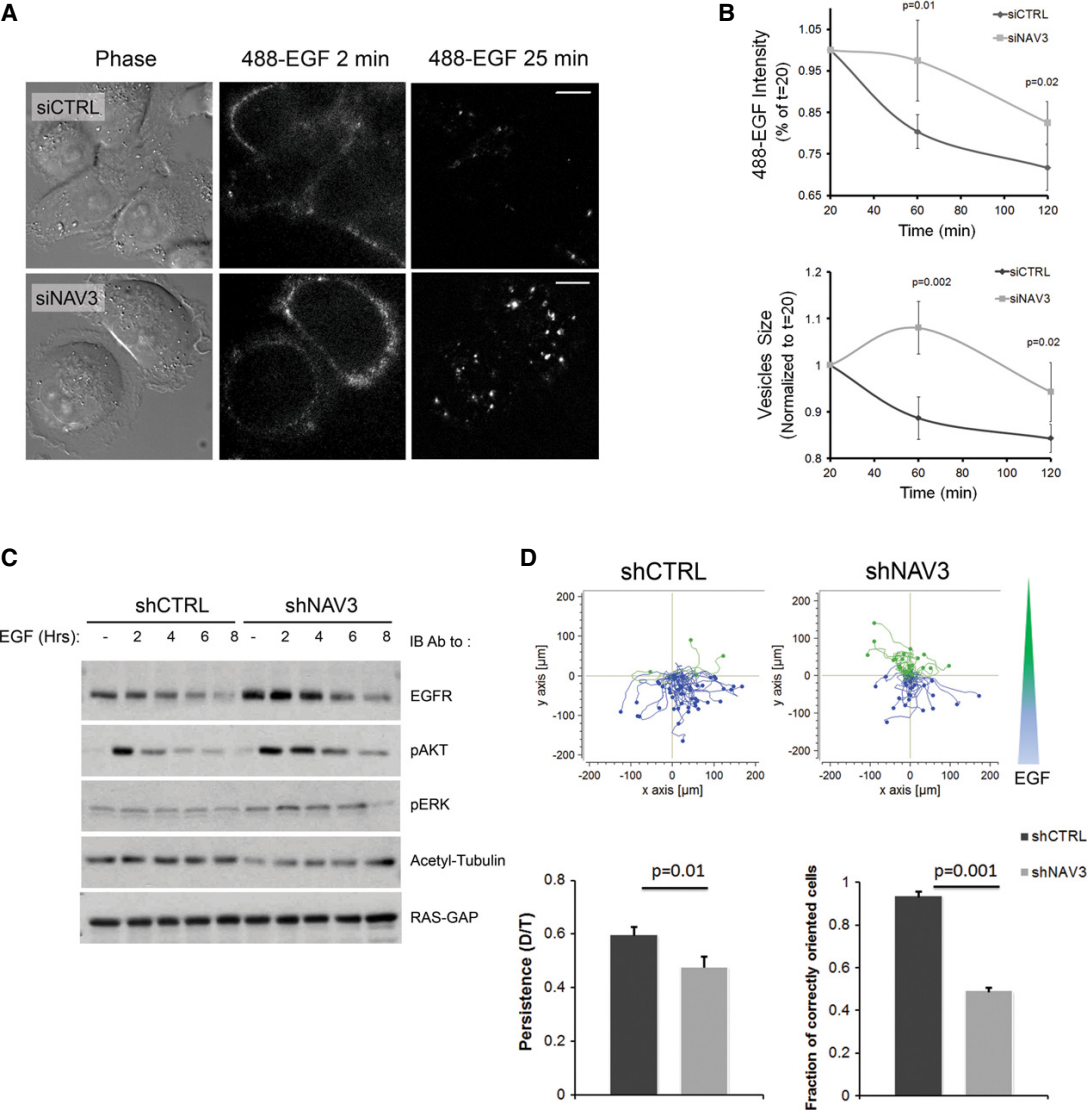
NAV3 knockdown associates with altered EGFR trafficking and disoriented chemotaxis
MDA-MB-231 cells, pre-treated with the indicated siRNAs, were stimulated with Alexa Fluor 488-EGF (20 ng/ml) and imaged for 25 min. Frames taken at 2 and 25 min are shown (scale bars, 10 μm; see Supplementary Movie S3).Cells were stimulated with EGF (20 ng/ml; 10 min) as in (A), acid-washed and incubated at 37°C for the indicated intervals. Thereafter, cells were detached, fixed and analyzed using imaging flow cytometry (ImageStreamX). Shown are means ± SD from three experiments.MDA-MB-231 derivatives were serum-starved and stimulated with EGF for the indicated time intervals. Whole extracts were subjected to immunoblotting as indicated.Rose plots obtained with MDA-MB-231 derivatives exposed to an EGF gradient (shown as a blue and green wedge) in chemotaxis chambers. Blue lines indicate properly oriented tracks. Histograms (mean ± SEM) show quantification of persistence and chemotactic orientation from three experiments (45 cells).

MT acetylation increases the affinity of motor proteins like kinesin-1 to their tracks (Hammond *et al*, 2008), and de-acetylation inhibits EGFR endocytosis (Deribe *et al*, 2009; Gao *et al*, 2010). Hence, we examined endocytosis of a fluorescent derivative of EGF (Fig5A; Supplementary Movie S3). In control cells, EGF initially associated with the cell surface, internalized and later underwent clearance by degradation and recycling. By contrast, in NAV3-depleted cells, we observed higher surface labeling, which was later replaced by relatively long-lived intracellular puncta of EGF. Cell sorter-based assays confirmed slower decay of the cell-associated EGF signal of NAV3-silenced cells, along with enlarged EGF-containing vesicles (Fig5B). These observations suggested delayed removal of EGFR from the cell surface, along with a defect in post-internalization processing of ligand–receptor complexes. Along this vein, immunoblotting of NAV3-depleted cells detected increased EGFR abundance, as well as prolonged downstream signaling to ERK and AKT (Fig5C). Because intact trafficking is critical for both EGFR localization to the leading edge and for the chemotactic response to EGF gradients (van Rheenen *et al*, 2007), we predicted that NAV3-depleted cells would aberrantly respond to a chemotactic cue. Indeed, most control cells migrated upstream a concentration gradient of EGF, but NAV3-depleted cells failed reading the gradient (Fig5D). In conclusion, by stabilizing MTs, NAV3 might enable normal trafficking of EGFR and timely termination of intracellular signals, as well as support properly oriented chemotaxis, which is consistent with a role for NAV3 in directionally persistent cell migration.

### Unlike wild-type *NAV3,* point-mutated cancer alleles are relatively unstable variants devoid of persistency induction and metastasis suppression

**Figure 6:**
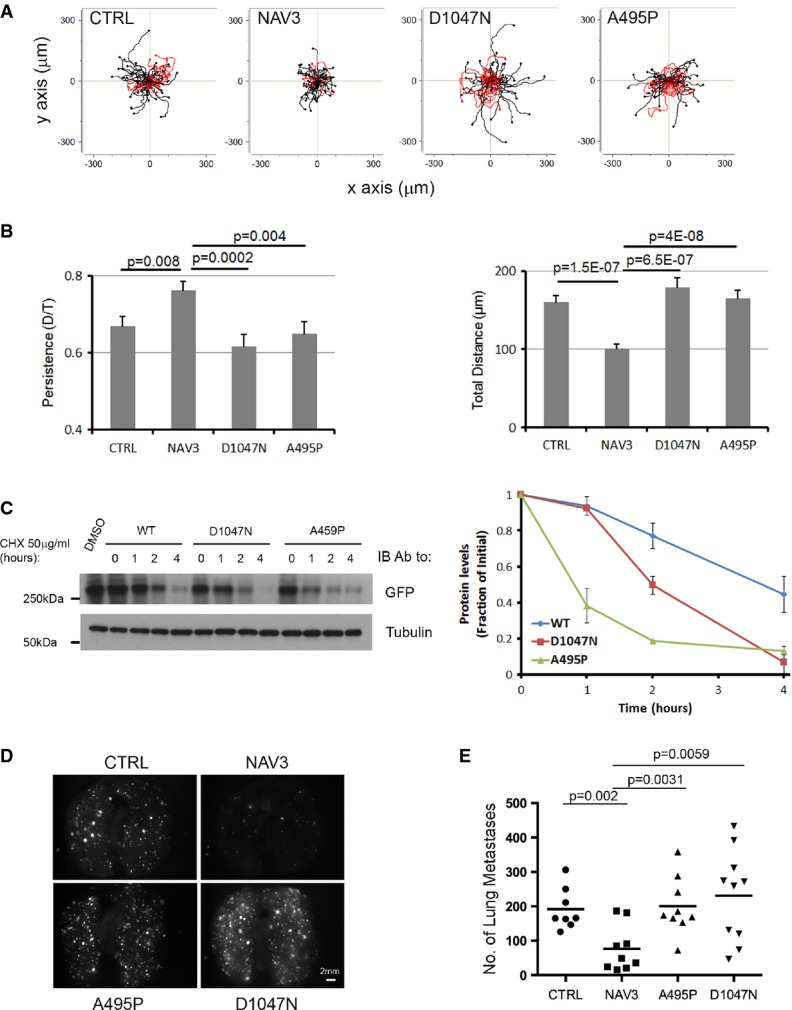
Unlike wild-type *NAV3,* mutant cancer alleles are devoid of persistency induction and metastasis suppression
A MCF10A cells were plated on collagen, and 24 h later, they were treated with EGF (10 ng/ml). Shown are trajectories of cells transiently expressing the indicated forms of NAV3. Red tracks indicate relatively low persistence (< 0.6).B Quantification of migration parameters (from A) is shown as mean ± SEM (60 cells and three experiments).C HEK293 cells previously transfected with EGFP-NAV3 (WT), EGFP-NAV3-D1047N or EGFP-NAV3-A459P were treated with cycloheximide (CHX; 50 μg/ml) or with the solvent, DMSO, for the indicated time intervals, followed by cell lysis and immunoblotting using anti-EGFP and anti-α-tubulin antibodies. The signals obtained were quantified and normalized to time zero (untreated cells; right panel).D, E The indicated mCherry-expressing MDA-MB-231 derivatives were injected intravenously (1.5 × 10^5^ per mouse) using 5-week-old female SCID mice. Four weeks later, lungs were photographed and the numbers of lung metastases were determined.
Source data are available online for this figure.

Both deletion of *NAV3* (Karenko *et al*, 2005) and point mutations within several structural domains (Wood *et al*, 2007; Bleeker *et al*, 2009) (see a scheme in Supplementary Fig S7A) have been reported in various cancers. Five somatic mutations (A495P, Q607H, D1047N, G2097V and S2341I) frequently appear in the genomes of CRC specimens, thus designating *NAV3* as one of the most frequently mutated genes of this disease. Another mutation (D220H), which was found in breast cancer, received a high “passenger” probability score. A nonsense mutation (Q200*) was found only in melanomas (Bleeker *et al*, 2009), and four missense mutations were found in renal cell carcinoma (Guo *et al*, 2012). To address potential tumorigenic mechanisms, we expressed in cultured cells GFP fusions of two driver mutants (A495P and D1047N) and found that both retained physical association with EB1 and co-localized with the growing tip of MTs (Supplementary Fig S7B). Nevertheless, the mutants lost the ability to increase persistence and decelerate migration when introduced into mammary cells (Fig6A and B). Moreover, unlike the ability of wild-type NAV3 to protect MTs from nocodazole-induced de-acetylation, the mutants partly lost this attribute (Supplementary Fig S7C). In line with mutation-mediated loss of the MT-stabilizing function, tracking individual plus ends revealed that the mutants were less active than wild-type NAV3 in controlling catastrophe and rescue (Supplementary Fig S7D). These observations, along with the wide distribution of mutations along the NAV3 protein, raised the possibility that some mutants are unstable. To determine relative rates of decay, we blocked new protein synthesis, using cycloheximide, and followed the decay of wild-type NAV3 in comparison with the A495P and D1047N mutants (Fig6C). The results clearly indicated that the two mutants acquired significantly shorter rates of decay, as compared to the relatively stable, wild-type form of NAV3. This observation raised the possibility that at least some mutations, in similarity to chromosomal deletions found in certain lymphomas (Karenko *et al*, 2005), lead to reduced abundance of NAV3.

Causal relationships between the ability of wild-type NAV3 to stabilize MTs and suppress metastasis would predict reduced ability of the mutants to inhibit metastasis. As expected, intravenous injection of mCherry-labeled MDA-MB-231 cells stably expressing wild-type NAV3 was able to suppress lung metastasis (Fig6D and E). By contrast, the extent of lung colonization by cells expressing either oncogenic mutant of NAV3 was similar to the ability of CTRL cells to establish metastases in lungs (Fig6D and E). In conclusions, the mutant forms of NAV3 we examined likely represent unstable variants, which are unable to stabilize MTs, enhance persistent migration and inhibit metastasis in animals.

### Low abundance of NAV3 correlates with shorter survival of breast cancer patients

**Figure 7:**
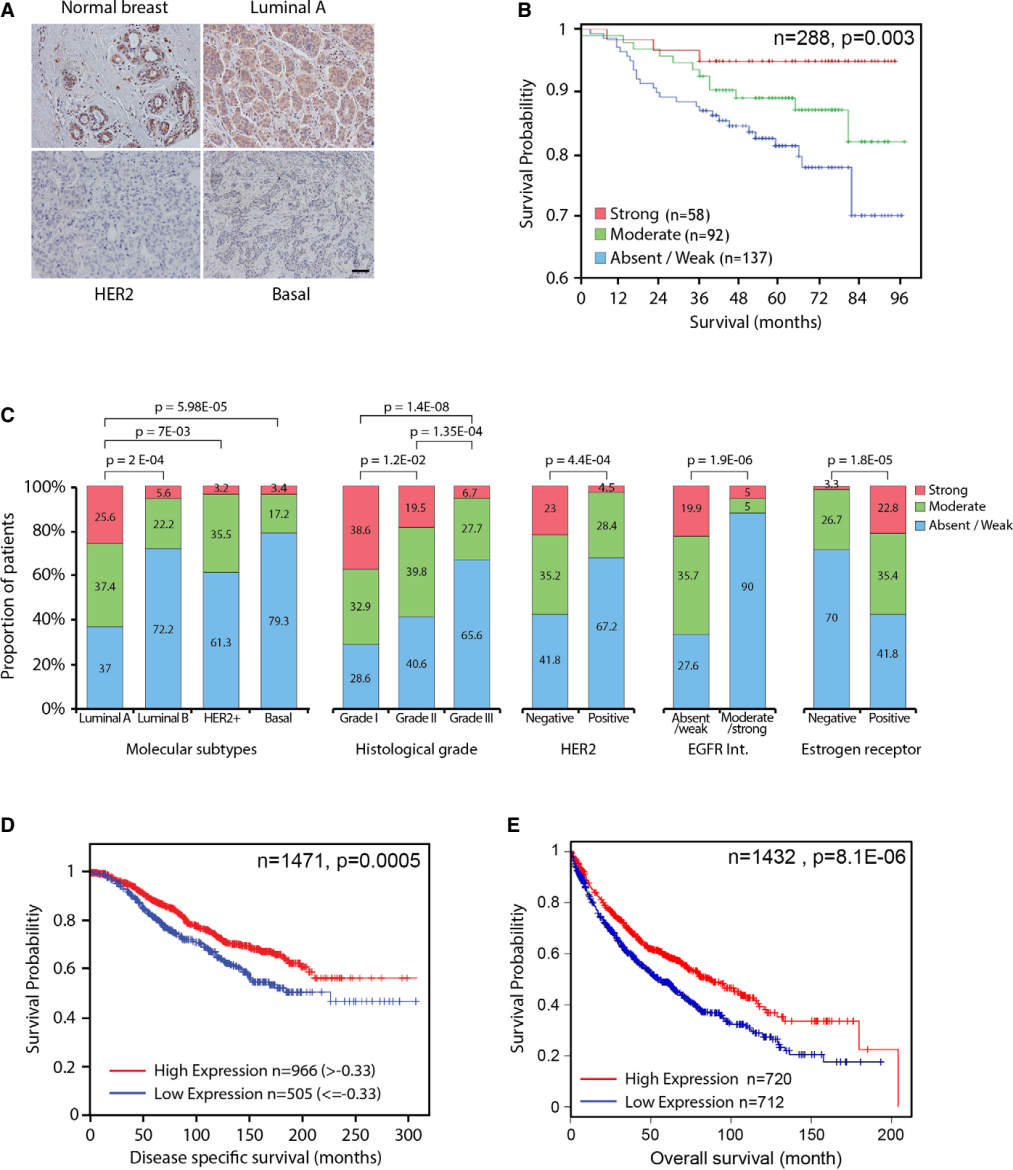
Low NAV3 abundance correlates with shorter survival of breast cancer patients
A Representative immunohistochemistry images of NAV3 in a normal breast tissue, as well as in the indicated molecular subtypes of breast cancer.B Tissue microarrays were used to stratify 288 breast cancer patients for overall survival according to NAV3 abundance.C Immunohistochemistry was used to score NAV3 expression and stratify 323 breast cancer patients according to the indicated subtypes and tumor parameters.D, E Kaplan–Meier curves depicting survival of 1,471 estrogen receptor-positive breast cancer patients (D) and 1,405 lung cancer patients (E) stratified according to NAV3 abundance.

To enable immunohistochemical analysis of NAV3, we generated a monoclonal antibody, established specificity and analyzed human breast cancer specimens. Whereas normal breast exhibited high NAV3, many tumors displayed weak or no staining (Fig7A). In accordance, overall survival in this cohort (288 patients) was longer when NAV3, as determined by means of immunohistochemistry, was highly expressed (Fig7B). In addition, inverse correlation was noted between low NAV3 and several tumor markers, as well as relatively aggressive subtypes (Fig7C and Supplementary Tables S2 and S3). Congruently, analysis of a larger breast cancer dataset (Curtis *et al*, 2012) confirmed association between low *NAV3* and shorter disease-specific survival of patients (Fig7D; 1,471 patients). This, however, was limited to estrogen receptor-positive tumors, suggesting that NAV3's aberrations arise relatively early in the tumorigenesis process, when tumors are still hormone dependent. Using multivariate Cox regression, the prognostic effect of *NAV3* was shown to be associated with histological grade (*P *=* *0.0083) and breast cancer subtype (PAM50; *P* = 0.0178). When investigating whether *NAV3* expression would associate with the recently identified 10 subgroups of breast cancer (Curtis *et al*, 2012), low *NAV3* was associated with groups with the worst outcome: iCluster5 (*P *=* *5.22e-9), iCluster9 (*P *=* *0.0007) and iCluster10 (*P *=* *1.57e-17). To extend the analysis beyond breast tumors, we examined prognostic significance in 1,405 lung cancer patients (http://kmplot.com/analysis/) and associated low NAV3 with shorter patient survival (Fig7E).

In summary, analysis of several independent cohorts of cancer patients established an association between low *NAV3* and aggressive course of disease. This association is in line with the results of our *in vitro* assays and animal studies, and together, they attribute tumor suppressor functions to *NAV3*. As we discuss below, these functions might be due to the effects of NAV3 on polarized MT dynamics, receptor endocytosis, invadopodia formation, as well as the exact mode of cellular locomotion.

## Discussion

EGF-induced migration of epithelial cells involves delayed transcription of specific mRNAs (Malliri *et al*, 1998) and microRNAs (Avraham *et al*, 2010). To identify critical genes, we concentrated on inducible mRNAs and examined migration pace and directional persistence. These focused our attention on NAV3, a multi-domain protein, previously implicated in axonal guidance (van Haren *et al*, 2009; Stringham & Schmidt, 2009). Our analyses indicate that NAV3 decorates the freshly polymerized plus ends of MTs and enhances directional persistence of cell migration. Thus, in line with a previous report (van Haren *et al*, 2009), we found that NAV3 belongs to the diverse group of +TIP proteins, which hitchhike on tip-bound EB proteins (Akhmanova & Steinmetz, 2008). Taking advantage of this location, +TIPs catalyze the addition of tubulin monomers and stabilize growing MTs (Mimori-Kiyosue *et al*, 2005). Two lines of evidence are consistent with enhancement of MT stability by NAV3: Firstly, acetylation of α-tubulin is enhanced by NAV3, and secondly, we found that NAV3 augments rescue and decreases the frequency of MT's catastrophes.

Through their roles in signaling and vesicular trafficking, MTs participate in events essential for cell migration (Etienne-Manneville, 2013). These functions rely on the asymmetric regulation of MT dynamics and stability. Importantly, NAV3-mediated stabilization of the MT network might regulate front–rear polarity of persistently migrating cells. According to previous analyses, receptor trafficking and localized signaling at the leading edge are crucial for the establishment of rear–front polarity (Jekely *et al*, 2005; Assaker *et al*, 2010; Parachoniak *et al*, 2011), and the defects in EGFR endocytosis we observed in NAV3-depleted cells are consistent with this model. In addition to the MT network, the Par polarity complex and small GTPases are also involved in the establishment of the front–rear polarity characteristic of directionally persistent migration (Pegtel *et al*, 2007). This asymmetry appears related to the ability of MTs to direct the site of actin polymerization and lamellipodia protrusion (Waterman-Storer *et al*, 1999). Once established, the front–rear axis enables extension of actin-filled protrusions in the direction of movement (Dawe *et al*, 2003). Hence, it is conceivable that NAV3-mediated stabilization of growing MTs engages polarity complexes and GTPases, thereby leading to adoption of directional persistence by EGF-stimulated cells.

Along with destabilized MTs, NAV3-silenced mammary cells exhibited abundant invadopodia. This finding is surprising in light of the absence of MTs in the early stages of invadopodia formation (Schoumacher *et al*, 2010). However, dynamic MTs are essential for matrix invasion (Nakaya *et al*, 2008), as well as for delivery of proteases. Formation and stabilization of invadopodia are relevant to the presumed tumor suppressor activity of *NAV3*. While rarely mutated in cancer, the spectrum of tumors in which aberrant NAV3 was detected is very wide: colorectal, breast, melanoma, adrenal carcinoma, as well as glioblastoma and neuroblastoma (Coy *et al*, 2002; Wood *et al*, 2007; Bleeker *et al*, 2009). Moreover, our analyses of > 2,000 cancer patients revealed strong associations between low NAV3 and shorter patient survival. In an attempt to shed light on the underlying processes, we established several animal models, which indicated that loss of NAV3 results in increased metastasis, whereas overexpression curtails metastasis.

How exactly NAV3 aberrations support metastasis and tumor progression in patients and in animal models remains unclear. Previous comparative analyses of random and directionally persistent migration proposed that high activity of Rac1 favors random migration by promoting peripheral lamellae (Pankov *et al*, 2005). EGFR activation not only elevates NAV3 expression, probably to support chemotaxis, but also stimulates phosphoinositol 3-kinase (PI3K), which activates Rac1 at the leading edge (Dise *et al*, 2008). Hence, in tumors displaying both constitutive activation of Rac1 (due to mutations in the PI3K or MEK pathways) and mutant/reduced *NAV3*, migration would be accelerated and the random mode would be favored. Along this vein, several previous studies implicated the random mode of cell migration in tumor progression. For example, oncogenic mutants of p53 promote both invasion and loss of migration directionality (Muller *et al*, 2010), and APC, a +Tip frequently mutated in cancer, is essential for directed migration of astrocytes (Etienne-Manneville & Hall, 2003). In line with these reports, our studies identified NAV3 as a promoter of persistent migration and a suppressor of breast cancer progression. Nevertheless, this study opens several new questions that relate to potential NAV3's roles in apico-basal and front–rear polarizations, as well as roles in locomotion within 3D spaces densely populated by tissue barriers.

## Materials and Methods

### Reagents and antibodies

Chemotaxis and cell tracking slides were from Ibidi (Munich, Germany), and glass-bottom dishes were from MaTek (Ashland, MA). A murine monoclonal antibody to NAV3 was generated in our laboratory. An anti-EGFR for immunoblotting was from Alexis Biochemicals (Lausanne, Switzerland). Secondary antibodies and Alexa Fluor 488-EGF were from Invitrogen (Eugene, OR). The cDNA of human NAV3 was purchased from OriGene (Rockville, MD).

### Immunohistochemical analysis

Formalin-fixed, paraffin-embedded breast tumors were retrieved from the histopathology files of IPATIMUP and Hospital de Săo Joăo (Porto, Portugal). Analysis was performed using the Envision Detection System (DakoCytomation, Carpinteria, CA). Antigen retrieval was performed by using an ethylenediaminetetraacetic solution of pH 9.0 at 98°C for 20 min. The NAV3 mouse monoclonal antibody clone 149 was incubated overnight at 4°C. After immunostaining, slides were counterstained with Mayer's hematoxylin. Two pathologists independently assessed protein levels and scored them based on staining intensity. Statistical analysis of the data was done using the SPSS suite.

### Migration, invasion and chemotaxis assays

For migration assays, cells (6 × 10^4^ cell/insert) were plated in the upper compartment of a Transwell tray (Corning, Acton, MA). Cell invasion assays were performed using BioCoat Matrigel Invasion Chambers (BD Bioscience, Franklin Lakes, NJ). For 3D invasion, cells were suspended in medium supplemented with 4% serum. Eight-chambered plates (BD Biosciences) were coated with Growth Factor Reduced (GFR) Matrigel™ (BD Bioscience). The cells (5 × 10^3^ cells/ml) were mixed (1:1) with assay medium containing Matrigel (10%). For tracking, cells were seeded (3 × 10^3^ cells/cm^2^) on collagen-coated μ-slide 8-well trays (Ibidi) in full medium without EGF. After overnight incubation, the medium was changed and time-lapse images were taken (3–5 h; 10-min intervals). The positions of cell nuclei were followed using ImageJ. For chemotaxis, cells (3 × 10^6^ cells/ml) were seeded in the main chamber of a chemotaxis slide (Ibidi). As control, time-lapse images of cells were taken (6 h; 15-min intervals) in the absence of EGF. Thereafter, EGF was added to one of the side reservoirs, followed by another 6-h session of time-lapse imaging.

### 3D cultures

MCF10A cells were re-suspended in Dulbecco's modified Eagle (DMEM)/F12 medium supplemented with 2% horse serum, insulin, cholera toxin and hydrocortisone. Eight-chambered plates (BD Biosciences) were coated with 35 μl growth factor-reduced Matrigel (BD Bioscience) per well. The cells were mixed 1:1 with assay medium containing Matrigel (4%) and EGF (20 ng/ml), and 400 μl was added to each chamber. Thereafter, acini were fixed in methanol–acetone, and the slides were blocked in goat serum (10%) containing buffer and processed for microscopy using a spinning disk confocal microscope.

### Metastasis tests in animals

Female CB-17 SCID mice (Harlan Laboratories, Haslett, MI) were either implanted in the fat pad with MDA-MB-231 cells (2.5 × 10^6^ per mouse), or they were injected intravenously (1.5 × 10^5^ per mouse). Mice were sacrificed four (tail vein injected) or 8 weeks (fat pad-injected) after injection, and the lungs and/or tumor size was analyzed. Lung images were acquired, and the numbers of micro-metastases or nodules were quantified. Statistical significance was calculated using Student's *t*-test.

### Measurements of MT dynamic instability

Cells co-expressing mCherry-tubulin and GFP were time-lapse recorded (2 min; 4-s intervals). Tips of individual MTs were tracked using ImageJ. Catastrophe (or rescue) frequencies were calculated as the number of events divided by the time of growth (or shortening). The following parameter set of PlusTipTracker was used: maximum gap length, 12 frames; minimum track length, three frames; search radius range, 5–10 pixels; maximum forward angle, 30°; maximum backward angle, 10°; maximum shrinkage factor, 1.5; and fluctuation radius, one pixel. The statistical comparison of MT growth speed and displacement was performed using a permutation test for means and *P *<* *0.05 as threshold.

### Transfections and RNA interference

Plasmid transfections were performed using Fugene HD (Roche, Mannheim, Germany). Oligofectamine (Invitrogen) and ON-Target SMARTpool were used for siRNA transfection (Dharmacon, Lafayette, CO). The following siRNA sequences were used to deplete NAV3 expression:

1. GCUGUUAGCUCAGAUAUUU
2. CAGGGAGCCUCUAAUUUAA
3. GAGAGGGUCUUCAGAUGUA
4. GGACUUAACCUAUAUACUA

shRNA-hairpins against NAV3 were from Open Biosystems (Thermo Fisher Scientific, Huntsville, AL) and produced in HEK-293T cells. Cells were infected with shRNA-encoding lentiviruses with polybrene (8 μg/ml) and cultured for 4 days in the presence of puromycin (2 μg/ml), prior to RNA extraction and real-time PCR. The following shRNA sequence was used to downregulate NAV3 expression:

5′-CCGG-GCCTTGTGAATAGATCGCTTT-CTCGAG AAAGCGATCTATTCACAAGGC-TTTTTG-3′.

### Quantitative PCR

RNA was isolated using the PerfectPure kit (5 Prime GmbH, Hamburg, Germany) and reverse transcribed with the High Capacity cDNA Reverse Transcription kit (Applied Biosystems, Foster City, CA). Real-time PCR analysis was performed using SYBR Green I (Applied Biosystems) in triplicates, and the results were normalized to β2-microglobulin.

### Immunofluorescence analyses

Cells were washed, permeabilized for 3 min using 0.05% Triton X-100 in 3% paraformaldehyde (PFA) and fixed with 3% PFA. For MT staining, cells were fixed in methanol for 20 min at −20°C. After blocking in 1% albumin, cells were labeled with antibodies. Fluorescent images were captured using an Olympus IX71 microscope equipped with a CCD camera (Cool SNAP HQ, Photometrics, Tucson, AZ) and controlled by a DeltaVision system (Applied Precision, Issaquah, WA). Images were processed using the SoftWorx suite and the ImageJ software.

### ImageStream analysis

Cells were stimulated with Alexa Fluor 488-EGF (20 ng/ml; 10 min), acid-washed and incubated at 37°C. Thereafter, cells were trypsinized, fixed with PFA (3%) and fluorescent signals analyzed using the ImageStreamX instrument (Amnis Corp., Seattle, WA). Data were analyzed using the IDEAS 4.0 software.

### Quantitative analyses of cellular motility

A MATLAB script was used to load the acquired experimental data for each cell in terms of position {*x*_*i*_, *y*_*i*_} in the plane for each *i*^th^ time interval. Below we will denote the length of the *i*^th^ step as *l*_i_: 

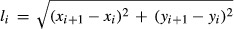
1

Thus, the total length of each walk, up to the *j*^th^ step, can be computed as: 

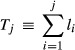
2Note that the maximal total length is *T* ≡ *T*_30_. In addition, the end-to-end distance may be defined as *D*_1,*j*_: 

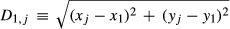
3

Note that the total end-to-end distance for all recorded steps is *D* ≡ *D*_1,30_. Hence, the distribution of step sizes can be computed for each treatment using the respective normalized histograms and the probability distribution function (pdf). For each number of steps from 1,…,30, at any point along the trajectories, we evaluated the average end-to-end distance and average length using two nested loops. The data were then plotted using logarithmic axes. In addition, we characterized the type of walk by assuming that for purely random walks we expect that for large *j*:

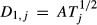
4where A is the order of the mean step size. On the other hand, for a directional persistent walk, we expect that

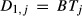
5

where again *B* is proportional to the mean step size. By plotting log *D*_1,*j*_ as a function of log *T*_*j*_, we expect to find a linear plot with slope ½ and intercept log *A* for a random walk, whereas the slope should be unity with intercept log *B* for a persistent walk.

### Real-time impedance and BrdU incorporation assays

Measurements of cell invasion and proliferation were recorded by using the RTCA-xCELLigence System (Roche Diagnostics, Mannheim, Germany). Gold microelectrodes E-16 (cell proliferation) and CIM plates (invasion assays) were used. For the BrdU incorporation assay, cells on coverslips were starved (24 h) and labeled with BrdU (30 min), followed by fixation and staining using a kit from Roche Diagnostics GmbH. Nuclei were stained with DAPI, and cells were visualized using a Nikon Eclipse 90i microscope.

### MT co-sedimentation assay

Cell lysates were cleared by centrifugation (10,000 *g*; 20 min), and the supernatants were further cleared (100,000 *g*; 30 min). Phosphocellulose-purified tubulin (30 μM) was assembled into MTs by incubation in PEM buffer (100 mM Pipes, pH 6.9, 1 mM MgSO_4_, 1 mM EGTA) supplemented with 1 mM GTP. Taxol (30 μM) was added, and tubulin was incubated for 10 min at 37°C, before the addition of pre-cleared supernatants (25 μl). Mixtures were incubated at 37°C for 10 min and then layered on top of a 50 μl cushion (60% glycerol in PEM buffer). Thereafter, MTs were precipitated (40,000 *g*; 30 min). Supernatant fractions were collected, and pellets were dissolved in electrophoresis sample buffer prior to gel loading.

### Study approval by ethical committees

All animal studies were reviewed and approved by the Weizmann Institute's Animal Care and Use Committee (IACUC) and by the Israel Council for Experiments in Animals for compliance with the Israeli animal welfare law, and compliance with the 3R principles and NRC guidelines. Altogether, we used 125 female CB-17 SCID mice (5 weeks old, from Harlan), which were normally housed and provided with food and water *ad libitum*. The retrospective immunohistochemical work was performed in accordance with the Portuguese national regulatory law of accessing tumor bank material for research purposes. The formalin-fixed, paraffin-embedded breast tumors were retrieved with institutional board approval from the Institute of Molecular Pathology and Immunology of the University of Porto and Hospital de Săo Joăo Porto, in Porto, Portugal.

## Supporting Information

Supplementary Figure Legends

Source Data for Supplementary Figure S7B

Review Process File

Source Data for Figure 1D

Source Data for Figure 6C and Supplementary Figure S6B
