# Supplementary figures and images for: Navigator-3, a modulator of cell migration, may act as a suppressor of breast cancer progression
Source: EMBO Mol Med. 2015 Feb 12;7(3):299–314. doi: 10.15252/emmm.201404134 (PMC4364947; doi:10.15252/emmm.201404134)

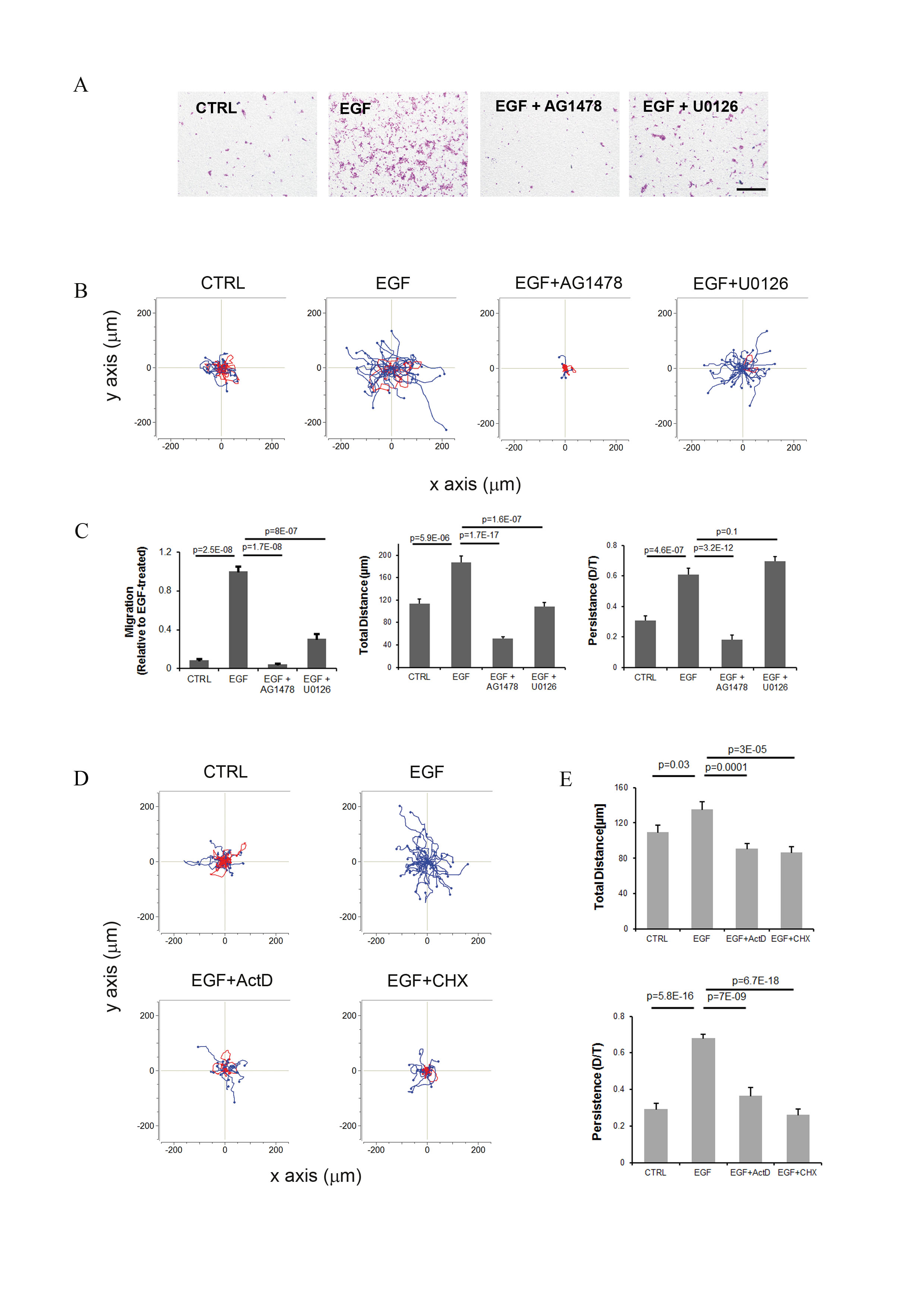

Supplement: Supplementary file 1 [file emmm0007-0299-sd1.tif]

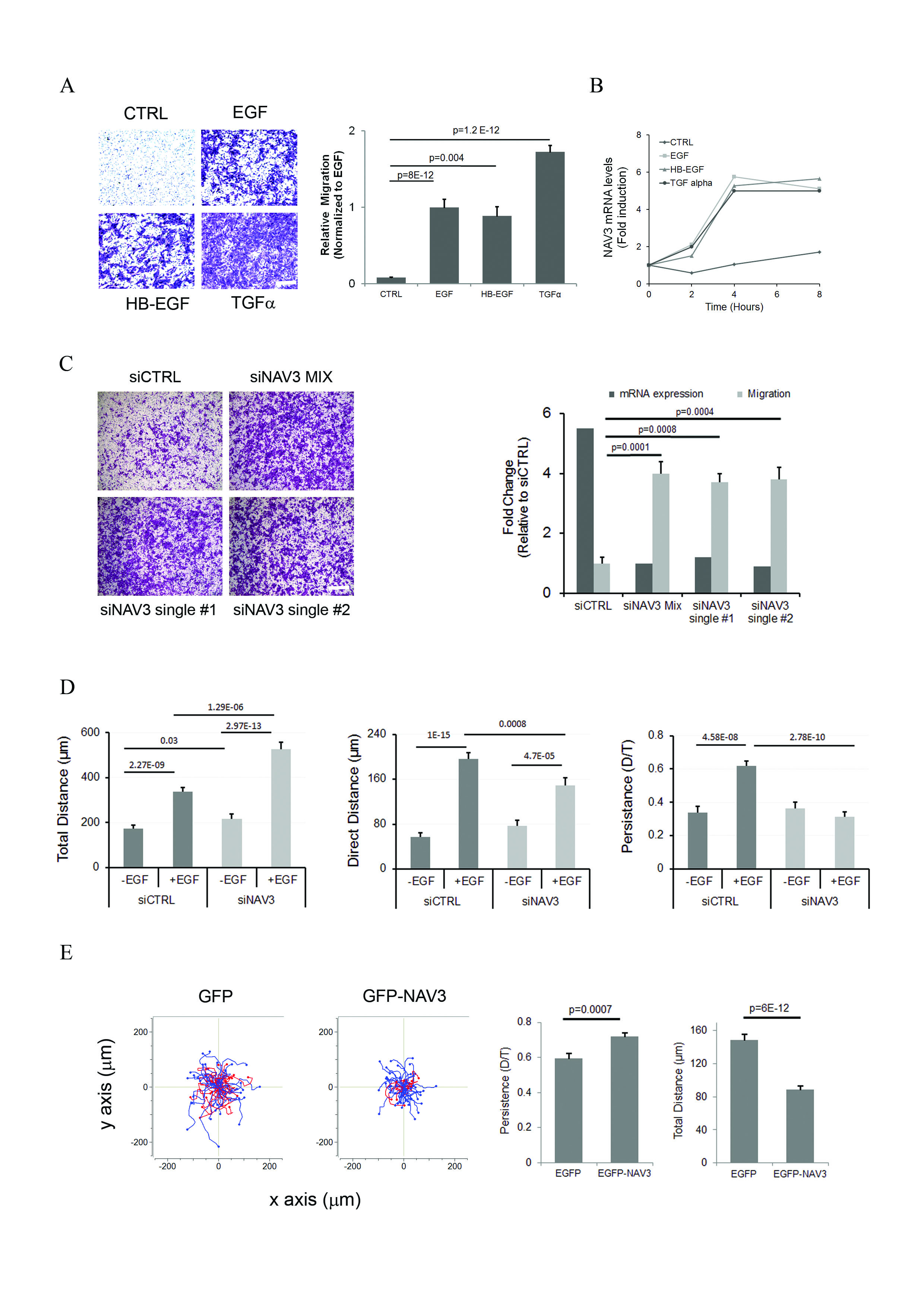

Supplement: Supplementary file 2 [file emmm0007-0299-sd2.tif]

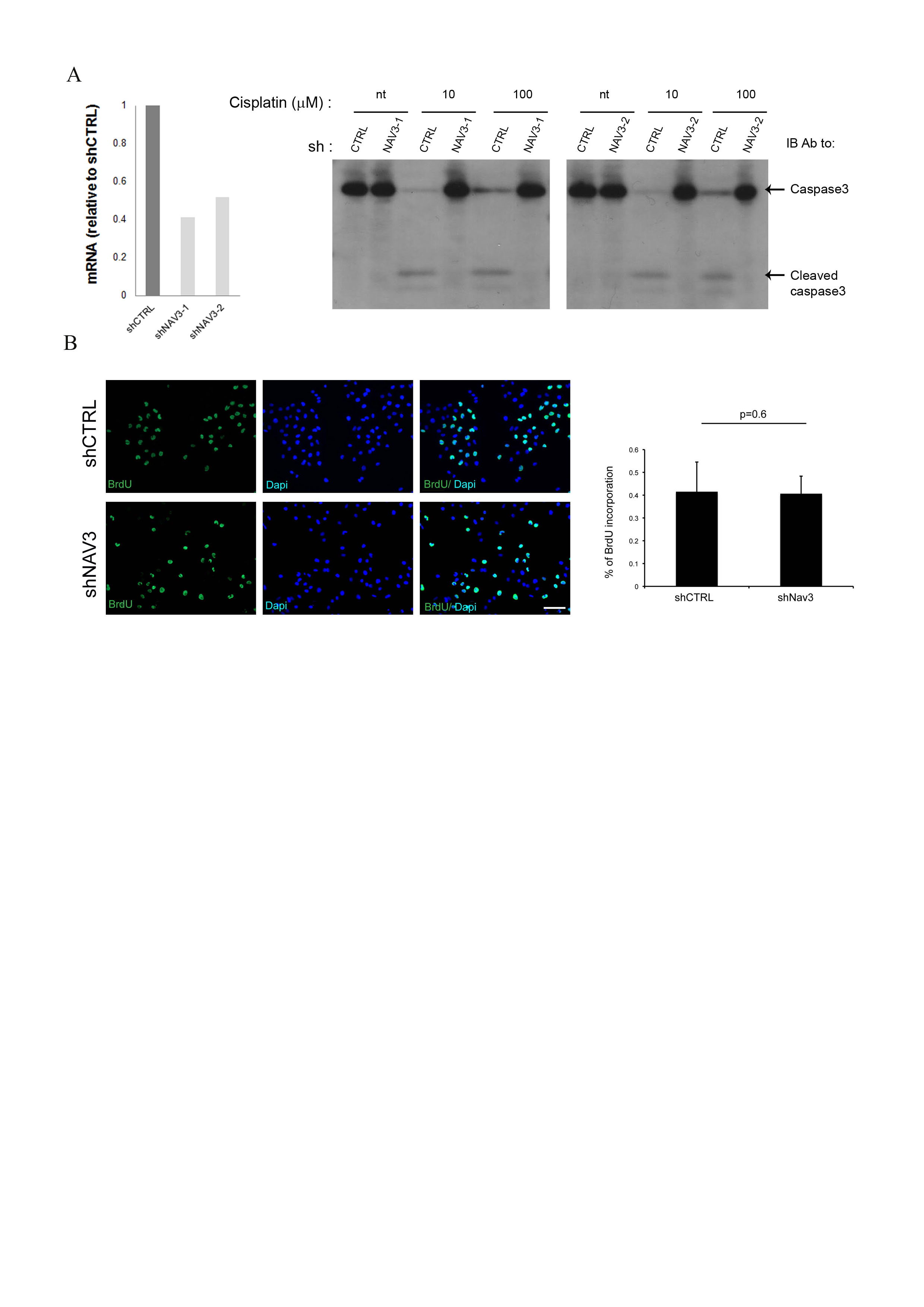

Supplement: Supplementary file 3 [file emmm0007-0299-sd3.tif]

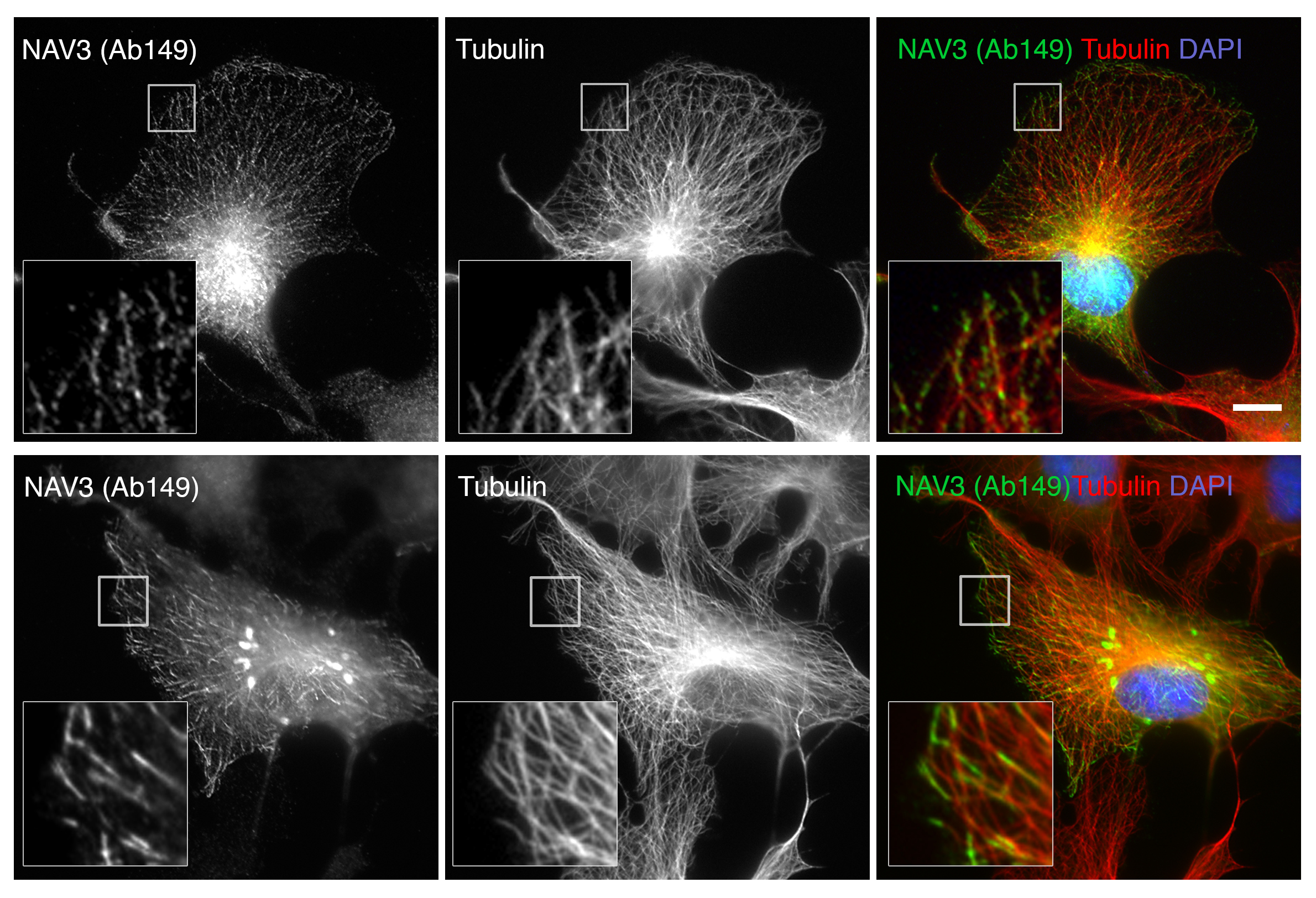

Supplement: Supplementary file 4 [file emmm0007-0299-sd4.tif]

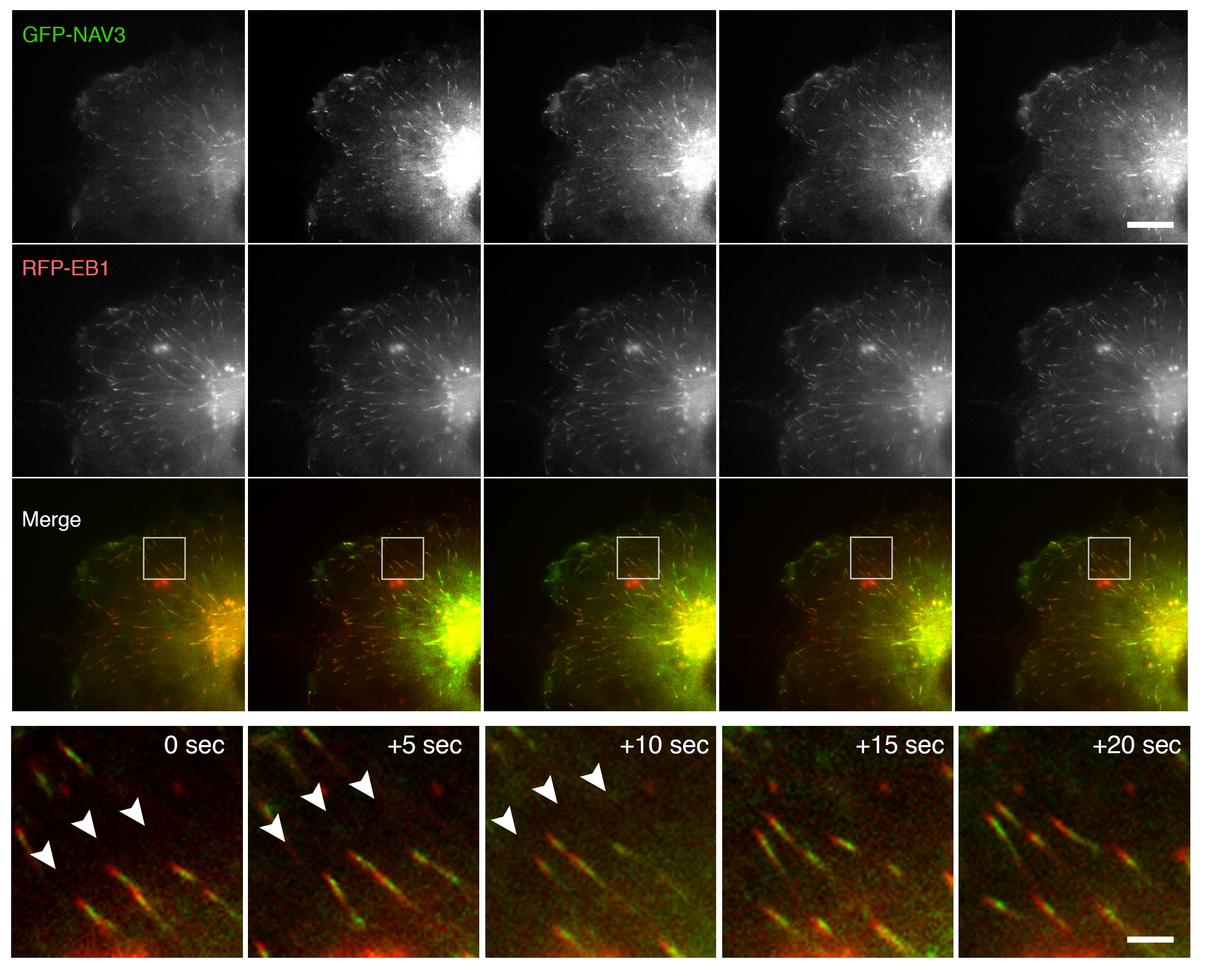

Supplement: Supplementary file 5 [file emmm0007-0299-sd5.tif]

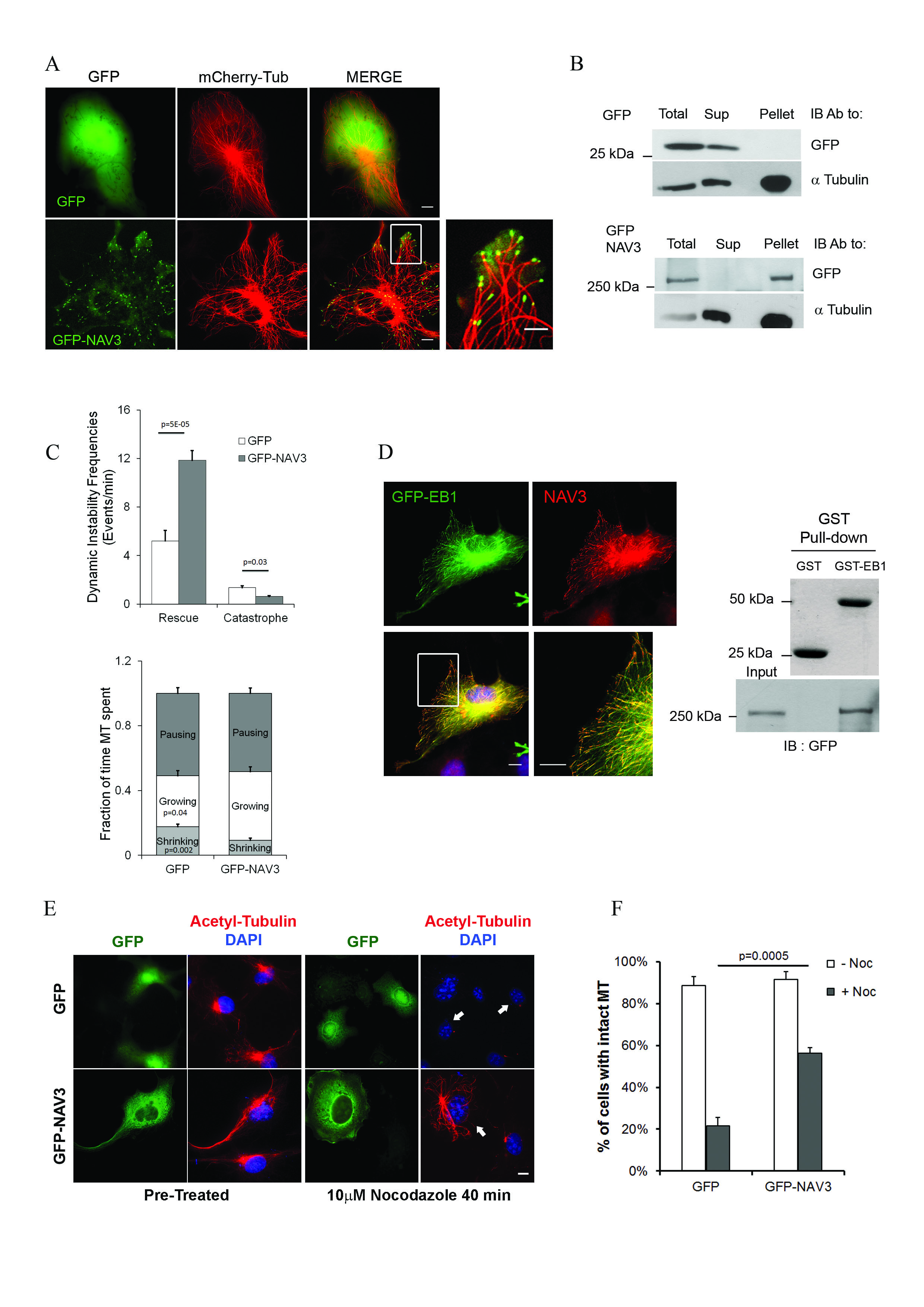

Supplement: Supplementary file 6 [file emmm0007-0299-sd6.tif]

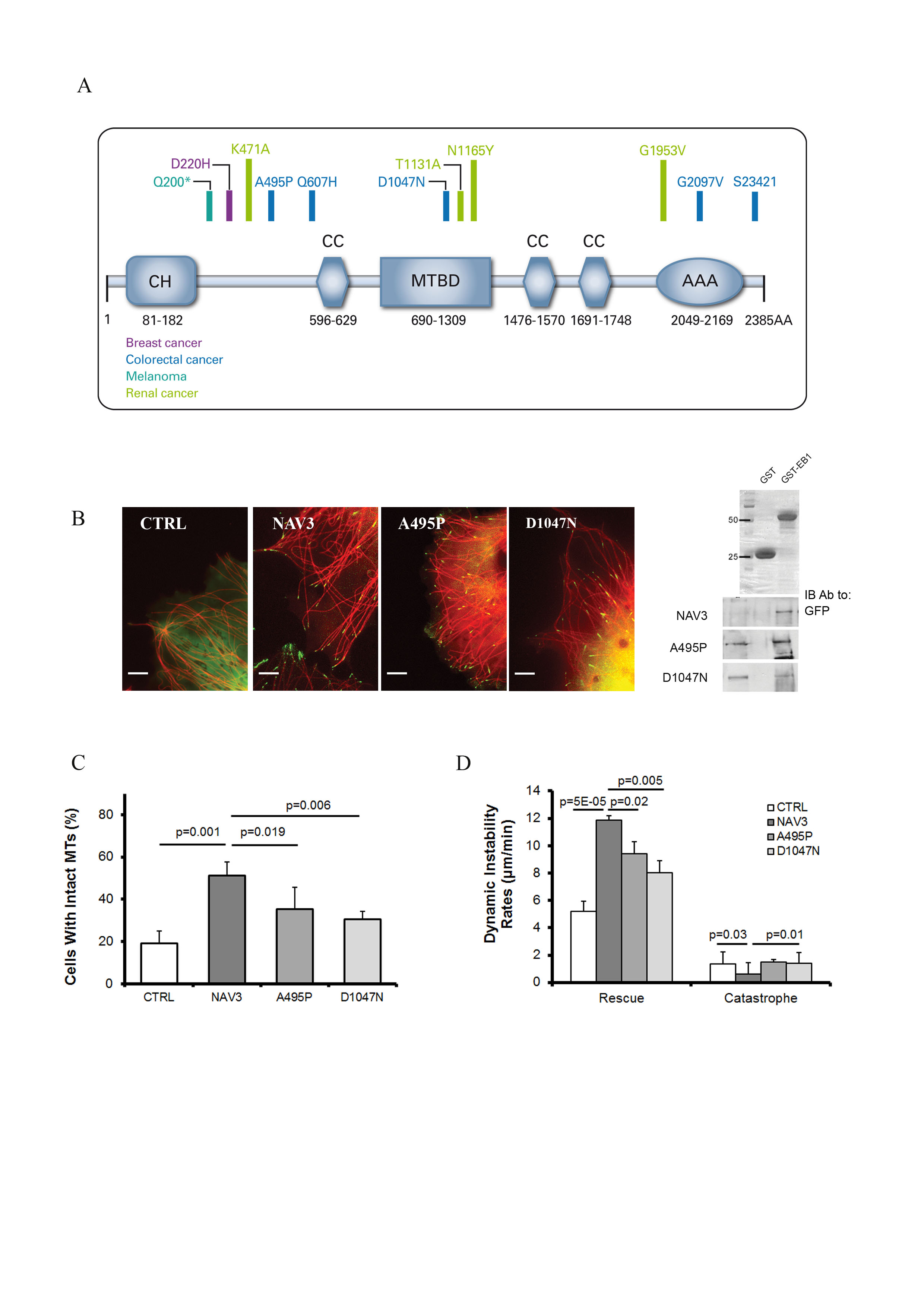

Supplement: Supplementary file 7 [file emmm0007-0299-sd7.tif]

Source File Supplementary Figure 7B

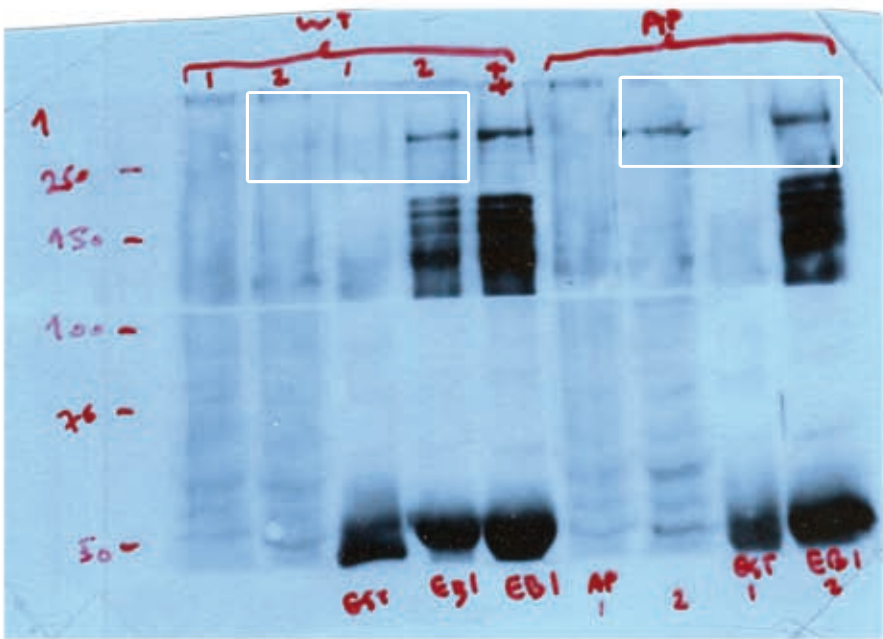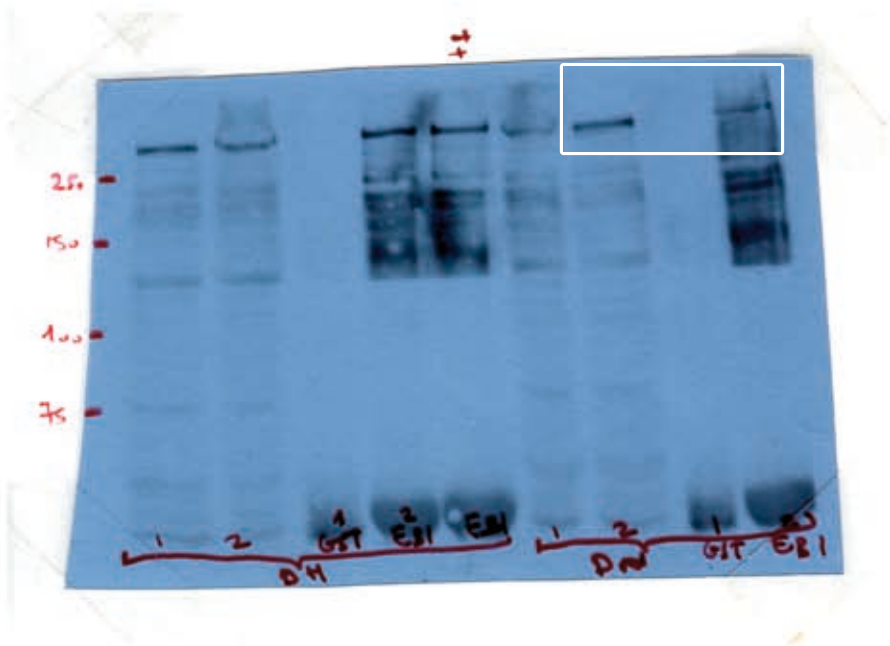

Supplement: Supplementary file 15 [file emmm0007-0299-sd15.pdf]

Source File Figure 1D

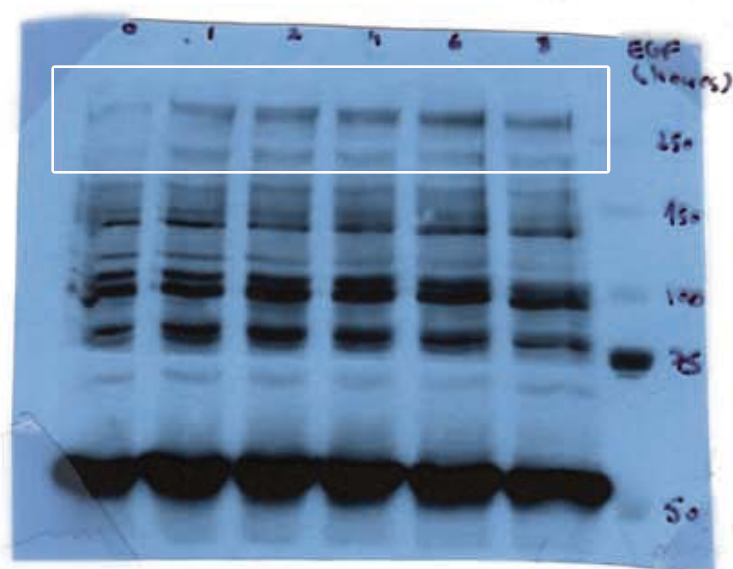

IB:NAV3

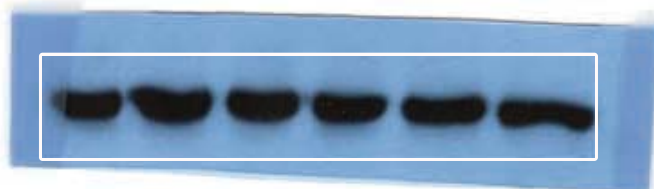

$\alpha$ -tubulin  
lower exposure

Supplement: Supplementary file 17 [file emmm0007-0299-sd17.pdf]

Source File Figure 6C

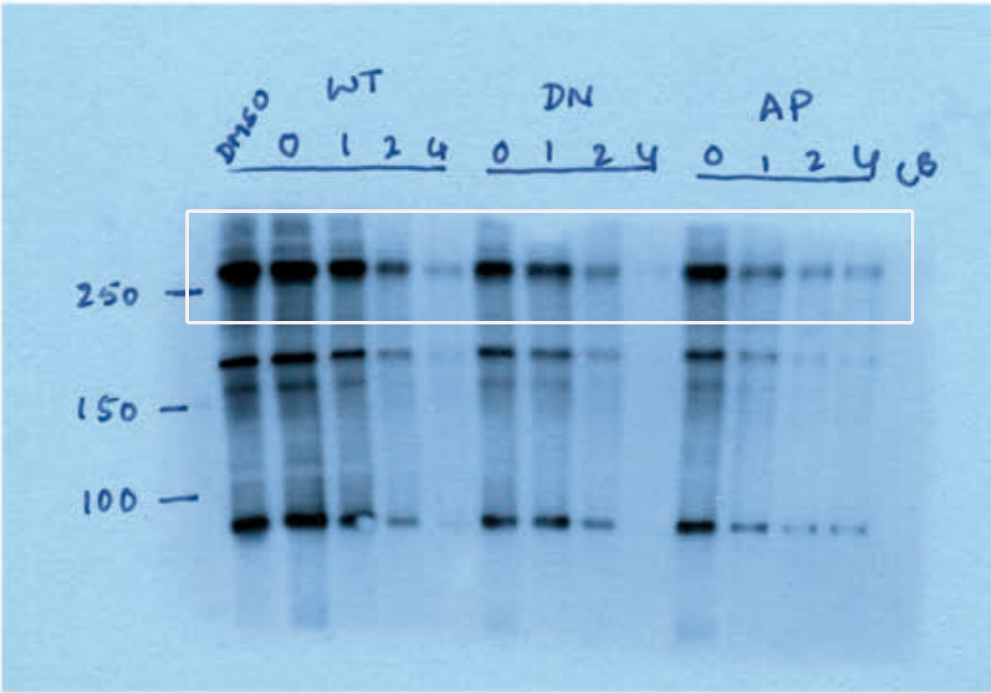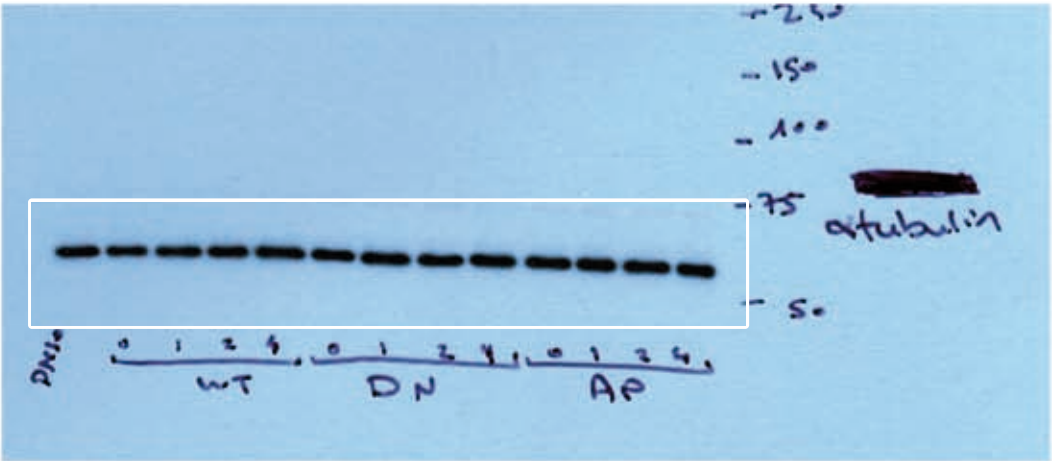

Source File Supplementary Figure 6B

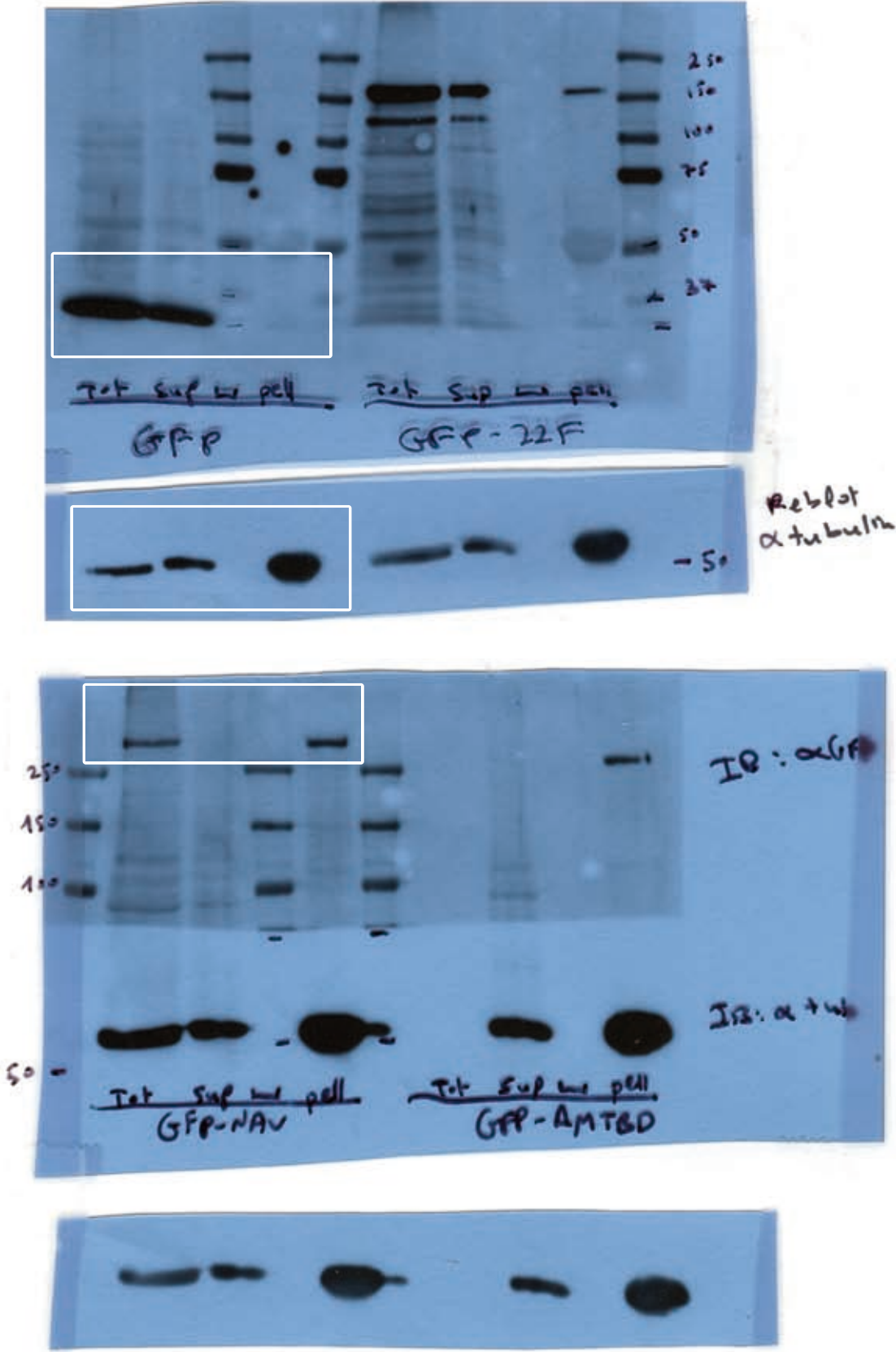

Supplement: Supplementary file 18 [file emmm0007-0299-sd18.pdf]
