# Supplementary material for: Navigator-3, a modulator of cell migration, may act as a suppressor of breast cancer progression
Source: EMBO Mol Med. 2015 Feb 12;7(3):299–314. doi: 10.15252/emmm.201404134 (PMC4364947; doi:10.15252/emmm.201404134)
Supplement: Supplementary file 11 [file emmm0007-0299-sd11.docx]

Supplementary table 1 (three parts): EGF-induced genes of mammary cells

Part 1: Early EGF-induced genes (20-120 min)

| Gene symbol | Gene title |
| --- | --- |
| AQP3 | aquaporin 3 |
| MDFI | MyoD family inhibitor |
| MMP14 | matrix metallopeptidase 14 |
| NUBP2 | nucleotide binding protein 2 |
| PHTF1 | putative homeodomain transcription factor 1 |
| PSMD13 | proteasome (prosome, macropain) 26S subunit, non-ATPase, 13 |
| RAD23B | RAD23 homolog B (S. cerevisiae) |
| SCARB2 | scavenger receptor class B, member 2 |
| SEMA3F | semaphorin 3F |
| SLC39A7 | solute carrier family 39 (zinc transporter), member 7 |
| BUB1 | budding uninhibited by benzimidazoles 1 homolog (yeast) |
| C14orf106 | MIS18 binding protein 1 |
| GNS | glucosamine (N-acetyl)-6-sulfatase |
| HSDL2 | hydroxysteroid dehydrogenase like 2 |
| LRRFIP1 | leucine rich repeat (in FLII) interacting protein 1 |
| NDUFB6 | NADH dehydrogenase (ubiquinone) 1 beta subcomplex, 6 |
| UNC93B1 | unc-93 homolog B1 (C. elegans) |
| Bles03/ C11orf68 | basophilic leukemia expressed protein |
| DLC1 | deleted in liver cancer 1 |
| HES1 | hairy and enhancer of split 1, (Drosophila) |
| IER3 | immediate early response 3 |
| IL6 | interleukin 6 |
| MGMT | O-6-methylguanine-DNA methyltransferase |
| RANBP3 | RAN binding protein 3 |
| SLC35D2 | solute carrier family 35, member D2 |
| AP1S1 | adaptor-related protein complex 1, sigma 1 subunit |
| BAX | BCL2-associated X protein |
| BCL10 | B-cell CLL/lymphoma 10 |
| DNCLI1 | dynein, cytoplasmic 1, light intermediate chain 1 |
| DUSP6 | Dual specificity protein 6 |
| EIF1 | eukaryotic translation initiation factor 1 |
| ENC1 | ectodermal-neural cortex 1 (with BTB-like domain) |
| EPHA2 | EPH receptor A2 |
| GDF15 | growth differentiation factor 15 |
| GPR161 | G protein-coupled receptor 161 |
| H41 | Hystocompatibility 41 |
| IL1B | Interlukin 1B |
| ING2 | inhibitor of growth family, member 2 |
| LSM4 | LSM4 homolog, U6 small nuclear RNA associated (S. cerevisiae) |
| MGC4825 | similar to hypothetical protein MGC4825 |
| NSUN3 | NOP2/Sun domain family member 3 |
| PHLDA1 | pleckstrin homology-like domain, family A, member 1 |
| PPP2R4 | Protein phosphatase 2A activator regulator subunit 4 |
| PRDX2 | Peroxireduxin 2 |
| RPS6KA4 | Ribosomal protein kinase S6 |
| SH2D3A | SH2 domain containing 3A |
| SPRY4 | Sprouty homologue 4 |
| TXNRD1 | Thioreduxin reductase 1 |
| SDHAF1 | Succinate Dehidrogenate assembly factor 1 |
| AP1S1 | adaptor-related protein complex 1, sigma 1 subunit |

Part 2: Late EGF-induced genes (240, 480 min)

| Gene symbol | Gene title |
| --- | --- |
| ARL7 | ADP-ribosylation factor-like 4C |
| BDKRB1 | bradykinin receptor B1 |
| C1orf144 | chromosome 1 open reading frame 144 |
| CABIN1 | calcineurin binding protein 1 |
| CHST11 | carbohydrate (chondroitin 4) sulfotransferase 11 |
| CYP27B1 | cytochrome P450, family 27, subfamily B, polypeptide 1 |
| EGLN1 | egl nine homolog 1 (C. elegans) |
| FOXD1 | forkhead box D1 |
| GFPT1 | glutamine-fructose-6-phosphate transaminase 1 |
| GIT2 | G protein-coupled receptor kinase interacting ArfGAP 2 |
| GPR153 | G protein-coupled receptor 153 |
| GRB10 | growth factor receptor-bound protein 10 |
| HPCAL1 | hippocalcin-like 1 |
| LOC402055 | SRR1 domain containing |
| LYPD3 | LY6/PLAUR domain containing 3 |
| MAPK1 | mitogen-activated protein kinase 1 |
| MGC5139 | unc-119 homolog B (C. elegans) |
| NAV3 | neuron navigator 3 |
| ODC1 | ornithine decarboxylase 1 |
| PLEK2 | pleckstrin 2 |
| POPDC3 | popeye domain containing 3 |
| PTHLH | parathyroid hormone-like hormone |
| RAB22A | RAB22A, member RAS oncogene family |
| RFC1 | replication factor C (activator 1) 1, 145kDa |
| RHOF | ras homolog gene family, member F (in filopodia) |
| SGNE1 | secretogranin V (7B2 protein) |
| SH2D2A | SH2 domain protein 2A ( T cell specific adapter protein) |
| SMTN | Smoothelin |
| STC1 | stanniocalcin 1 |
| TIMM10 | translocase of inner mitochondrial membrane 10 homolog (yeast) |
| TMEM22 | transmembrane protein 22 |
| TRIB3 | tribbles homolog 3 (Drosophila) |
| VEGF | vascular endothelial growth factor A |
| VEGFC | vascular endothelial growth factor C |
| SLC7A11 | Solute carrier family 7 member 11 |
| PLAU | Plasminogen activator, urokinase1 |
| MICAL2 | Microtubule associated monoxygenase calponin and LIM domain containing 2 |
| ITSN1 | Intersectin1 (SH3 domain) |
| DUSP4 | Dual specificity phosphatase 4 |
| PTAFR | Platelet-activating factor receptor |
| SOCS2 | Suppressor of cytokine signaling 2 |
| STK17A | Serine/threonine kinase 17A |
| TNFRSF12A | Tumor necrosis factor superfamily, member 12A |
| ATPBD1B/GPN2 | GPN-loop GTPase 2 (ATP BD 1 family, member B) |
| INSIG1 | Insulin induced gene 1 |
| TUBB2 | Tubulin beta 2A |

*Genes highlighted in blue were selected for further analysis

Part 3: p-Values for significance presented in Figure 1B
