# Supplementary material for: Navigator-3, a modulator of cell migration, may act as a suppressor of breast cancer progression
Source: EMBO Mol Med. 2015 Feb 12;7(3):299–314. doi: 10.15252/emmm.201404134 (PMC4364947; doi:10.15252/emmm.201404134)
Supplement: Supplementary file 12 [file emmm0007-0299-sd12.docx]

Supplementary Table 2: Correlation between NAV3 expression and disease parameters

| NAV3 correlation to: | Correlation | *p*-Value |
| --- | --- | --- |
| Histological grade (I,II,III) | Negative | 2.1E-07 |
| Metastasis (−/+) | Negative | 5.8E-02 |
| EGFR | Negative | 1.9E-06 |
| HER2 | Negative | 4.4E-04 |
| Ki67 | Negative | 6.6E-06 |
| Cytokeratin 5 | Negative | 4.9E-02 |
| Estrogen receptor | Positive | 1.8E-05 |
| PTEN | Positive | 3E-02 |
| pMAPK in tumor | Positive | 3.9E-04 |
| pmTOR | Positive | 1E-02 |
| NFKB | Positive | 1E-02 |
| Tumor size | No correlation | NS |
| Patient age | No correlation | NS |

NS, No statistical significance
