# Supplementary material for: Navigator-3, a modulator of cell migration, may act as a suppressor of breast cancer progression
Source: EMBO Mol Med. 2015 Feb 12;7(3):299–314. doi: 10.15252/emmm.201404134 (PMC4364947; doi:10.15252/emmm.201404134)
Supplement: Supplementary file 13 [file emmm0007-0299-sd13.docx]

Supplementary Table 3: multivariate analysis for IHC

| Disease parameters | NAV3 expression | | | p-Value |
| --- | --- | --- | --- | --- |
|  | Absent/Weak | Moderate | strong |  |
| Molecular subtype  n=323 (100%)  Luminal A  Luminal B  HER2+  Basal | 84 (37%)  26 (72.2%)  19 (61.3%)  23 (79.3%) | 85 (37.4%)  8 (22.2%)  11 (35.5%)  5 (17.2%) | 58 (25.6%)  2 (5.6%)  1 (3.2%)  1 (3.4%) | 4.2 E-07 |
| Histological grade  n=322 (99.7%)  Low (I)  Intermediate (II)  High (III) | 20 (28.6%)  54 (40.6%)  78 (65.6%) | 23 (32.9%)  53 (39.8%)  33 (27.7%) | 27 (38.6%)  26 (19.5%)  8 (6.7%) | 2.1 E-07 |
| Metastasis  n=323 (100%)  Without  With | 135 (45.2%)  17 (70.8%) | 104 (34.8%)  5 (20.8%) | 60 (20%)  2 (8.4%) | 5.8 E-02 |
| HER2  n=323 (100%)  no overexpression  overexpression | 107 (41.8%)  45 (67.2%) | 90 (35.2%)  19 (28.4%) | 59 (23%)  3 (4.4%) | 4.4 E-04 |
| Estrogen receptor  n=323 (100%)  negative  positive | 42 (70%)  110 (41.8%) | 16 (26.7%)  93 (35.4%) | 2 (3.3%)  60 (22.8%) | 1.8 E-05 |
| EGFR  n=317  Absent/Weak  Moderate/strong | 132 (27.6%)  18 (90%) | 106 (35.7%)  1 (5%) | 59 (19.9%)  1 (5%) | 1.9 E-06 |
