# Supplementary material for: Navigator-3, a modulator of cell migration, may act as a suppressor of breast cancer progression
Source: EMBO Mol Med. 2015 Feb 12;7(3):299–314. doi: 10.15252/emmm.201404134 (PMC4364947; doi:10.15252/emmm.201404134)
Supplement: Supplementary file 14 [file emmm0007-0299-sd14.docx]

**Legends to Supplementary Figures**

**Supplementary Figure S1: Pharmacology of EGF-induced mammary cell migration**

**(A)** MCF10A cells were seeded in migration chambers (6×10^4^ cells/well) in the absence or presence of EGF (10 ng/ml), and the indicated inhibitors (10 µM). Cells that crossed the filter after 18 hours were stained and photographed.

**(B)** MCF10A cells were cultured on collagen-coated 8µm slides (from Ibidi), in the absence of EGF. Following attachment, the growth medium was replaced with inhibitor-containing medium and cell movements were tracked using time-lapse microscopy. Shown are rose plot trajectories; red color indicates tracks with low migration persistence (D/T<0.3).

**(C)** Quantification of migration (from B), including total distance (T) and persistence (D/T) of the tracks. Means±SEM of 60 cells are shown. Data were pooled from three experiments.

**(D** and **E)** MCF10A cells were cultured on collagen-coated 8µm slides in the absence of EGF. Following attachment, cells were pretreated for 30 min with either chycloheximide (CHX, 1μg/ml) or actinomycin D (2μg/ml). Following addition of EGF and a 24 hour incubation, cell movements were recorded and tracked. Results are depicted as mean and SEM based on 60 cells (data was pooled from three experiments). P values are indicated *(*two-tailed student`s t-test).

**Supplementary Figure S2: Inducible NAV3 expression regulates mammary cell migration**

(**A**) MCF10A cells were cultured for 18 hours in migration chambers (6×10^4^ cells/well) containing medium supplemented with the indicated growth factor (10 ng/ml) or with serum (5%; CTRL). Cells that reached the filter’s bottom were stained and the respective area coverage was quantified. Relative migration signals were normalized to EGF.

(**B**) Starved MCF10A cells were stimulated with serum or with the indicated growth factors for the indicated time intervals. Thereafter, cells were lysed and total RNA was prepared and subjected to quantitative PCR using NAV3-specific primers.

(**C**) MCF10A cells were transfected with the following siRNA oligonucleotides: control oligonucleotides, two oligonucleotides targeting non-overlapping regions of the NAV3 transcript, as well as a mixture of four oligonucleotides (number 1 and number 2, and two additional oligonucleotides). Forty-eight hours following transfection, cells were transferred into migration chambers and cultured for additional 18 hours. Cells that reached the filter’s bottom were stained and the respective area coverage was quantified. Alternatively, transfected cells were lysed and total RNA was subjected to quantitative RT-PCR using NAV3-specific primers. Relative cell migration (mean±S.D. of three experiments) and relative NAV3 mRNA levels are depicted as fold-change (normalized to siCTRL).

(**D**) Quantification of migration parameters of the tracks presented in Figure 1E. Depicted are means±SEM based on 60 cells and three experiments. P values are indicated.

(**E**) MCF10A cells were transfected with either EGFP or EGFP-NAV3. Forty-eight hours later, cells were tracked and their trajectories were quantified for persistence and for total distance. Red colored tracks indicate persistence values (D/T) smaller than 0.6. Results are depicted as mean±SEM based on 60 cells (data was pooled from three experiments). P values are indicated (two-tailed student`s t-test).

**Supplementary Figure S3: Silencing of NAV3 retards apoptosis of mammary cells minimally affects proliferation rates**

(**A**) MCF10A cells stably expressing either shControl or the indicated shNAV3 were tested for NAV3 expression using RT-PCR (left panel). In addition, the same cells were treated for 24 hours with the indicated concentrations of cisplatin (nt, no treatment), followed by immunoblot (IB) analysis of whole cell extracts with antibodies to caspase-3. Arrows mark the locations of intact and cleaved forms of caspase 3.

(**B**) The indicated derivatives of MDA-MB-231 cells were grown on cover slips and starved for 24 hours, followed by incubation with BrdU, fixation and staining with DAPI. BrdU incorporation into DNA was measured by determining the ratio of BrdU- to DAPI-stained nuclei. Data represent mean and standard errors calculated from at least 15 non-overlapping photomicrograph fields (>500 nuclei). The *p*-value of two-tailed student’s t test showed no statistically significant difference.

**Supplementary Figure S4: NAV3 localizes to the growing ends of microtubules**

Shown are images of COS-7 cells transiently expressing a FLAG-NAV3 protein. The cells were probed using an anti-NAV3 antibody (monoclonal antibody clone 149) and an anti-tubulin antibody. Thereafter, cells were counterstained with DAPI, to visualize nuclei. Rectangles mark the enlarged areas, which are shown in the bottom left corners. Images of two cells are shown. Bar, 5 μm.

**Supplementary Figure S5: NAV3 co-localizes with EB1 to the ends of microtubules**

Shown are successive time-lapse images (5 seconds apart) of live COS-7 cells co-expressing GFP-NAV3 (green) and RFP-EB1 (red). The rectangles mark the enlarged areas depicted in the bottom row. The arrowheads mark individual microtubules. Note that the red channel was recorded first (for one second), followed by a short interval of 1.5 seconds and a similar recording from the green channel. For this reason, red-yellow-green triads represent co-localization. Bar, 10 μm.

**Supplementary Figure S6: Expression of NAV3 augments MT stability**

**(A**) Live images of COS-7 cells co-expressing either GFP (upper panel) or GFP-NAV3 (lower panel), along with mCherry-tubulin. The rectangle marks the enlarged area depicted in the far right panel. Bars, 10 and 5μm.

**(B**) Whole extracts of HEK-293 cells, previously transfected with a GFP-tagged NAV3 plasmid (or GFP alone), were subjected to a microtubule co-sedimentation assay. Subsequently both the pellet and the supernatants were processed for immunoblotting using anti-GFP and anti-α-tubulin antibodies.

**(C**) COS-7 cells were co-transfected with plasmids encoding mCherry-Tubulin and either EGFP or EGFP-NAV3. Dynamic MTs at the lamella/lamellipodium of co-transfected cells were tracked. Rates of dynamic instability (rescue and catastrophe) and the percentages of time MTs spent growing, shrinking or pausing, were calculated. Results are depicted as mean±SEM from 25 MTs (~5 MTs per cell; 5 cells). P values were analyzed using 1-way ANOVA (Bonferroni *post hoc* correction).

**(D**) Images of COS-7 cell co-expressing FLAG-NAV3 and GFP-EB1, and immunostained with an anti-NAV3 antibody and DAPI. The rectangle marks the enlarged area depicted in the far right panel. Bars, 10 and 5μm. The right hand panels present a GST pull-down experiment. Whole extracts of HEK-293 cells, previously transfected with an EGFP-NAV3 plasmid, were subjected to pull-down with beads coated with GST or GST-EB1. The upper panel shows protein staining and the lower panel shows immunoblotting (IB) with an antibody to GFP.

**(E** and **F**) COS-7 cells ectopically expressing either GFP or GFP-NAV3 were treated for 40 minutes with Nocodazole (10 μM), prior to staining with an anti-acetyl tubulin antibody (red) and with DAPI (blue). White arrows indicate transfected cells. Bars, 10 μm. The fraction of transfected cells with intact MTs was analyzed. Approximately 100 transfected cells were analyzed per condition. Error bars show mean±S.D from three experiments.

**Supplementary Figure S7: *NAV3* mutant cancer alleles fail to stabilize microtubules**

(**A**) A scheme showing the locations of all known mutations within the coding regions of the human NAV3 gene. Note the color code of each mutation, according to the tissue of origin. Numbers indicate amino acids (AA). Shown are the calponin homology (CH) domain, the microtubule-binding domain (MTBD), the coiled coil (CC) regions and the triple A domain, which functions as an ATPase.

(**B**) Left: Images of COS-7 cells co-expressing mCherry-tubulin and the indicated GFP-tagged forms of NAV3. Bars, 5 μm. Right: Extracts of HEK-293 cells, previously transfected with the indicated GFP-NAV3 constructs, were subjected to a pull-down assay using beads coated with GST or GST-EB1 (upper panel: protein staining; lower panels: immunoblotting for GFP).

(**C**) COS-7 cells ectopically expressing either GFP (CTRL), NAV3, or the indicated mutants were treated for 40 minutes with Nocodazole (10 μM), prior to staining with an anti-acetyl tubulin antibody. The fraction of transfected cells with intact MTs is presented. Approximately 100 transfected cells were analyzed per construct. Error bars show mean±S.D. (three experiments).

**(D**) COS-7 cells were co-transfected with plasmids encoding mCherry-tubulin and the indicated constructs. Rates of dynamic instability were calculated by tracking MTs.
