# Supplementary material for: Navigator-3, a modulator of cell migration, may act as a suppressor of breast cancer progression
Source: EMBO Mol Med. 2015 Feb 12;7(3):299–314. doi: 10.15252/emmm.201404134 (PMC4364947; doi:10.15252/emmm.201404134)
Supplement: Supplementary file 16 [file emmm0007-0299-sd16.pdf]

Navigator-3, a modulator of cell migration, may act as a suppressor of breast cancer progression

Hadas Cohen-Dvashi, Nir Ben-Chetrit, Roslin Russell, Silvia Carvalho, Mattia Lauriola, Sophie Nisani, Maicol Mancini, Nishanth Nataraj, Merav Kedmi, Lee Roth, Wolfgang Köstler, Amit Zeisel, Assif Yitzhaki, Jacques Zylberg, Gabi Tarcic, Raya Eilam, Yoav Wigelman, Rainer Will, Sara Lavi, Ziv Porat, Stefan Wiemann, Sara Ricardo, Fernando Schmitt, Carlos Caldas and Yosef Yarden

Corresponding author: Yosef Yarden, The Weizmann Institute of Science

Review timeline:

Submission date:	02 April 2014
Editorial Decision:	06 May 2015
Revision received:	15 August 2014
Editorial Decision:	12 September 2014
Revision received:	12 December 2014
Editorial Decision:	18 December 2014
Revision received:	25 December 2014
Editorial Decision:	07 January 2015
Revision received:	11 January 2015
Accepted:	13 January 2015

Transaction Report:

(Note: With the exception of the correction of typographical or spelling errors that could be a source of ambiguity, letters and reports are not edited. The original formatting of letters and referee reports may not be reflected in this compilation.)

Editor: Roberto Buccione

1st Editorial Decision 06 May 2015

Thank you for the submission of your manuscript to EMBO Molecular Medicine. We have now heard back from the three Reviewers whom we asked to evaluate your manuscript.
You will see that while one Reviewer is more supportive of your work, two are negative with varying degrees. The concerns expressed prevent us from considering publication at this time.

I will not dwell into much detail, as the evaluations are self-explanatory. I would like, however, to mention the main points.

Reviewer 1 feels that the manuscript does not improve our understanding of the function of Nav 3 nor does it clearly explain how Nav3 impacts cancer cell dissemination.

Reviewer 2, while more appreciative of the study, does raise doubts on its translational relevance (which of course is quite important for our title) and suggests further experimentation on a suitable breast cancer model.

Reviewer 3, similarly to reviewer 1, finds mechanistic insight and novelty to be insufficient. S/he also notes that how Nav3 impacts cancer progression remains unclear. This Reviewer also provides a detailed list of critical issues, some technical, some mechanistic, which partially overlap with

certain Reviewer 1's concerns. There are also requests for further clarification.

Extensive mechanistic insight is not an absolute requirement for EMBO Molecular Medicine when the clinical implications and translational relevance are striking. In this case however, the Reviewers, in aggregate, challenge the manuscript on both counts. To be considered further, your manuscript would have to be substantially developed in a mechanistic sense, both concerning the basic function of NAV3 and its role in cancer progression. Ideally, it would be also advisable to extend your experimentation to a more advanced breast cancer model.

All considered, while publication of the paper cannot be considered at this stage, we would be prepared to consider a substantially revised submission as outlined above, and that acceptance of the manuscript will entail a second round of review.

Since the required revision in this case appears to require a significant amount of time, additional work and experimentation and might be technically challenging, I would understand if you chose to rather seek publication elsewhere at this stage. Should you do so, we would welcome a message to this effect.

As you know, EMBO Molecular Medicine has a "scooping protection" policy, whereby similar findings that are published by others during review or revision are not a criterion for rejection. However, I do ask you to get in touch with us after three months if you have not completed your revision, to update us on the status. Please also contact us as soon as possible if similar work is published elsewhere.

***** Reviewer's comments *****

Referee #1 (Comments on Novelty/Model System):

The role of Nav3 (navigator 3) is investigated in breast cancer cell migration, invasion and metastasis. Navigator proteins had previously been shown to be microtubule tip binding proteins that are involved in process extension and neuronal pathfinding. Here, the authors confirm that Nav3 levels can affect microtubule dynamics and show that Nav3 is induced by EGFR stimulation and affects migration and invasion. While the authors reveal interesting effects of depleting or overexpressing Nav3, this study adds little to our mechanistic understanding of how Nav3 functions in cells. Nav3 doesn't seem to localize to microtubules, but it affects their dynamics. It isn't clear how Nav3 affects microtubules though or whether the microtubule effects account for the invasion and migration effects. The authors show that depletion of Nav3 leads to increased metastatic dissemination in both a tail vein injection and a mammary fat pad implantation model. They also implicate Nav3 in human cancer and show that Nav3 downregulation correlates with poor survival in breast and lung cancer patients. While the authors provide compelling evidence that loss of Nav3 function promotes cancer spread, the functional studies do not give a clear picture of why or how. Overall, this study reveals that Nav3 expression has a complex relationship with cell migration and invasion and with cancer spread. The authors make a lot of observations, but they seem difficult to tie together.

Referee #1 (Remarks):

Specific Comments:

1) Silencing Nav3 reduces an EGF-stimulated increase in directional persistence, but how? Nav3 depletion also seems to increase sustained EGFR signaling (Fig 5C), so how does this work?
2) It also seems hard to explain how reduced chemotaxis to EGF of Nav3 depleted cells could lead to increased invasion and metastatic dissemination.
3) I didn't see the point of the mathematical model- what did it reveal that the data didn't already show? Perhaps this needs to be explained more clearly.
4) In the assays where the cells migrate through filters, how much of the effects seen could be due to

cell proliferation/death changes? The authors show that loss of Nav3 reduces apoptosis. How does this work? Is this effect only observed in 3D?
5) Ectopic expression of Nav3 stabilizes microtubules and reduces catastrophe rate, but decreases the persistency of migration. This seems counterintuitive. Are these two observations connected in some way?
5) In Figure 4D, the description given in the legend doesn't seem to match the figure.
6) Why are the mutants of Nav3 defective? Do they express at normal levels in cells? Which function have they lost?

Referee #2 (Comments on Novelty/Model System):

I would recommend to the authors to further validate the clinical values of NAV-3 using transgenic models. They may do so for their next paper

Referee #2 (Remarks):

The manuscript submitted by Cohen-Dvashi et al. provides an in-depth analysis of the potential role of navigator-3 (NAV3) as a promoter of directional migration in vitro. The knock-down of NAV-3 in MCF10-A alters the formation of a lumen in a 3D environment. Restoration of the expression NAV-3 in the aggressive MDA-MB-231 mammary adenocarcinoma cell line abrogates invasion in a 3D extracellular matrix and in in vivo metastatic colonization of the lung.
The authors have explored the function of NAV-3 in vitro. Interestingly, NAV-3 localizes with End Binding Proteins at the plus end of dynamic microtubules and contributes to their stabilization through tubulin acetylation. This finding is related to the observation that EGFR signaling is short-lived as compared to its long half-life in NAV-3-depleted cells. A sustained activation of EGFR signaling is then hypothesized to be responsible for the lack of a chemotactic behavior promoting random migration, especially in tumors with activated PI3K.
The authors found that a decreased expression of NAV-3 correlates with poorer survival in breast cancers, and this was associated with some of the 10 breast cancer molecular subtypes identified by Curtis et al.

Comments
It is somewhat counter-intuitive that random migration observed in vitro would necessarily be more effective in the invasion of 3D environments.
The authors should provide a more precise description of NAV-3 mutational status in tumors. The authors mention that is it rarely mutated (page 11).
The authors should comment on the fact that low NAV-3 expression correlates with poor survival only in ER-positive tumors. EGFR receptor signaling seems more clearly associated with triple-negative tumors; these tumors could also express NAV-3, as it is a target of EFGR signaling.
Many genes have been found to correlate with survival but most of them have not been shown to impact tumor progression outside a few cell line models. The authors may consider analyzing classical breast tumors models with a wild-type or deleted NAV-3 background.

Referee #3 (Remarks):

The manuscript by Cohen-Dvashi and collaborators describes that the microtubule + end binding protein NAV3, induced by EGF in the MCF10A breast cancer cell line, inhibits EGF-induced cell velocity, but enhances persistent migration, and invasion and experimental metastasis by the MDA-MB231 cell line. The author claims that these effects might be the consequence of increased

microtubule growth. NAV3 mutants similar to the ones found in cancer are functionally impaired for directional persistence and metastasis formation. As NAV3 is expressed at low levels in breast tumors, and is associated with low survival, the data suggests that NAV3 might be a suppressor of breast cancer progression.

While the study provides lots of interesting evidence linking NAV3 to microtubule stability and cell invasion, it falls short on the molecular mechanisms. NAV proteins are known +TIPs and, as such, were previously associated to microtubule stability and directional migration (of neurons). This study confirms that ectopically expressed NAV3 is a +TIP and shows that it has an impact on microtubule dynamics, favoring microtubule growth. It also shows that NAV3 depletion leads to increased formation of invadopodia, even though no data is provided that support a causal link between decreased microtubule stability and increased number of invadopodia. There is also no demonstrated connection between NAV3-induced microtubule stability and reduced metastasis. Because NAV3 knockdown also induced increased growth of primary tumors, it is not obvious whether the impact on metastasis is due to the effects on tumor growth or on invasion. Very interestingly two NAV3 mutants, that recapitulate mutations found in tumors, are functionally inactive. However, how, at the molecular level, these mutations affects NAV3 activity is not explored.
Thus despite the interest of the data, the study does not provide significant insights into the mechanism of action underlying NAV3 function as a potential suppressor of cancer progression.

Other comments:
1. Transient transfection of siRNA is used throughout the manuscript. The depletion of NAV3 should be shown for every experiment, if possible at the protein level. Similarly the level of NAV3 upon shRNA-mediated depletion must be verified, especially in long term experiments (3D growth, metastasis). This is especially true because cells are sometimes challenged at the same time with EGF which is supposed to increase NAV3 expression and siRNA which will bring down the expression levels. Moreover, it would be nice to validate, at least some of the key results, with another siRNA/shRNA.
2. What is the relative expression level of NAV3 in the various cellular models used in the study: MCF10A, MDA-MB231, HEK293 and COS7? Are MCF10A representative of normal cells with high expression levels? Are MDA-MB231 representative of aggressive breast cancer cells with low NAV3 (which would question the rationale of further down-regulating NAV3 expression)? Are there breast cancer cells lines devoid of NAV3 expression (or expressing NAV3 mutants)? If yes, is there a correlation between expression of NAV3 and migration/invasion efficiency?
3. Is NAV3 also induced by EGF in other cell lines, e.g. the MDA-MB231? And why would pro-oncogenic EGF signaling lead to expression of a suppressor of invasion. ? How does this relate to aggressive breast tumors which express EGFR, but have low NAV3? Inversely is EGFR also induced by shNAV3 in cell lines others than the MDA-MB231. NAV3 appears to be downregulated in a large majority of breast tumors, irrespective of their EGFR status. From the discussion, it is not clear what the model is and whether the authors propose that the effect of NAV3 depletion is necessarily mediated via EGF signaling or not.
Actually, most experiments using MDA-MB231 cells are performed in the absence of EGF (from my understanding of the legends and methods): cell invasion, invadopodia formation, cell proliferation, and microtubule dynamics (except for MDA-MB231 cell persistence and velocity which were unfortunately not tested in the absence of EGF). So it seems that most effects are independent of EGF signaling.
4. In gain of function experiments, expression levels of NAV3 (Fig. S2) and NAV3 mutants (Fig. 6 and S4 and S5) relative to endogenous NAV3 should be shown. The rationale for testing only the D1047N and A495P mutants is not provided. Why are gain of function experiments not performed in MDA-MB231 (which again should have low expression of NAV3)?
5. If the authors state that MCF10A acini expressing shNAV3 are larger (Fig. 2), the data should be quantified. Moreover they also claim that the acini are amorphous. Is this related to an overproliferative or an invasive phenotype? The claim that the observed effect on apoptosis can support an involvement of NAV3 in polarity is surprising. No markers of polarity are used to support this claim. And surprisingly, lumen formation or cell polarization is not observed in control cells, which makes it difficult to evaluate the impact of NAV3 on polarity.
The impact of NAV3 on cell proliferation should have been tested over longer period of time, in 2D and 3D models. Because NAV3 depletion has an effect on primary tumor growth and because it induces larger acini, it is likely to have an effect on proliferation and/or survival.

6. Is endogenous NAV3 capable of tip-tracking? Apparently not, from the Fig. 4C. This should raise questions relative to its mode of action, that deserved to be discussed. While depleting NAV3 decreases acetylated tubulin, does it modify the density or organization of the microtubule network? Does NAV3 interact with acetylated MT (for instance MTs that remain after nocodazole treatment)? Does NAV3 overexpression induce MT bundles, does it induce cell extension as described previously?
7. For the TMA study, it is important to show the validation of the NAV3 antibody for immunohistochemistry.
In Fig. 2B, the number of patients in each subgroup strong, moderate and absent/weak is not specified. In Fig 2C, the number of patients in the subpopulations is not mentioned.
For the large study dataset, why was the study focused on ER+ patients? According to the manuscript, 1471 patients were included in the study, but on Fig. 7D, n=200.
A complete multivariate analysis should have been presented for both studies (as supplementary tables). The analyses suggest a correlation between loss of NAV3 and proliferation, as supported by some data.
8. The description of experiments is not detailed enough and is sometimes confusing. To cite some examples:
Which siRNA is used throughout the manuscript? In the methods section, 4 different siRNA are described, but only one is used in the study, which is not specified. What control is used in siRNA (or shRNA) experiments?
It is often unclear how cells have been challenged: starved, with FCS, with EGF?
In the Transwell assays, it is not clear whether EGF has been added in the bottom chamber or in both chambers, and how cells were stained and migration quantified. It is mentioned at one point that control medium contains 5% serum? Does that mean that the EGF-treated cells are also in 5% serum?
It is mentioned in the legend of Fig. S3 that the MDA-MB231 cells were starved? Why? Were the cells starved in all experiments? Was the Transwell assays performed for MDA-MB231 cells in medium with or without serum? This is not described. Why was the assay not performed in the presence of EGF, like the tracking assay (Fig. 5). Because the assays are not properly described, it is difficult for the reader to understand what events are EGF-dependent and what events are not.
The way statistical analyses were performed is also confusing. Statistics must be presented for all experiments, including the supplementary data. If they are not presented, the authors must explain why. It seems that some experiments were not repeated (e.g. Fig. 1C-E, S3). This must be clearly stated.
The number of cells analyzed per experiment must be specified for all experiments. And the number of times the experiments were repeated (e.g. Fig. 4B, only 8 or 9 cells analyzed? From how many experiments?). For several experiments, it is not clear whether statistics were applied on cells within the same experiments or on independent biological replicates.
For example, for experimental metastasis, how many mice were included per data point? How many replicates? Were statistics applied on total mouse population or on independent biological replicates? The number of lung metastases is very different in the two tail-vein injection experiments: average of 2 in Fig. 3E and around 200 in Fig 6D? Is there an explication for the large difference? The Y axis is labeled differently in each of the three metastasis experiments. Was the data quantified differently? In the metastasis from fat pad, do the indicated numbers (between 2000 and 4000) actually represent the number of metastases?

1st Revision - authors' response 15 August 2014

List of Corrections and New Experimental Results Incorporated into the Revised Manuscript in Response to Comments of the Reviewers

Referee #1 (Comments on Novelty/Model System):

The role of Nav3 (navigator 3) is investigated in breast cancer cell migration, invasion and metastasis. Navigator proteins had previously been shown to be microtubule tip binding proteins that are involved in process extension and neuronal path finding. Here, the authors confirm that

Nav3 levels can affect microtubule dynamics and show that Nav3 is induced by EGFR stimulation and affects migration and invasion. While the authors reveal interesting effects of depleting or overexpressing Nav3, this study adds little to our mechanistic understanding of how Nav3 functions in cells. Nav3 doesn't seem to localize to microtubules, but it affects their dynamics. It isn't clear how Nav3 affects microtubules though or whether the microtubule effects account for the invasion and migration effects. The authors show that depletion of Nav3 leads to increased metastatic dissemination in both a tail vein injection and a mammary fat pad implantation model. They also implicate Nav3 in human cancer and show that Nav3 down regulation correlates with poor survival in breast and lung cancer patients. While the authors provide compelling evidence that loss of Nav3 function promotes cancer spread, the functional studies do not give a clear picture of why or how. Overall, this study reveals that Nav3 expression has a complex relationship with cell migration and invasion and with cancer spread. The authors make a lot of observations, but they seem difficult to tie together.

The present study is the first report demonstrating functional links between EGFR signalling and NAV3, as well as relevance to tumour progression in animal models and in cancer patients. As such, the study opens many mechanistic questions, which we have not dealt with due to either space limitations or because we were unable to completely resolve the underlying processes. Following the critical comments raised by this and the other Referees, we detail in this letter several mechanistic aspects that were added to the revised manuscript. In addition, we present below our current working models, which attempt to tie the random type of migration to oncogenic signalling pathways.

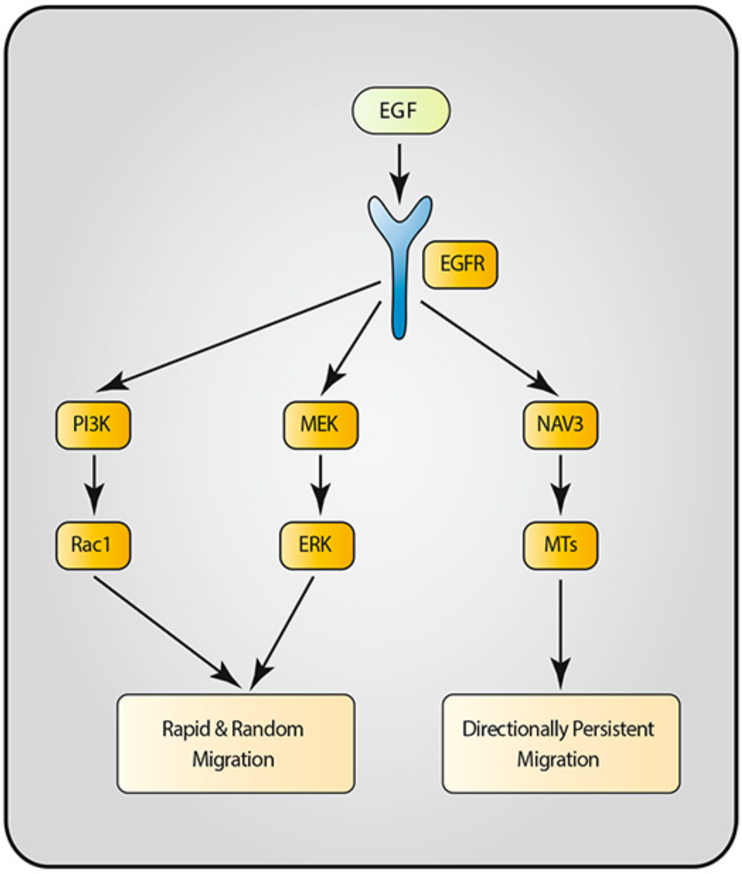

Figure I: ***A scheme depicting signalling pathways putatively controlling each mode of cell migration.*** According to the working model, EGFR signalling simultaneously controls both the directionally persistent mode of cell migration and the more rapid, random type of locomotion. Both a delayed, transcription-based mechanism, and an earlier mRNA-independent process (see Figure II, below), enable up-regulation of NAV3 abundance, thereby they stabilize microtubules (MTs) and favour the chemotaxis-like mode of persistent motility. EGFR's downstream signals that activate the ERK pathway and the

phosphatidylinositol 3-kinase (PI3K) route, which increases Rac1-GTP, collaboratively enhance the random type of migration. This is mediated by the stimulatory effect of Rac1 on actin polymerization and lamellipodia formation through the WAVE-Arp2/3 complex. Importantly, the ERK and PI3K pathways are constitutively active in tumours due to mutations in RAS, BRAF, PI3K and other components, thereby activating the random, metastasis-supporting mode of cell migration. According to our results, this oncogenic mode might be further augmented in certain malignancies in which NAV3 abundance is low due to mutations or other mechanisms. Note that the model is currently supported by some pharmacological and other lines of evidence. Our current efforts, including those presented below, attempt providing additional molecular evidence.

Yet another working hypothesis we currently examine has been driven by the analogy between NAV3 and another, relatively large cytoplasmic protein, the tumour suppressor adenomatous polyposis coli (APC). Like NAV3, APC binds with tubulin and with actin and it undergoes dimerization (Wen et al., 2004). Likewise, both proteins enhance stability of microtubules through binding to plus tip proteins, such as EB1. These interactions might enable APC to regulate cell migration and establish epithelial polarity. Notably, many MT +Tips are regulated by phosphorylation at basic serine-rich regions, which reduces their affinity for MTs (Akhmanova and Steinmetz, 2008). This important regulation locally controls microtubule-stabilizing activity in different cellular compartments, such as the leading edge and neuronal growth cones (Akhmanova et al., 2001). For example, microtubule binding by APC is inhibited by glycogen synthase kinase 3 beta (GSK-3beta), which phosphorylates APC (Kumar et al., 2009) Apparently, GSK-3beta phosphorylation takes place at serine-rich regions, in the vicinity of SxIP motifs. Thus this phosphorylation negatively regulates localization of +Tips like APC to MT ends by decreasing their affinity to EB1 (Honnappa et al., 2009). We noted that NAV3 shares with APC the existence of SxIP motifs, which are embedded in serine-rich regions. On the basis of these observations we hypothesized that NAV3 binding to EB1 and MTs might also be regulated by phosphorylation, which is likely mediated by GSK-3beta. To establish whether a phosphorylation event links NAV3 and GSK-3beta we utilized a construct of RFP-tagged constitutively active (S9A)GSK-3beta, which was co-expressed with a FLAG-tagged NAV3. As expected, we observed a clear upward shift in NAV3's apparent molecular weight whenever FLAG-NAV3 was co-expressed with RFP-(S9A)GSK-3beta indicating that NAV3 might serve as a phosphorylation substrate of GSK-3beta. Moreover, increasing expression levels of GSK-3beta was accompanied by a reduction in NAV3 protein levels, suggesting that NAV3 undergoes degradation following phosphorylation (Fig.II-A). To further evaluate if GSK-3beta phosphorylation of NAV3 affects its binding to MT, we co-expressed EGFP-NAV3 and RFP-(S9A)GSK-3beta in COS-7 cells and used live cell imaging to detect NAV3`s MT plus-ends tracking activity. Overexpression of both NAV3 and active GSK-3beta resulted in partial to complete loss of NAV3 plus ends tracking activity and its translocation to the cytoplasm, whereas in cells that expressed no detectable RFP- GSK-3beta after transfection, NAV3 retained its localization to the MT plus-ends (Fig. II-B). In this vein, a MT sedimentation assay confirmed that co-expression of an active GSK-3beta with NAV3 led to diminution of the ability of NAV3 to co-sediment with MTs (Fig. II-C).

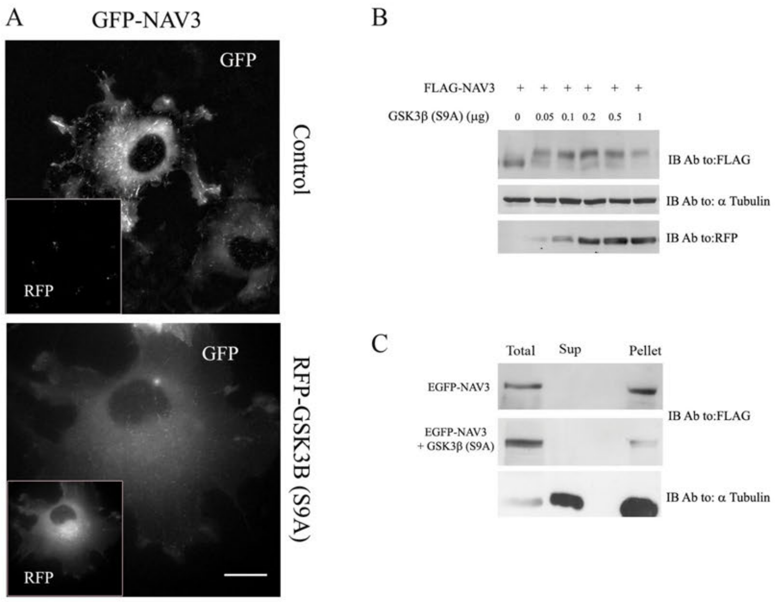

Figure II: ***GSK3b regulates NAV3 uncoupling from microtubules.***

(**A**) Live-cell images of COS-7 cells expressing EGFP-NAV3 without or with co-expression of RFP-tagged, constitutively active (S9A) GSK3beta (insets). Bar, 10 mm.

(**B**) HEK293 cells were co-transfected with a FLAG-tagged NAV3 vector and increasing concentrations of RFP-(S9A)GSK3beta. Whole cell extracts were prepared and subjected to immunoblotting with anti-FLAG, anti-tubulin and anti-RFP antibodies.

(C) Whole extracts from HEK293 cells previously transfected with EGFP-NAV3, either alone or with RFP-(S9A)GSK3beta, were subjected to a microtubule sedimentation assay.

Referee #1 (Remarks):

Specific Comments:

1) Silencing Nav3 reduces an EGF-stimulated increase in directional persistence, but how?

One key finding of our study, and in fact the starting evidence that motivated the study (Figure 1), has been the ability of EGF to transcriptionally up-regulate expression of NAV3. This has been demonstrated using DNA-arrays, as well as PCR and western blots. Concurrently, EGF enhances the directionally persistent mode of cell migration, but this does not occur if NAV3 is depleted. We attribute NAV3-dependent effects on mammary cell migration to the ability of both existing and newly synthesized navigator molecules to nucleate protein complexes that simultaneously regulate two cytoskeletal systems, the microtubule and the actin networks.

To further explore this scenario, we present below (Fig. III) experimental evidence supporting early, apparently transcription-independent effects of EGF on NAV3. The major findings might be summarized as follows:

(i) In addition to transcriptional up-regulation of NAV3, which occurs approximately three hours after EGF stimulation, an apparent stabilization of NAV3 molecules (or translocation to a soluble fraction) takes place as early as a few minutes after stimulation with EGF. This refers to both the endogenous form of NAV3 (Fig. III-A) and to the ectopically expressed GFP-NAV3 fusion protein (Fig. III-C), suggesting protein-level effects rather than promoter involvement.

(ii) In parallel to apparent stabilization, NAV3 molecules undergo tyrosine phosphorylation in response to EGF, and this is inhibited by an EGFR-specific kinase inhibitor (AG1478; Figs. III-B and III-C).

(iii) Assuming that the tyrosine-phosphorylated form of NAV3 nucleates a complex of proteins essential for its migration-modifying function, we applied a covalent crosslinking reagent and mass-spectrometry. These analyses identified several

potential partners of NAV3 (Fig. III-E), including NAV2, with which NAV3 might form heterodimers, and four different members of the 14-3-3 family of adaptors. Physical interactions between NAV3 and 14-3-3 was confirmed in experiments that used an antibody reactive with all 14-3-3 proteins (called 14-3-3 Pan), as well as another antibody, which recognizes a structural motif shared by all 14-3-3 binders (called 14-3-3 BM; Fig. III-F). Notably, in support of the MS results we observed homodimer formation of NAV3 molecules (data not shown) and a previous report showed that NAV2 interacts with 14-3-3 epsilon (Marzinke et al., 2013), whereas another publication reported that EGFR interacts with 14-3-3 zeta (Oksvold et al., 2004). Altogether, these lines of evidence raise the possibility that EGFR, 14-3-3 and NAV3 might form a ternary complex, a model we currently investigate.

(iv) Notably, several cytoskeletal proteins and microtubule binders, such as spectrin, dynein and MAP1B associated with NAV3 in the absence of a covalent crosslinking reagent, implying indirect or relatively weak binding.

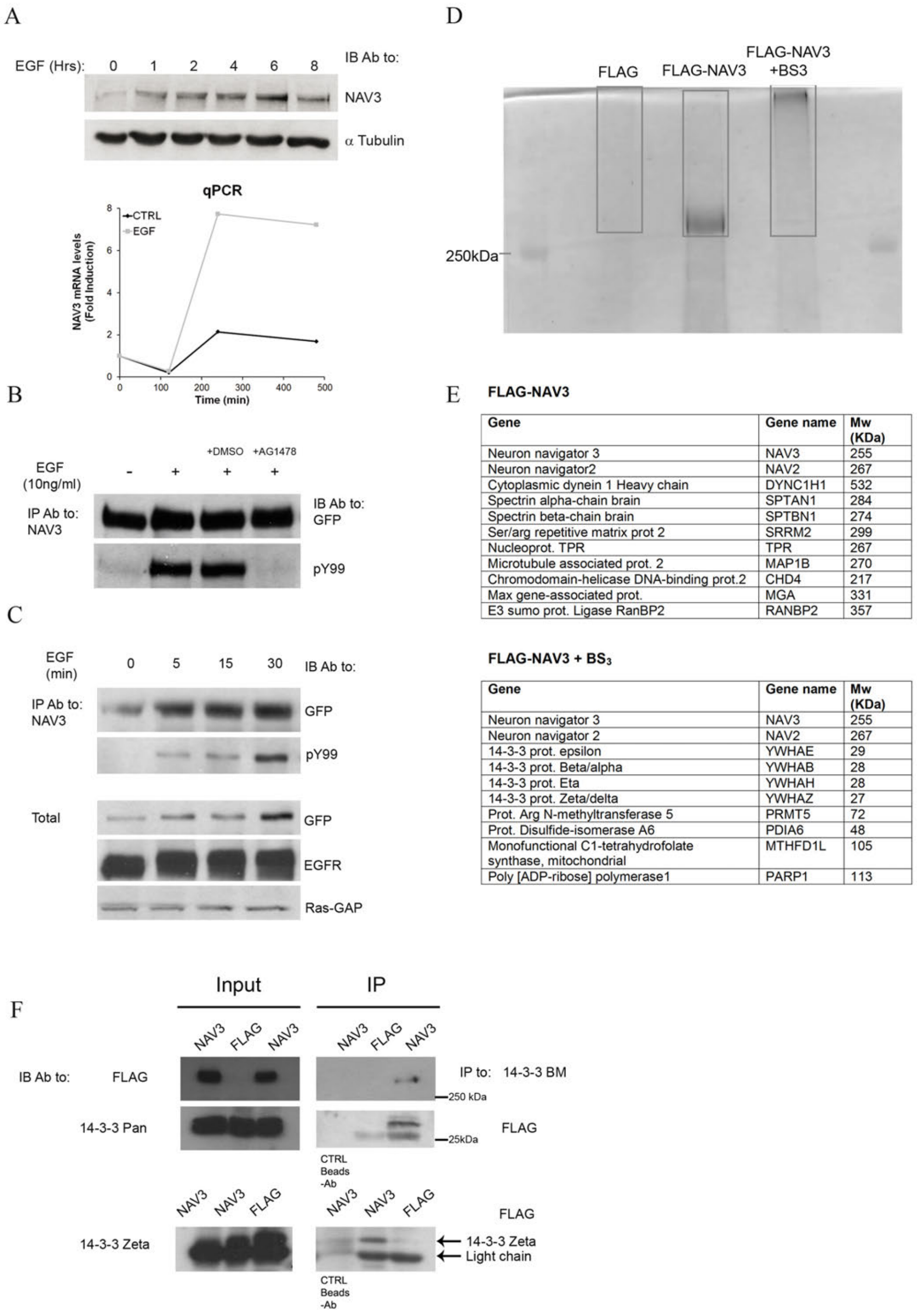

FLAG-NAV3

Gene	Gene name	Mw (KDa)
Neuron navigator 3	NAV3	255
Neuron navigator2	NAV2	267
Cytoplasmic dynein 1 Heavy chain	DYNC1H1	532
Spectrin alpha-chain brain	SPTAN1	284
Spectrin beta-chain brain	SPTBN1	274
Ser/arg repetitive matrix prot 2	SRRM2	299
Nucleoprot. TPR	TPR	267
Microtubule associated prot. 2	MAP1B	270
Chromodomain-helicase DNA-binding prot.2	CHD4	217
Max gene-associated prot.	MGA	331
E3 sumo prot. Ligase RanBP2	RANBP2	357

FLAG-NAV3 + BS_3

Gene	Gene name	Mw (KDa)
Neuron navigator 3	NAV3	255
Neuron navigator 2	NAV2	267
14-3-3 prot. epsilon	YWHAE	29
14-3-3 prot. Beta/alpha	YWHAB	28
14-3-3 prot. Eta	YWHAH	28
14-3-3 prot. Zeta/delta	YWHAZ	27
Prot. Arg N-methyltransferase 5	PRMT5	72
Prot. Disulfide-isomerase A6	PDIA6	48
Monofunctional C1-tetrahydrofolate synthase, mitochondrial	MTHFD1L	105
Poly [ADP-ribose] polymerase1	PARP1	113

Figure III: ***Evidence supporting early, transcription-independent effects of EGFR signals on NAV3.***

(**A**) Serum-starved MCF10A cells were treated with EGF (10 ng/ml) for the indicated intervals of time, and cell extracts were later prepared and subjected to immunoblotting (IB) with an antibody to NAV3 (upper panel). Similarly, RNA was extracted from EGF-treated MCF10A cells and changes in NAV3 expression levels were determined using quantitative PCR.

(**B**) HeLa cells stably expressing GFP-NAV3 were stimulated with EGF for 30 minutes, in the absence or presence of an EGFR-specific tyrosine kinase inhibitor. Cell extracts were subjected to immunoprecipitation (IP) and immunoblotting (IB) using anti-GFP and anti-phosphotyrosine (PY99) antibodies, as indicated.

(**C**) HeLa cells stably expressing GFP-NAV3 were stimulated with EGF for the indicated time intervals (5-30 minutes) and cell extracts were either directly subjected to immunoblotting (IB; lower panel), or they were first subjected to immunoprecipitation (IP) using an antibody to NAV3 and thereafter analysed using an antibody to phosphotyrosine (PY99).

(**D** and **E**) HEK293 cells were transfected with vectors encoding a FLAG-tagged NAV3 or FLAG alone. Forty-eight hours later, extracts from FLAG-NAV3 expressing cells were either untreated or they were subjected to protein covalent crosslinking using the bi-functional reagent BS^3. Finally, cell extracts were subjected to immunoprecipitation (IP) using anti-FLAG antibodies bound to beads. Rectangles mark the portions of gel processed for mass spectrometry (MS) analysis. The tables list proteins that physically interact with NAV3, in the absence or presence of the crosslinking reagent.

(**E**) HEK293 cells were transfected with plasmids encoding a FLAG-tagged NAV3 or FLAG only. Cell extracts were mixed with the indicated antibodies bound to beads. As control we used beads with no antibody bound to them. The immunoprecipitates were subjected to immunoblotting (IB) using a pan 14-3-3 antibody. Alternatively, we used an antibody to the binding motif shared by all 14-3-3 binders (14-3-3 BM).

In conclusion, NAV3 emerges from these new studies as a direct or indirect phosphorylation target of EGFR molecules. As a result, NAV3 undergoes apparent stabilization, as well as transcriptional induction, to nucleate a complex of proteins, including -3 family members, which might be involved in the mechanism leading to enhancement of directional cell migration. Because we plan to further study, in more details, the mechanisms underlying EGF- and NAV3-mediated effects on cell migration, the model and results presented in Figures I-III are only briefly mentioned in the revised manuscript.

Nav3 depletion also seems to increase sustained EGFR signalling (Fig 5C), so how does this work?

Employing a fluorescent derivative of EGF, cell sorter-based assays and immunoblotting, our observations found that of NAV3-depleted cells are characterized by defective endocytosis of EGFR and prolonged signalling to the AKT and ERK pathways (Figure 5 of the revised manuscript). We attribute the defect in internalization and inactivation of active EGFR molecules to the destabilized microtubule system of NAV3-depleted cells. This assumption is based on previous evidence linking EGFR endocytosis to intact microtubules. For example, paclitaxel (PTX), a chemotherapeutic drug, affects microtubule dynamics and also influences endocytic trafficking of EGFR (Li et al., 2012): Compared with the control cells, the velocity of directed motion of EGF-labelled quantum dots was reduced by 30% due to the suppression of high-speed movements along the microtubules in PTX-treated cells. Likewise, histone deacetylase 6 (HDAC6), a cytoplasmic lysine deacetylase, was found to negatively regulate EGFR endocytosis and degradation by controlling the acetylation status of alpha-tubulin and, subsequently, receptor trafficking along microtubules (Deribe et al., 2009; Gao et al., 2010). To better clarify this issue, we added an explanation and references to the respective part of the revised manuscript.

2) It also seems hard to explain how reduced chemotaxis to EGF of Nav3 depleted cells could lead to increased invasion and metastatic dissemination.

3) I didn't see the point of the mathematical model- what did it reveal that the data didn't already show? Perhaps this needs to be explained more clearly.

These two critical comments are connected and, as requested, we revised the relevant text to better clarify the issue. In essence, chemotaxis upward an EGF gradient requires directional persistence,

which depends on an intact NAV3. We agree that it is more intuitive to assume that defective chemotaxis might associate with lower, rather than higher potential to invade and metastasize, and this is exactly why we mathematically modelled migration patterns of NAV3-depleted cells.

What the modelling revealed is that the random, rather than the persistent mode of cell migration in 2D might better support metastasis in animal models. In other words, the mathematical model investigated relations between the type of cell migration and the probability of hitting rare targets (e.g., blood/lymph vessels or transient openings within the basement membrane). Essentially, cellular locomotion might be characterized by two parameters: speed and directionality. For example, the chemokine CXCL10 accelerates migration of blood cells without changing the non-random walk statistics (Harris et al., 2012), whereas our results demonstrated that EGF speeds up migration while enhancing directionality. The experimental data, when combined with the model, indicated that control cells treated with EGF displayed a directionally persistent walk, whereas under the same conditions NAV3-depleted cells displayed patterns expected from a random walk. This translates to the prediction that most persistent trajectories of cell migration would miss rare targets like openings within the basement membranes or nearby vessels, no matter how much time they are given. By contrast and counter-intuition, randomly migrating mammary cells, because they better explore their environment, will eventually hit rare targets in their vicinity, if given long enough time.

4) In the assays where the cells migrate through filters, how much of the effects seen could be due to cell proliferation/death changes?

All migration or invasion assays through filters have been conducted with a "loading" control, meaning that in parallel to cell seeding in the two-compartment chambers, cells of the same type and batch were also seeded in regular wells. This was followed by staining with crystal violet, at the end of the experiment, and normalization of the number of migrated cells to the loading control. By using this protocol we practically eliminated effects related to cell proliferation. Nevertheless, to directly address the critique, we inhibited cell proliferation by using mitomycin C and repeated the migration and invasion assays (Fig. IV). The results confirmed that migration and invasion assays of MCF10A cells were not significantly altered when proliferation was inhibited.

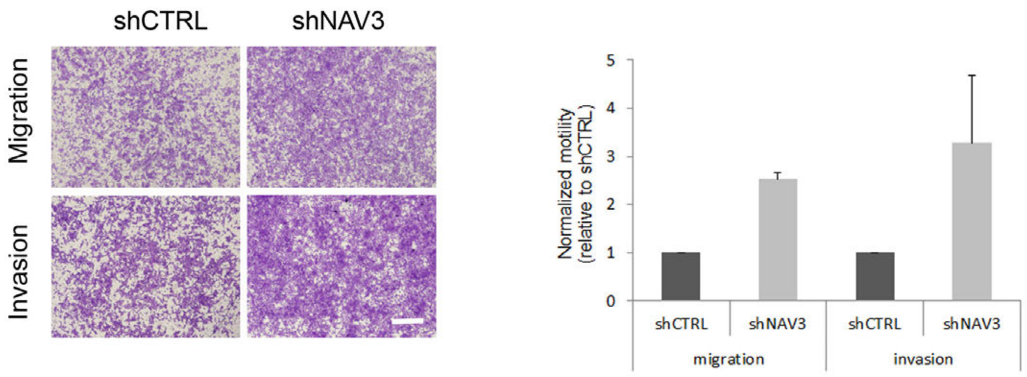

Figure IV: ***Cell proliferation may not enhance motility of NAV3-silenced cells.*** MCF10A cells stably expressing shNAV3 or shCTRL were treated for 2 hours with mitomycin C (0.2 mg/ml), followed by seeding in migration or invasion chambers in the presence of mitomycin D. Cells that reached the filter's bottom were stained, photographed and migration signals were quantified (bar, 100 mm). Shown are means ± S.D. values from three experiments.

The authors show that loss of Nav3 reduces apoptosis. How does this work? Is this effect only observed in 3D?

Luminal filling of 3D, acini-like structures, occurs when proliferation signals are combined with inhibition of apoptosis (reviewed in (Debnath and Brugge, 2005)). As requested, we assessed whether the reduced apoptosis observed in NAV3-depleted 3D cultures is reflected also in a 2D format. To this end we treated NAV3-silenced cells with cisplatin, a chemotherapy drug that triggers apoptosis. As shown in Figure V, the drug increased cleavage of caspase-3, a marker of apoptosis, only in control cells (shCTRL); silencing NAV3 using two different shRNAs abolished the apoptosis signal induced by both low and high concentrations of the chemotherapeutic drug. This result supports the pro-survival effect of NAV3 depletion, and it is consistent with the observed retarded apoptosis in 3D cultures, as well as with the observed increased of AKT activation of NAV3-depleted cells (Fig. 5 of the revised manuscript). The revised manuscript refers to the data shown in Figure V, but due to space limitations the actual results are not presented.

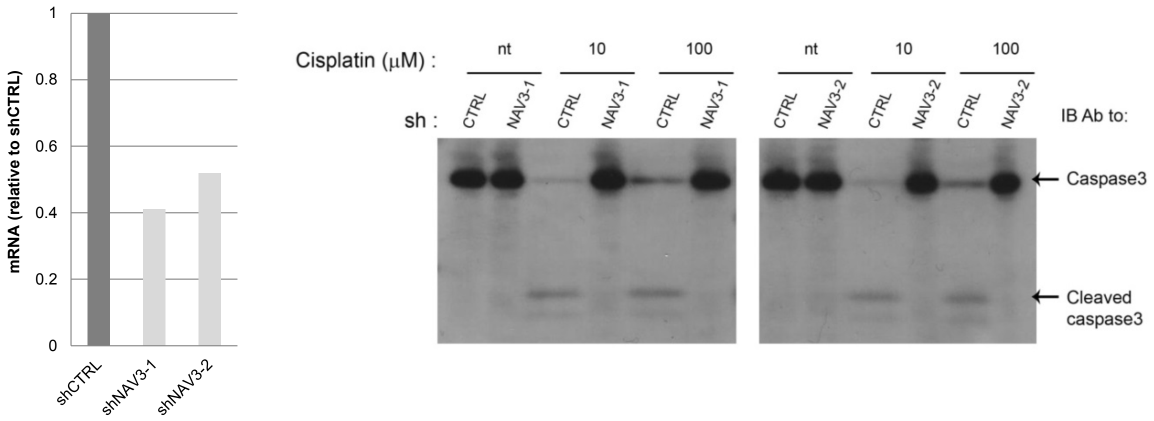

Figure V: ***Cisplatin treatment does not induce apoptosis of NAV3 silenced cells.*** MCF10A cells stably expressing either shControl or the indicated shNAV3 were tested for NAV3 expression using RT-PCR (left panel). In addition, the same cells were treated for 24 hours with the indicated concentrations of cisplatin (nt, no treatment). This was followed by western blot analysis of whole cell extracts with antibodies to caspase-3. Arrows mark the location of intact and cleaved casapse 3

5) Ectopic expression of Nav3 stabilizes microtubules and reduces catastrophe rate, but decreases the persistency of migration. This seems counterintuitive. Are these two observations connected in some way?

As indicated by the Referee, ectopic expression of NAV3 stabilizes microtubules and reduces catastrophe rate. However, ectopic NAV3 <u>increases</u> (not decreases) the directional persistence of cell migration, as shown in Figure VI.

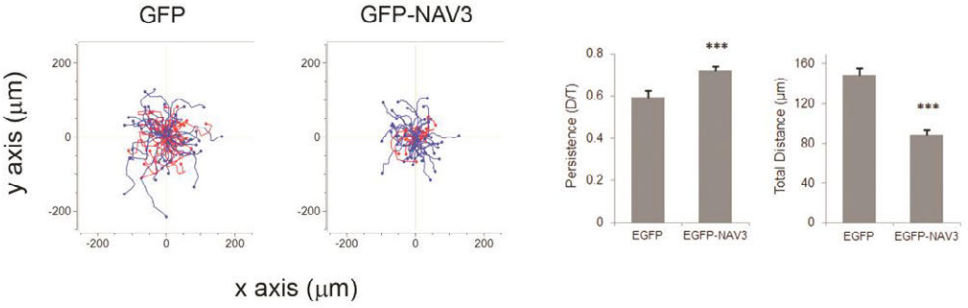

Figure VI: ***Ectopic expression of NAV3 increases directional persistence of mammary cells.***

MCF10A cells were transfected with plasmids encoding EGFP or EGFP-NAV3. Forty-eight hours later, while under EGF treatment, migrating cells were tracked and their trajectories were quantified for directional persistence and for total distance. Red coloured tracks indicate persistence values (D/T) smaller than 0.6. Results are depicted as means ±SEM values, based on 60 cells (data was pooled from three experiments). ****p*<0.0005; two-tailed student`s t-test (Note: this figure is identical to Figure S2D of the original manuscript).

5) In Figure 4D, the description given in the legend doesn't seem to match the figure.

As requested, we revised the legend to Figure 4D.

6) Why are the mutants of Nav3 defective? Do they express at normal levels in cells? Which function have they lost?

As requested, we examined stability of two point mutants of NAV3, A495P and D1047N, which were identified in human colorectal tumours. To determine relative rates of decay we blocked new protein synthesis and followed the decay of NAV3 protein. The results clearly indicated that the two mutants acquired significantly shorter rates of decay, as compared to the relatively stable, wild type form of NAV3 (Fig. VII and new Figure 6C of the revised manuscript). This observation raised the possibility that at least some mutations, in similarity to chromosomal deletions found in certain lymphomas (Karenko et al., 2005), lead to reduced expression levels of NAV3

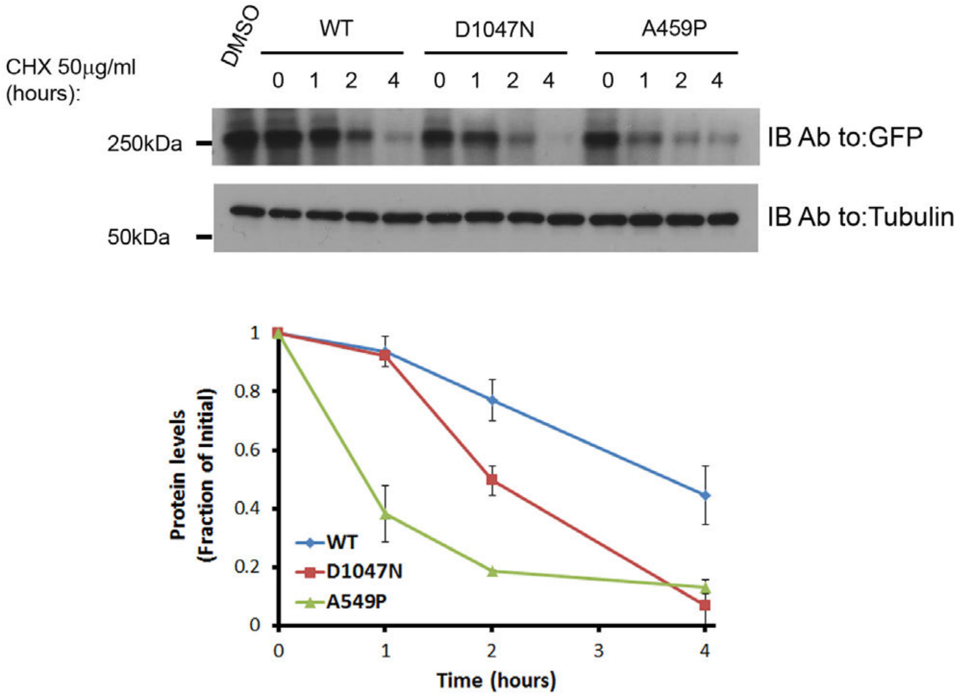

Figure VII: ***Two oncogenic point mutants of NAV3 are characterized by relatively short rates of decay.***

HEK293 cells previously transfected with EGFP-NAV3 (WT), EGFP-NAV3-D1047N or EGFP-NAV3-A459P were treated with cycloheximide (CHX; 50 mg/ml) for the indicated time intervals, and this was followed by cell lysis and immunoblotting using anti-EGFP and anti-alpha-tubulin antibodies. The signals obtained were quantified and normalized to time zero (untreated cells; bottom panel).

Referee #2 (Remarks)

The manuscript submitted by Cohen-Dvashi et al. provides an in-depth analysis of the potential role of navigator-3 (NAV3) as a promoter of directional migration in vitro. The knock-down of NAV-3 in MCF10-A alters the formation of a lumen in a 3D environment. Restoration of the expression NAV-3 in the aggressive MDA-MB-231 mammary adenocarcinoma cell line abrogates invasion in a 3D extracellular matrix and in in vivo metastatic colonization of the lung. The authors have explored the function of NAV-3 in vitro. Interestingly, NAV-3 localizes with End Binding Proteins at the plus end of dynamic microtubules and contributes to their stabilization through tubulin acetylation. This finding is related to the observation that EGFR signalling is short-lived as compared to its long half-life in NAV-3-depleted cells. A sustained activation of EGFR signalling is then hypothesized to be responsible for the lack of a chemotactic behaviour promoting random migration, especially in tumours with activated PI3K.

The authors found that a decreased expression of NAV-3 correlates with poorer survival in breast cancers, and this was associated with some of the 10 breast cancer molecular subtypes identified by Curtis et al.

Comments:
1) It is somewhat counter-intuitive that random migration observed in vitro would necessarily be more effective in the invasion of 3D environments.

We agree that it is counter-intuitive that random in vitro migration associates with stronger invasiveness in two animal models of metastasis. In fact, this perplexing finding has been the reason for our mathematical modelling of the effect of NAV3 on the mode of cell migration. The following potential mechanisms might explain the association between the random mode of cell migration and increased invasiveness in vitro and in animal models:

(i) As proposed by the mathematical model, the random mode of cell migration might confer a probabilistic advantage in terms of locating weaknesses in tissue barriers or hitting blood or lymph vessels.

(ii) NAV3-silencing might increased formation of proteolytic protrusions, thereby contribute to invasion in 3D environments.

(iii) The enhanced invasiveness of MDA-MB-231 cells depleted of NAV3 might be due to the sustained pattern of EGFR signaling characteristic of these cells. In line with this possibility, depletion of EGFR using siRNAs specific to EGFR reduced both invasion and migration of mammary tumor cells (Fig. VIII).

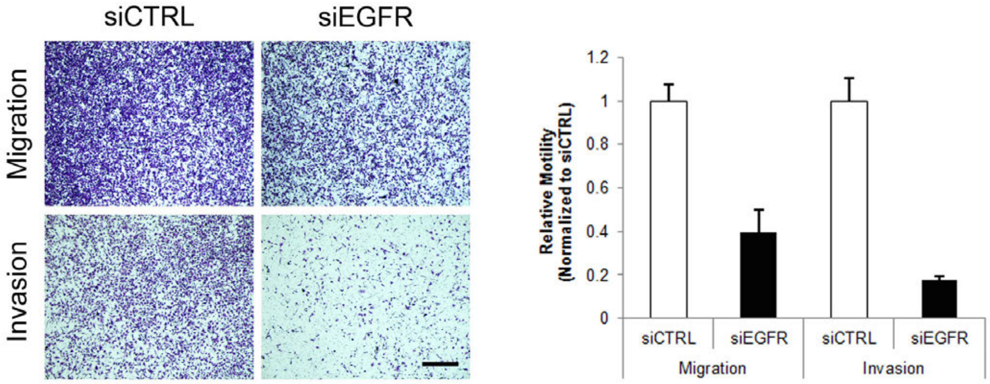

Figure VIII: ***Silencing EGFR reduces invasion of cultured mammary cells.***

MDA-MB-231 cells were transfected with control siRNA oligonucleotides or with EGFR-specific siRNA oligonucleotides. Cell migration and invasion assays were performed 48 hours later and the results were quantified (bar, 100 mm). Shown are means ± S.D. values of three experiments.

2) The authors should provide a more precise description of NAV-3 mutational status in tumours. The authors mention that is it rarely mutated (page 11).

As requested, the revised manuscript contains a new figure showing all known mutations of NAV3 (Figure IX, below, and Figure S5A of the revised manuscript). In addition, the revised text provides the following, more precise description of all mutations found in the *NAV3* gene: "In a study attempting to map the most frequently mutated genes in colorectal and in breast cancers (Wood et al., 2007), NAV3 was found to be highly mutated in colorectal cancers (CRC). Five somatic mutations (A495P, Q607H, D1047N, G2097V and S2341I), which may not be considered as SNPs, frequently appeared in the genomes of CRC specimens, designating *NAV3* as one of the 60 most frequently mutated genes in CRC. These mutations received a low score for being 'passenger' aberrations, hence they might qualify as 'driver' mutations. Another mutation (D220H), which was found in breast cancer, received a high 'passenger' probability score. A nonsense mutation (Q200*) was found only in melanomas (Bleeker et al., 2009), and four frequent missense mutations were found in renal cell carcinoma (Guo et al., 2012)".

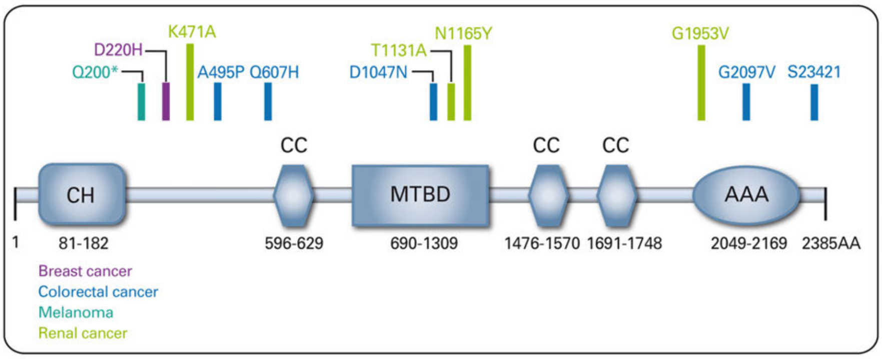

Figure VIX: ***A scheme showing the locations of all mutations within the coding regions of the human NAV3 gene.***

Note the colour code of each mutation, according to the tissue of origin. Numbers indicate amino acids. Shown are the calponin homology (CH) domain, the coiled coil (CC) regions, the microtubule-binding domain (MTBD) and the triple A domain, which functions as an ATPase.

3) The authors should comment on the fact that low NAV-3 expression correlates with poor survival only in ER-positive tumours. EGFR receptor signalling seems more clearly associated with triple-negative tumours; these tumours could also express NAV-3, as it is a target of EFGR signalling.

As requested, we modified the relevant paragraph, such that the new text refers to the association between low NAV3 and poor prognosis of ER-positive breast cancer. Essentially, we assume that NAV3 alterations take place at an early step of breast cancer progression, when tumours are still hormone-dependent and the number of genetic alterations is relatively small. At later steps, more penetrant genetic alterations might preempt the initial driver roles of NAV3 loss or aberrant forms of the gene. This might also refer to the triple negative molecular subtype, which often presents genetic defects in strong tumour-suppressors, like p53 and BRCA1, along with activation of oncogenes like PI3K and EGFR. Our on-going experiments test promoter reporter plasmids of NAV3 for possible responses to steroids, such as estrogen, progesterone and glucocorticoids.

4) Many genes have been found to correlate with survival but most of them have not been shown to impact tumour progression outside a few cell line models. The authors may consider analysing classical breast tumours models with a wild type or deleted NAV-3 background.

Following this comment, we initiated studies using a well-characterized murine model of breast cancer, namely 4T1. This rather aggressive tumour is being modified now with lentiviruses that specifically deplete the endogenous form of *Nav3*. Within a few months we will introduce stably

depleted tumour cells into immunocompetent animals and test the effect of *Nav3* in a syngeneic model of metastasis.

Referee #3 (Remarks):

The manuscript by Cohen-Dvashi and collaborators describes that the microtubule + end binding protein NAV3, induced by EGF in the MCF10A breast cancer cell line, inhibits EGF-induced cell velocity, but enhances persistent migration, and invasion and experimental metastasis by the MDA-MB231 cell line. The author claims that these effects might be the consequence of increased microtubule growth. NAV3 mutants similar to the ones found in cancer are functionally impaired for directional persistence and metastasis formation. As NAV3 is expressed at low levels in breast tumours, and is associated with low survival, the data suggests that NAV3 might be a suppressor of breast cancer progression.

While the study provides lots of interesting evidence linking NAV3 to microtubule stability and cell invasion, it falls short on the molecular mechanisms. NAV proteins are known +TIPs and, as such, were previously associated to microtubule stability and directional migration (of neurons). This study confirms that ectopically expressed NAV3 is a +TIP and shows that it has an impact on microtubule dynamics, favouring microtubule growth. It also shows that NAV3 depletion leads to increased formation of invadopodia, even though no data is provided that support a causal link between decreased microtubule stability and increased number of invadopodia. There is also no demonstrated connection between NAV3-induced microtubule stability and reduced metastasis.

Because NAV3 knockdown also induced increased growth of primary tumours, it is not obvious whether the impact on metastasis is due to the effects on tumour growth or on invasion. Very interestingly two NAV3 mutants, that recapitulate mutations found in tumours, are functionally inactive. However, how, at the molecular level, these mutations affects NAV3 activity is not explored.

Thus despite the interest of the data, the study does not provide significant insights into the mechanism of action underlying NAV3 function as a potential suppressor of cancer progression.

What follows is a list of responses and new data that answer in part the general comments of this Referee. We later detail our responses to the eight specific comments listed by this Referee.

(i) The mechanism of action underlying NAV3 function as a potential suppressor of cancer progression

This Referee focused his/her critique on the mechanism of action of NAV3. And although the Editor commented to us that "Extensive mechanistic insight is not an absolute requirement for *EMBO Molecular Medicine* when the clinical implications and translational relevance are striking", we adhered to the critique and performed several new experiments that aimed at the function of NAV3 in cancer progression.

Figure I presents one working model of our mechanism-based studies and potential relations to signal transduction pathways. The model assigns pivotal roles for Rac1, a Rho family member, because a wealth of in vitro studies revealed that Rac1 controls switching between the directionally persistent and the random modes of cell migration. For example, a study by Ken Yamada and colleagues described a Rac mechanism for determining whether cell patterns of migration are intrinsically random or directionally persistent (Pankov et al., 2005). Most important, Rac activity promoted formation of peripheral lamellae, which mediated random migration. Accordingly, decreasing Rac activity suppressed peripheral lamellae and switched cell migration patterns of fibroblasts and epithelial cells from random to directionally persistent. Accordingly, the number, extent, and direction of lamellipodia correlate well with the speed and direction of cell migration (Petrie et al., 2009). Lamellipodial protrusions are controlled by actin polymerization and the WAVE/Arp2/3 complex, a target of Rac1. Total Rac1 activity can therefore provide a regulatory switch between the two major patterns of cell migration. For example, Slit-Robo GAP 1 (srGAP1) is a modulator of Rac activity in locomotive cells (Yamazaki et al., 2013). srGAP1 possesses a GAP activity specific to Rac1 and is recruited to lamellipodia in a Rac1-dependent manner. Depletion of srGAP1 over activates Rac1 and inactivates RhoA, resulting in continuous spatiotemporal spreading

of lamellipodia and a modal shift. To test relevance of Rac1 to EGF-induced migration of mammary cells, we combined EGF treatment with a specific inhibitor of Rac1. The results presented in Figure X confirm that Rac1 activity is necessary for robust migration of MCF10A cells in response to EGF. Furthermore, in agreement with Yamada's and other studies, directional persistence significantly increased, implying that high Rac1 activity dictates intrinsically random migration in our model of mammary cells.

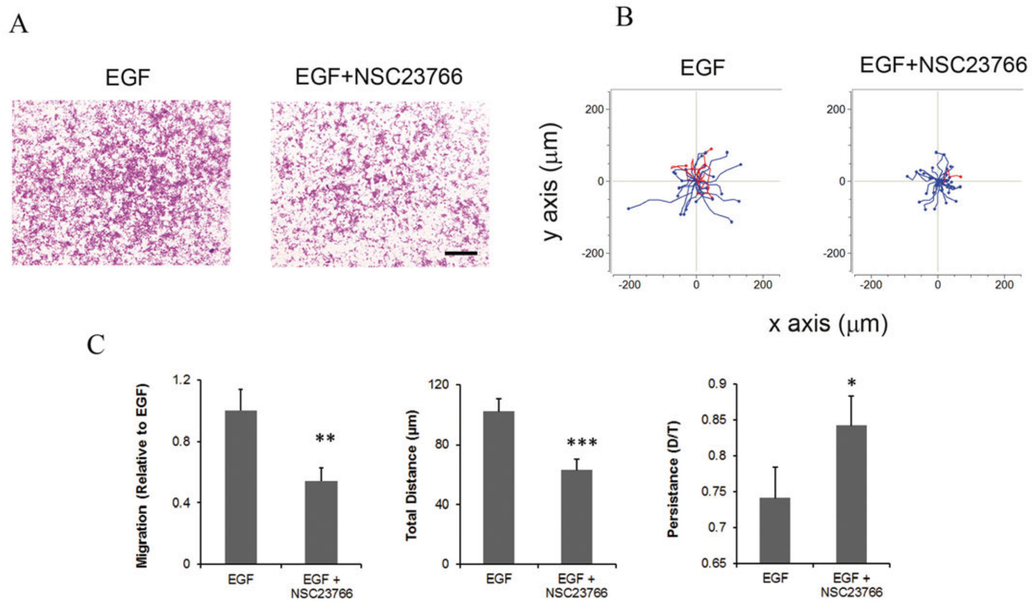

Figure X: ***Rac1 might control directional persistence of EGF-induced migration of mammary cells.***

(A) Migration of MCF10A cells was assessed in the presence of EGF, either alone or in combination with a Rac1 inhibitor (NSC23766; 10 μM). Cells were cultured for 18 hours in migration chambers (6×10^4 cells/well) and cells that migrated through the intervening filter were stained and photographed (bar, 100 mm).

(B) Migration tracks of MCF10A cells that were treated as in A were recorded.

(C) The histograms present quantification of the tracks shown in B in terms of migration, distance (relative velocity) and directional persistence.

(ii) A causal link between decreased microtubule stability and enhanced invasiveness

Although a link between MT stability and metastasis was not thoroughly investigated by us, we note that it has previously been shown that reducing MT stability (by Nocodazole) enhances ingression through the basement membrane in an EMT model, while inducing MT stability (using Taxol) reduced invasiveness and increased RhoA activation (Nakaya et al., 2008). To assess the impact of MT stability on invasiveness in our cellular model, we conducted additional invadopodia assays, in which the cells were pre-incubated with either Nocodazole or Taxol, prior to seeding on FITC-gelatin matrix. As demonstrated in Figure XI, Nocodazole moderately enhanced invadopodia formation, whereas Taxol significantly reduced the number of invadopodia, in line with a role for microtubule destabilization in invadopodia formation and matrix invasion.

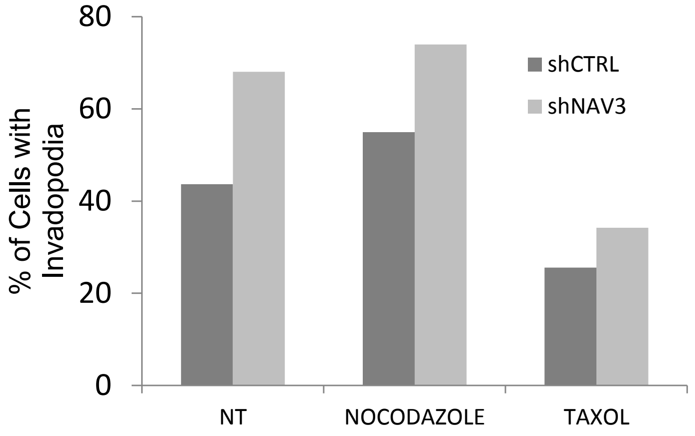

Figure XI: ***Microtubule stability regulates invadopodia formation.***

MDA-MB-231 cells, stably expressing control or NAV3-targeted shRNAs were treated with Nocodazole or Taxol (each at 10 mM) for 30 minutes prior to plating on coverslips pre-coated with a fluorescent gelatin. Note that we supplemented the medium with either Nocodazole or Taxol, as appropriate. Two hours later, cells were fixed and probed for active invadopodia using actin and TKS5 as markers. The percentages of cells with invadopodia were quantified (~100 cells analysed per condition).

(iii) How individual mutations affects NAV3 activity

In response to a similar question, raised by another Referee, we tested the stability of mutant NAV3 proteins and found that they are unstable relative to the wild type form (see Fig. VII). Hence, it is conceivable that the mutant forms of NAV3 phenocopy depletion of the wild type protein.

(iv) Relations between increased growth of NAV3-depleted primary tumors and their enhanced metastasis

We agree that the dual in vivo effect of NAV3 depletion (i.e., increased tumour volume and enhanced metastasis) compounds interpretation of data from experiments in which genetically manipulated MDA-MB-231 cells were injected into the mammary fat pad and gave rise to relatively large primary tumours. However, to overcome this we used a *second* animal model that is not affected by the size of the primary tumour. This other type of analysis directly introduced tumour cells into the circulation of immunocompromised mice. Because the same numbers of tumour cells were used for tail vein injection, and shNAV3 cells seeded more metastases in lungs, we concluded that NAV3 also regulates cellular invasion (extravasation) and metastasis.

Other comments:

1. Transient transfection of siRNA is used throughout the manuscript. The depletion of NAV3 should be shown for every experiment, if possible at the protein level. Similarly the level of NAV3 upon shRNA-mediated depletion must be verified, especially in long-term experiments (3D growth, metastasis). This is especially true because cells are sometimes challenged at the same time with EGF which is supposed to increase NAV3 expression and siRNA which will bring down the expression levels. Moreover, it would be nice to validate, at least some of the key results, with another siRNA/shRNA.

In all of our transient siRNA transfections and knockdown experiments we used a mixture of four NAV3-specific oligonucleotides (siGenome On-Target, from Dharmacon), as described under Methods. According to the manufacturer, using the mixture reduces off-target effects. As control we used scrambled On-Target Oligonucleotides (four different oligonucleotides, corresponding to no known mRNA) from the same manufacturer. To specifically address the comment, we examined several individual oligonucleotides against NAV3. As shown in Figure XII, two oligonucleotides yielded knockdown efficiencies similar to those achieved by the mixture of oligonucleotides, and

also yielded similar inhibition of cell migration in the Transwell assay. The revised text refers to the use of RNA interference and also presents the results in Figure S2C.

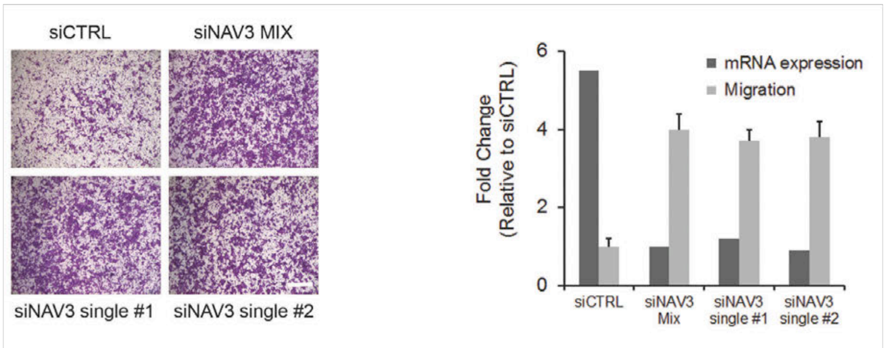

Figure XII: ***siRNA oligonucleotides targeting NAV3 reduce expression of NAV3 and enhance migration of human mammary cells.***

MCF10A cells were transfected with the following siRNA oligonucleotides: control oligonucleotides, two oligonucleotides targeting non-overlapping regions of the NAV3 transcript, as well as a mixture of four oligonucleotides (number 1 and number 2, and two additional oligonucleotides). Forty-eight hours following transfection, cells were transferred into migration chambers and cultured for additional 18 hours. Cells that reached the filter's bottom were stained and the respective area coverage was quantified (bar, 100 mm). Alternatively, transfected cells were lysed and total RNA was subjected to quantitative RT-PCR using NAV3-specific primers. Relative cell migration (mean ± S.D. of three experiments) and relative NAV3 mRNA levels are depicted as fold-change (normalized to siCTRL).

According to our experience, mixtures of siRNAs yield relatively high knockdown efficiencies in short-term assays. For long-term assays, such as 3D cultures and animal studies, we used specific shRNAs. All along the study we routinely verified knockdown efficiencies at the mRNA or protein levels using RT-PCR or immunoblotting. A representative experiment using both PCR and immunoblotting is shown below, as Figure XIII, and in the revised manuscript, as Fig. 4D.

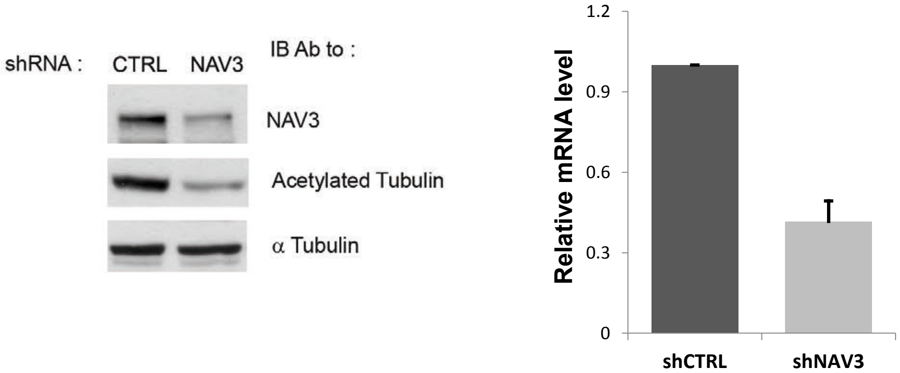

Figure XIII: ***Confirmation of the ability of short hairpins to down regulate NAV3 expression at the levels of both mRNA and protein.*** The indicated stable derivatives of MDA-MB-231 cells were lysed and extracts were subjected to immunoblotting with anti-NAV3, anti-acetylated tubulin or anti-alpha tubulin antibodies (left panel). RT-PCR measurements (from three different experiments) of knockdown efficiency are shown in the right panel.

2. What is the relative expression level of NAV3 in the various cellular models used in the study: MCF10A, MDA-MB231, HEK293 and COS7? Are MCF10A representative of normal cells with high expression levels? Are MDA-MB231 representative of aggressive breast cancer cells with low NAV3 (which would question the rationale of further down-regulating NAV3 expression)?

Are there breast cancer cells lines devoid of NAV3 expression (or expressing NAV3 mutants)? If yes, is there a correlation between expression of NAV3 and migration/invasion efficiency?

As requested, we subjected the four cell lines indicated by the Referee to immunoblotting with an anti-NAV3 antibody. The results we obtained are presented in Figure XIV. Along with a confirmation of EGF-induced up-regulation of NAV3, we observed additional alterations in NAV3 or related proteins, which might represent products of alternatively spliced mRNAs. These alterations are currently being investigated. So far, using a small set of cell lines, we have been unable to correlate NAV3 abundance with other cellular attributes, probably because breast cancer cell lines often represent advanced stages of the disease.

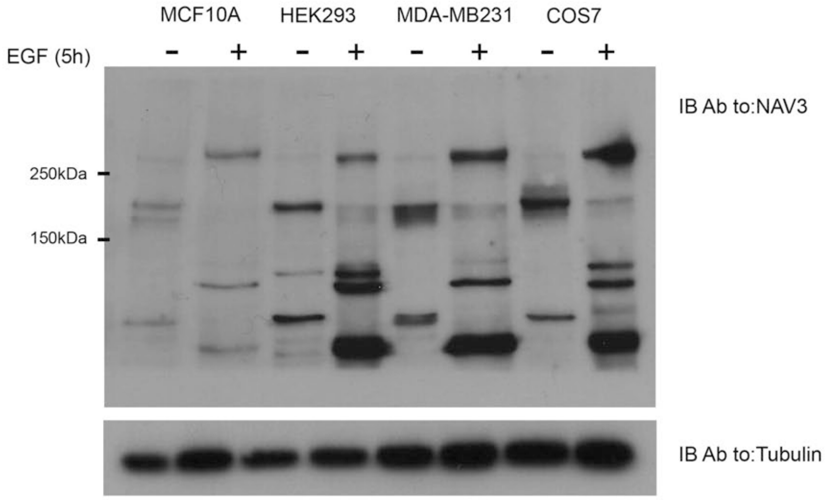

Figure XIV: ***EGF stimulation augments NAV3 levels in different cell lines.*** MCF10A, HEK293, MDA-MB-231 and COS7 cells were equally seeded. Following attachment to the substrate, cells were starved for serum factors for 18 hours and later stimulated with EGF (10 ng/ml) for 5 hours. Thereafter, the cells were lysed and whole cell extracts were subjected to immunoblotting using an anti-NAV3 antibody. Note that the uppermost protein band represents NAV3.

3. Is NAV3 also induced by EGF in other cell lines, e.g. the MDA-MB231? And why would pro-oncogenic EGF signalling lead to expression of a suppressor of invasion? How does this relate to aggressive breast tumours which express EGFR, but have low NAV3? Inversely is EGFR also induced by shNAV3 in cell lines others than the MDA-MB231. NAV3 appears to be down regulated in a large majority of breast tumours, irrespective of their EGFR status. From the discussion, it is not clear what the model is and whether the authors propose that the effect of NAV3 depletion is necessarily mediated via EGF signalling or not.

Actually, most experiments using MDA-MB231 cells are performed in the absence of EGF (from my understanding of the legends and methods): cell invasion, invadopodia formation, cell proliferation, and microtubule dynamics (except for MDA-MB231 cell persistence and velocity which were unfortunately not tested in the absence of EGF). So it seems that most effects are independent of EGF signalling.

(i) As requested, we tested MDA-MB-231 cells for induction of NAV3 following EGF stimulation and found that these highly invasive cells up-regulate NAV3 expression quite rapidly after stimulation (see Fig. XIV).

(ii) We do not know why a pro-oncogenic EGF signaling lead to expression of a suppressor of invasion. However, this might relate to the developmental roles of EGF family ligands as drivers of morphogenesis and migration of progenitor cells. In addition, it is notable that NAV3 slows down the rate of cell migration while biasing the directionally persistent mode. These attributes might represent a delayed feedback loop of EGF-induced chemotaxis, which is nullified by malignant transformation of mammary cells.

(iii) Our immunohistochemical analyses of breast cancer specimens proposed an association between high EGFR and low abundance of NAV3 (see Figure 7 of the revised manuscript).

(iv) Our working model (and results presented in Figure 5 of the revised manuscript) assumes that lowering NAV3 abundance enhances stability of EGFR, thereby prolongs mitogenic and survival signaling, which are respectively generated by the ERK and AKT pathways.

(v) MDA-MB-231 cells carry mutant forms of both KRAS and p53, hence overexpress HB-EGF and TGF-alpha (Lehmann et al., 2000), along with amphiregulin (Sauer et al., 2010; Taira et al., 2014). As shown below (Fig. XV), both HB-EGF and TGF-alpha, like EGF, up-regulate expression of NAV3 and accelerate mammary cell migration. Because of these autocrine loops we refrained from testing the effects of exogenous EGF on MDA-MB-231 cells.

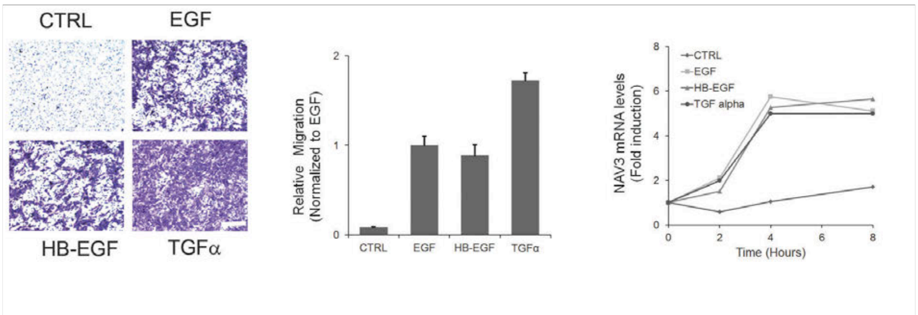

Figure XV: ***Both HB-EGF and TGF-alpha, like EGF, up-regulate NAV3 expression and enhance mammary cell migration.***

(Left and middle) MCF10A cells were cultured for 18 hours in migration chambers (6×10^4 cells/well) containing medium supplemented with the indicated growth factors (10 ng/ml) or with serum (5%; CTRL). Cells that reached the filter's bottom were stained and the respective area coverage was quantified (bar, 100 mm). Relative migration signals were normalized to EGF.

(Right) Serum starved MCF10A cells were stimulated with individual growth factors for the indicated time intervals. Thereafter, cells were lysed and total RNA was prepared and subjected to quantitative PCR using NAV3-specific primers. (Note: this figure is identical to a portion of Fig. S2 of the original manuscript).

4. In gain of function experiments, expression levels of NAV3 (Fig. S2) and NAV3 mutants (Fig. 6 and S4 and S5) relative to endogenous NAV3 should be shown. The rationale for testing only the D1047N and A495P mutants is not provided. Why are gain of function experiments not performed in MDA-MB231 (which again should have low expression of NAV3)?

As requested, we compared the levels of ectopic NAV3 (wild type and mutants) to the expression level of the endogenous form of NAV3 (see Figure XVI, below). Notably, the ectopic form was expressed approximately 5 times higher than the endogenous form (cell expressing GFP only). The revised text refers now to this observation.

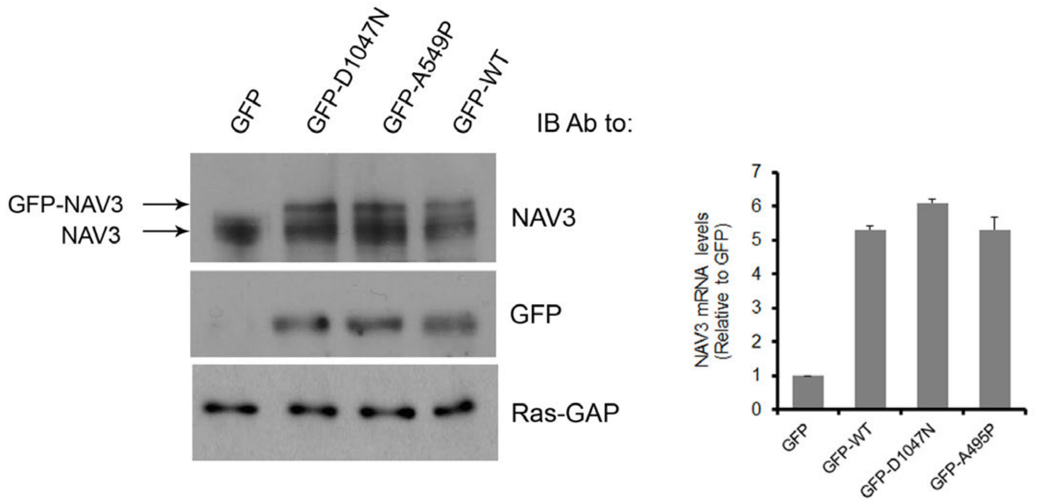

Figure XVI: ***Expression levels of GFP-NAV3 and mutants relative to the endogenous NAV3.***

MDA-MB-231 cells stably expressing the indicated GFP constructs were lysed, and extracts were subjected to immunoblotting with either anti-GFP or anti-NAV3 antibodies. In addition, RNA was extracted from the cells, and used for RT-PCR analysis with NAV3's specific primers.

Technical reasons have so far prevented us from more extensive studies of additional mutant forms of NAV3 in MDA-MB-231 cells. We did introduce all five CRC somatic mutations in a GFP-NAV3 fusion. However, because of the unusual size of NAV3's cDNA (>7 kilobase), selecting stable clones turned out to be quite challenging. So far we succeeded in generating stable clones for GFP, GFP-NAV3 and two mutations: D1047N and A495P. To circumvent these technical obstacles, we are currently generating stable isogenic MDA-MB-231/NAV3 cell lines by utilizing the Flp-In recombination system. A vector containing a Flp recombinase target site, as well as an antibiotic selection marker (hygromycin), was stably integrated into the MDA-MB-231 genome to generate a clonal MDA-MB-231/Flp isogenic acceptor line. The MDA-MB-231/Flp cell line was validated for single-copy integration of the FRT site by Southern blotting. For the generation of MDA-MB-231/Flp/NAV3 isogenic sublines, FRT expression vectors containing a tet repressor gene (tetR), as well as a tetracycline-responsive CMV promoter, were cloned upstream of the wild type and mutant NAV3 open reading frames for inducible expression. One of these ORFs encodes the wild type protein, the other five express different mutant forms of NAV3 (i.e., AP, SI, GV, DN, DH and QH). After sequence verification, the NAV3 constructs were individually co-transfected, together with a Flp recombinase expression vector (pOG44), to integrate the respective open reading frame in the targeted locus of the MDA-MB-231/Flp cell line by site specific recombination. Figure XVII presents our current progress and indicates successful integration into the targeted locus. Unfortunately, establishing stable clones of all mutants will involve several more months of investigation.

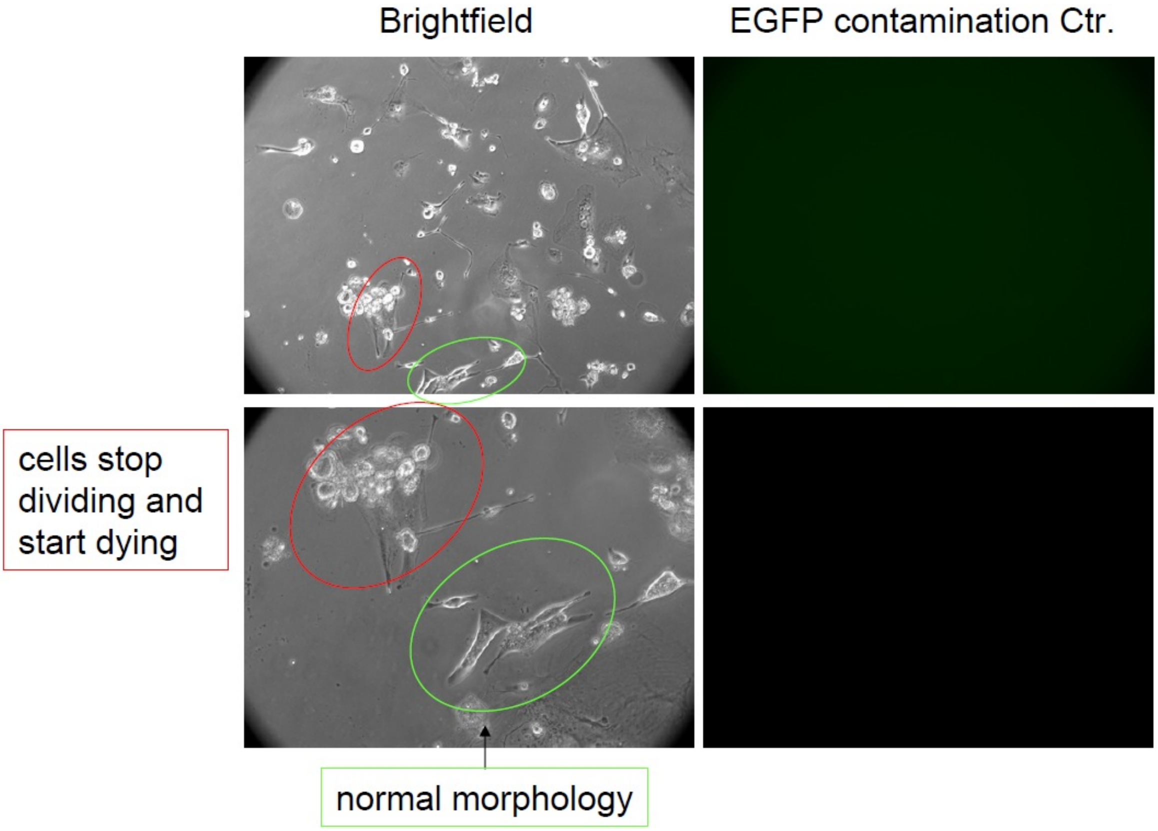

Figure XVII: Cell m*orphology of the DH_NAV3 mutant expressed in isogenic MDA-MB-231 cells.* Cells were transfected with the DH-mutant. Single colonies were picked. Shown are bright field images (left) and an EGFP control (right). The EGFP ORF is contained in the original acceptor line, which is replaced by the respective ORF by recombination and integration. Negative GFP signals indicate successful ORF integration, which is the DH-mutant.

5. If the authors state that MCF10A acini expressing shNAV3 are larger (Fig. 2), the data should be quantified. Moreover they also claim that the acini are amorphous. Is this related to an over proliferative or an invasive phenotype? The claim that the observed effect on apoptosis can support an involvement of NAV3 in polarity is surprising. No markers of polarity are used to support this claim. And surprisingly, lumen formation or cell polarization is not observed in control cells, which makes it difficult to evaluate the impact of NAV3 on polarity. The impact of NAV3 on cell proliferation should have been tested over longer period of time, in 2D and 3D models. Because NAV3 depletion has an effect on primary tumour growth and because it induces larger acini, it is likely to have an effect on proliferation and/or survival.

(i) As requested, we quantified the volume of the different acini and presented the results in Figure XVIII (new Figure 2C of the revised manuscript). As shown, the acini of NAV3-depleted cells were, on average, larger than the control acini.

(ii) To examine potential invasiveness of NAV3-depleted acini, we stained them with antibodies to either laminin or beta-catenin (Fig. XVIII, lower panel). The results indicated that both control and NAV3-depleted cells maintain integrity of the basement membrane that encapsulates the respective acini.

(iii) Following the critical comments of this Referee in relation to polarity, the revised text does not refer to effects on polarity.

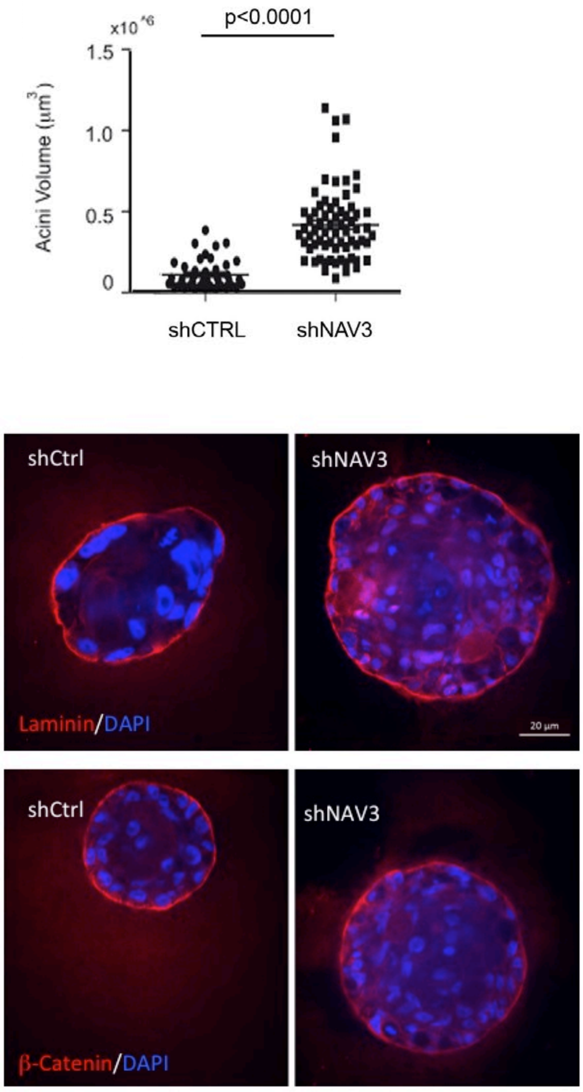

Figure XVIII: ***Acini formed by NAV3-depleted cells are larger than control acini but their basement remains intact.***

MACF10A cells stably expressing shCTRL or shNAV3 were grown in 3D matrix for 8 days. The acini formed were photographed (bar, 20 mm) and either quantified for their volumes using ImageJ (**upper panel**) or they were stained using antibodies to beta-catenin and laminin (**lower panel**). Note the integrity of the basement membrane of NAV3-depleted cells.

The impact of NAV3 on cell proliferation should have been tested over longer period of time, in 2D and 3D models. Because NAV3 depletion has an effect on primary tumour growth and because it induces larger acini, it is likely to have an effect on proliferation and/or survival.

As requested, we examined the effect of NAV3 depletion on long-term proliferation of human mammary cells. Comparison of cells infected with particles encoding two different shRNAs, as well as control shRNA, indicated that depleted cells might gain slightly more rapid rates of proliferation after 7 days in culture.

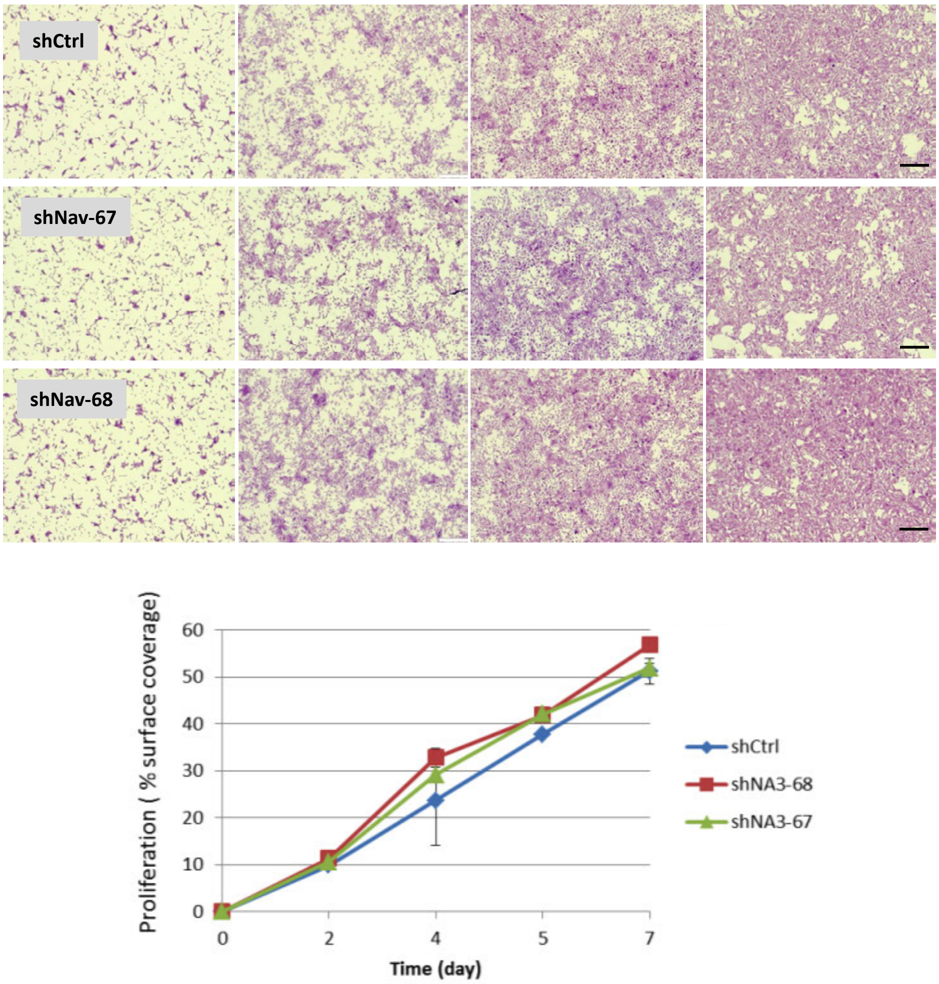

Figure XIX: ***NAV3-silencing only weakly affects long-term proliferation of MCF10A cells.***

MCF10A cells stably expressing shCTRL, or the indicated clone of shNAV3, were equally seeded. At the indicated days, cells were stained and photographed, and the percentage of area coverage was quantified using ImageJ (bar, 100 mm). Results are depicted as mean ± S.D. values of two biological repeats.

6. Is endogenous NAV3 capable of tip-tracking? Apparently not, from the Fig. 4C. This should raise questions relative to its mode of action, that deserved to be discussed. While depleting NAV3 decreases acetylated tubulin, does it modify the density or organization of the microtubule network?

Following this critical comment we replaced Figure 4C with a new figure, which is shown below (Fig. XX). The new figure documents cells that were differently fixed to better preserve microtubule structures. As shown, staining of endogenous NAV3 confirmed physical associations with microtubules. Notably, co-localization was reduced on treatment with Nocodazole and strengthened by Taxol. While NAV3 seemed to decorate the whole microtubule shaft, according to results presented in the lower panel of Fig. XX, under the same staining conditions, EB1 displayed similar fiber decoration.

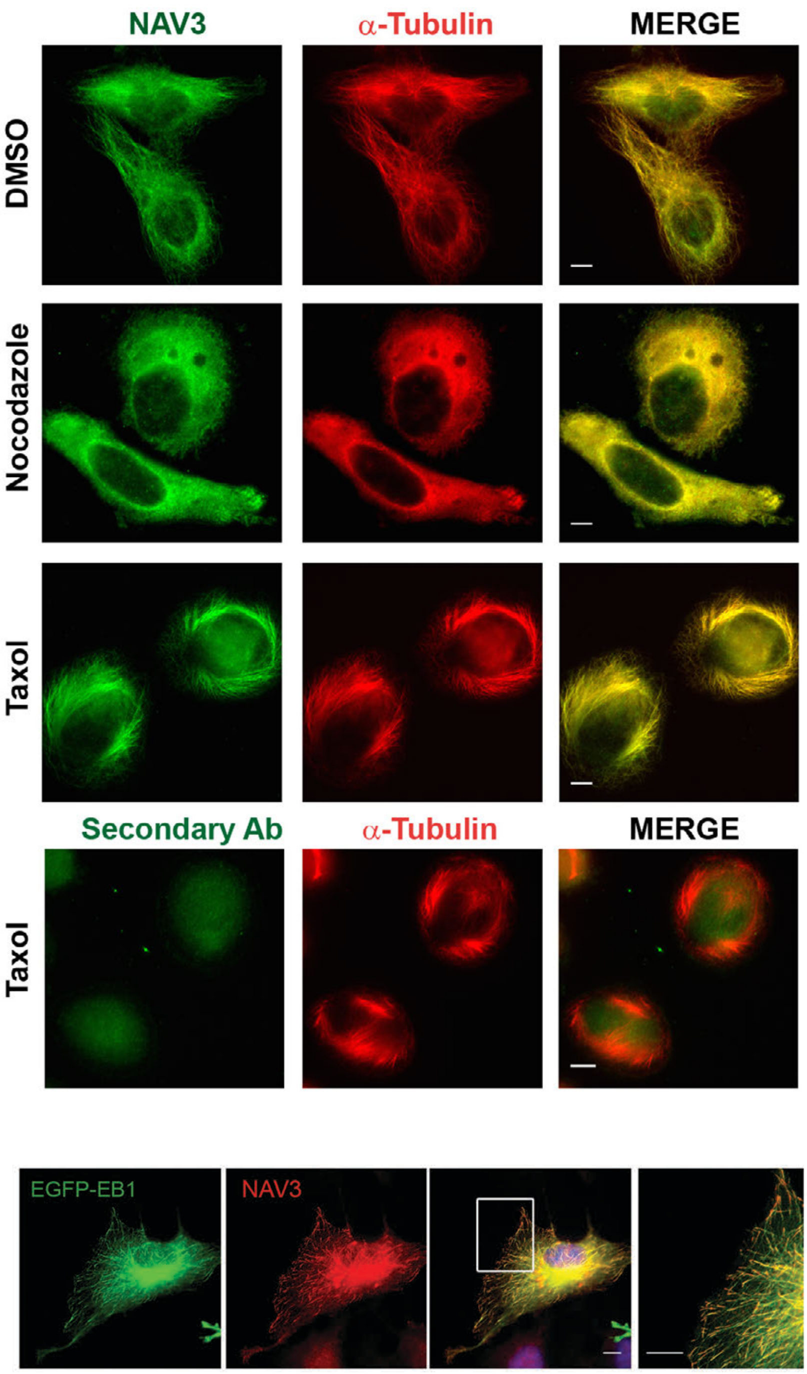

Figure XX: ***Endogenous NAV3 co localizes with microtubules.***

(**Upper**) MDA-MB-231 cells were treated for 4 hours with either dimethyl sulphoxide (DMSO), 10 μM Nocodazole or 10 μM Taxol, and subsequently double stained with anti-NAV3 (green, Cy2) and anti-α-tubulin antibodies (red, Cy5). Merged images (yellow) are also shown. As control, Taxol treated cells were stained with the secondary antibody only. All scale bars, 10 mm.

(**Lower**) Images of COS-7 cells co-expressing FLAG-NAV3 and GFP-EB1. Cells were immunostained with an anti-NAV3 antibody and with DAPI. The rectangle marks the enlarged area depicted in the far right panel (bars, 10 and 5 μm).

Does NAV3 overexpression induce MT bundles? Does it induce cell extension as described previously?

As indicated by this Referee, NAV proteins have been reported to induce cell extensions and microtubule bundling upon ectopic expression in transfected cells. However, these phenomena were mainly observed in neuronal cell types (van Haren et al., 2009). Similarly, we detected extensions in MCF10A epithelial cells and apparent microtubule bundling in fibroblast-like cells (COS7) ectopically expressing NAV3 (Fig. XXI).

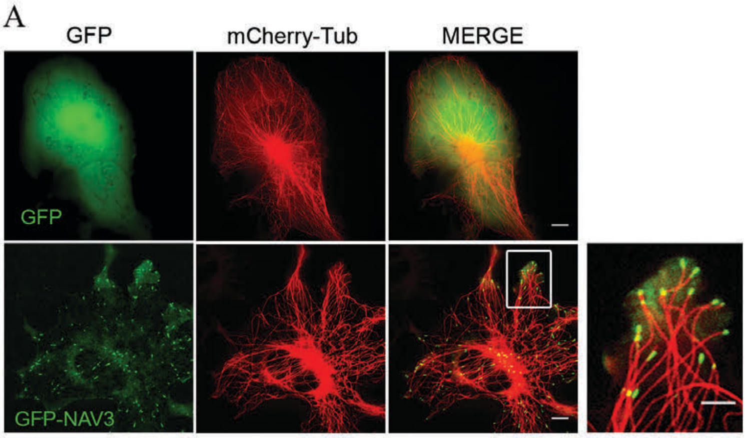

Figure XXI: ***NAV3 expression induces extension-like structures and alters cell morphology.***

Live images of COS-7 cells co-expressing either GFP (upper panel) or GFP-NAV3 (lower panel), along with mCherry-tubulin. The rectangle marks the enlarged area depicted in the far right panel. Bars, 10 and 5μm. (Note: this figure is identical to Fig. S4A of the original manuscript).

Does NAV3 interact with acetylated MT (for instance MTs that remain after nocodazole treatment)?

As shown in Figure XXII (below), NAV3 partly co-localized with acetylated tubulin prior to treatment with Nocodazole. However, following treatment we hardly detected co-localization. Presumably, by binding to microtubules, NAV3 prolongs their life span, thus allowing accumulation of acetylated tubulin. This association might be disrupted by Nocodazole.

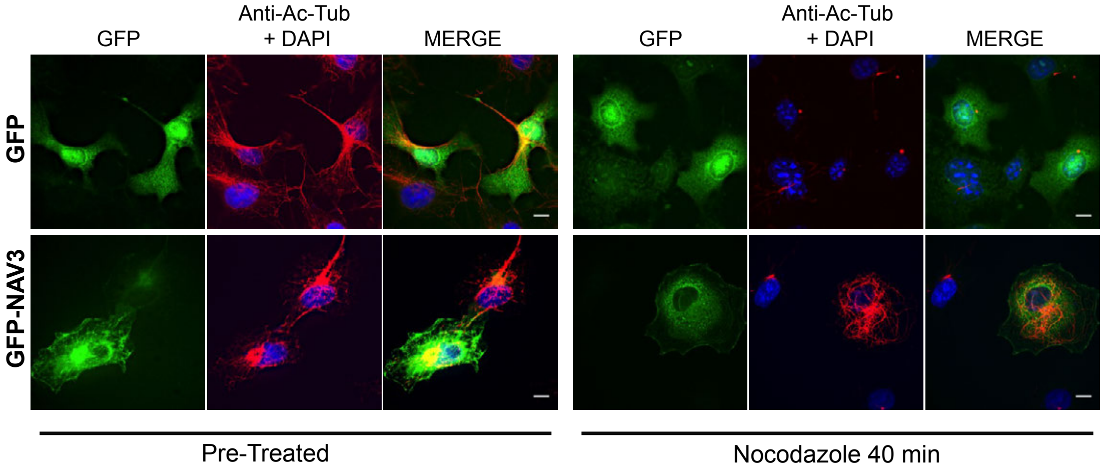

Figure XXII: ***Ectopic NAV3 localizes to microtubules and enhances abundance of acetylated tubulin.*** COS-7 cells ectopically expressing either GFP or GFP-NAV3 were treated for 40 minutes with Nocodazole (10 mM), prior to staining with an anti-acetyl tubulin antibody (red) and with DAPI (blue). White arrows indicate transfected cells and the scale bar is 10 mm. (Note: this figure is similar to Fig. S4E of the original manuscript).

7. For the TMA study, it is important to show the validation of the NAV3 antibody for immunohistochemistry.

Prior to the large-scale TMA study, we generated in mice a set mouse monoclonal antibodies against NAV3. Additionally, in collaboration with Sigma Israel we designed synthetic peptides and raised three different rabbit polyclonal antibodies, which are currently available to Sigma's customers. In preparation for the TMA analysis, we examined both the mono- and polyclonal antibodies for immunoprecipitation, immunoblotting, immunofluorescence and tissue staining. These studies selected monoclonal antibody 149 as the most suitable reagent for immunohistochemical surveys.

Two tests established specificity of our antibodies:

(i) Immunofluorescence of cells ectopically expressing GFP or a GFP fused to NAV3. As shown below, both monoclonal antibody 149 and the commercially available rabbit anti-NAV3 antibody yielded binding signals that were specific to NAV3-expressing cells (Fig. XXIII).

(ii) A western blot assay was performed on extracts prepared from NAV3-expressing cells, either prior or after immunoprecipitation using NAV3-specific antibodies. This experiment used control cells expressing GFP only (Fig. XXIV).

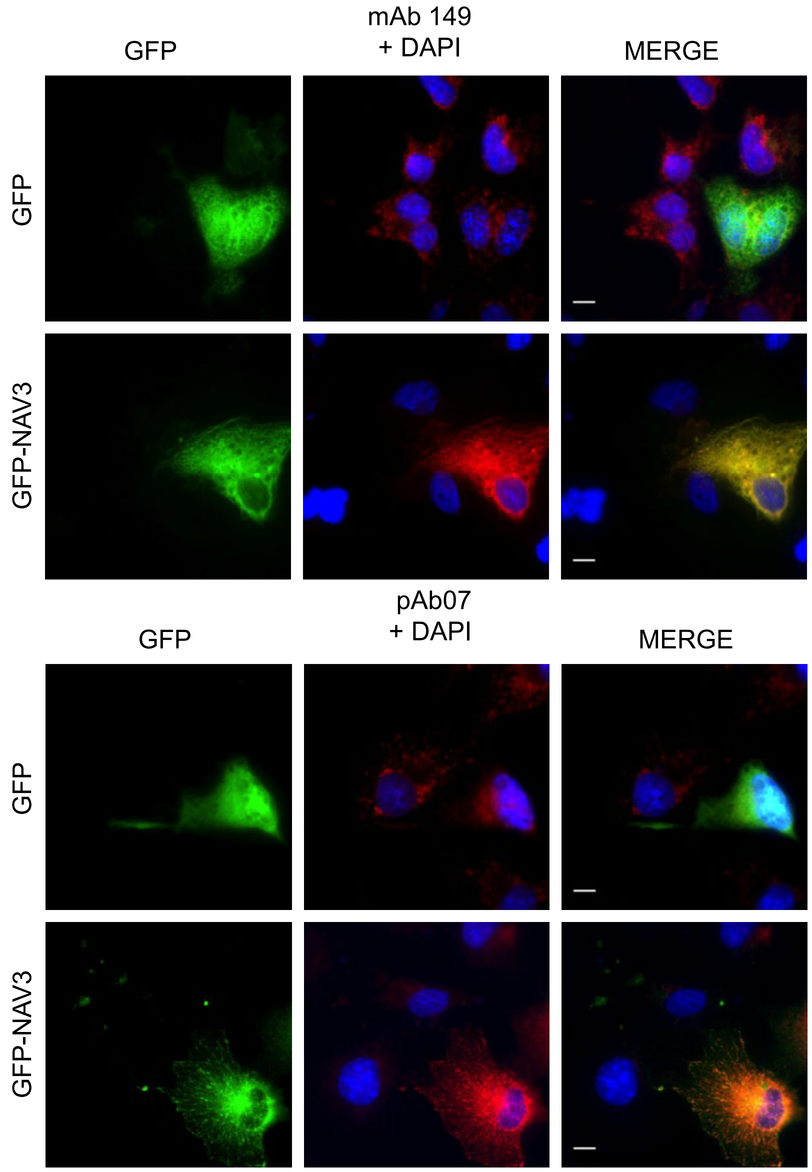

Figure XXIII: ***NAV3 is specifically recognized by monoclonal and polyclonal antibodies.*** COS-7 cells ectopically expressing either GFP or GFP-NAV3 were fixed and

double stained with anti-NAV3 (red) poly (pAb07) or monoclonal (mAb149) antibody and DAPI (blue). Bars, 10 mm.

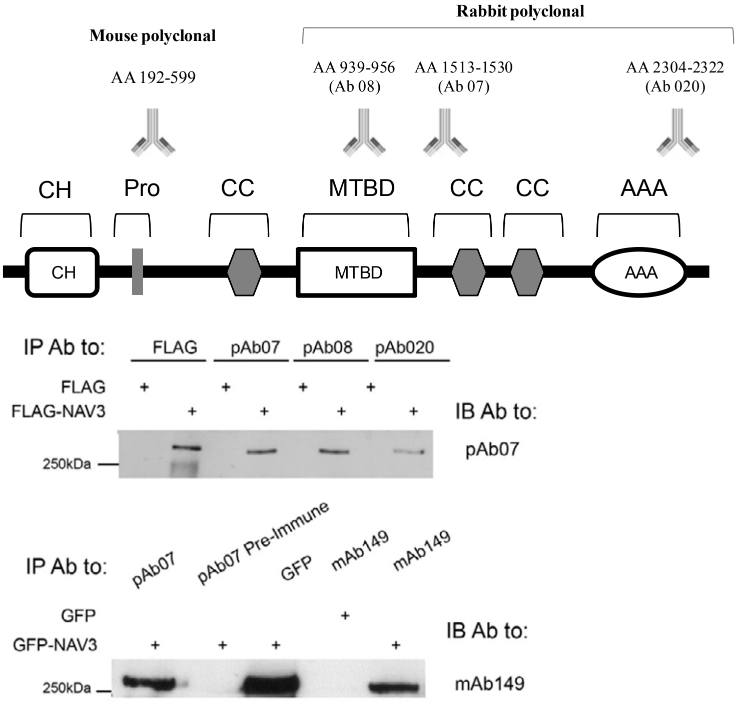

Figure XXIV: ***Specificity confirmation of antibodies to NAV3.***

(**Upper panel**) Schematic representation of the domain structure of NAV3 and the regions selected for generating murine mouse and rabbit polyclonal antibodies. The following domains are indicated; CH; Calponin homology, Pro; Proline rich, CC; Coiled-coil, MTBD; Microtubule binding domain, AAA; ATPase domain.

(**Middle panel**) Whole cell extracts of HEK293 cells previously transfected with either FLAG or FLAG-NAV3 were subjected to immunoprecipitation with the indicated polyclonal antibodies or incubated with beads conjugated to an anti-FLAG antibody. Protein detection was performed by using immunoblotting (IB) with an anti-NAV3 polyclonal antibody.

(**Lower panel**) Whole cell extracts of HEK293 cells previously transfected with plasmids encoding either GFP or GFP-NAV3 were subjected to immunoprecipitation with the indicated antibodies and to immunoblotting with an anti-NAV3 monoclonal antibody.

In Fig. 2B, the number of patients in each subgroup strong, moderate and absent/weak is not specified. In Fig 2C, the number of patients in the subpopulations is not mentioned.

As requested, we replaced Figure 7B with a new figure (see below; Fig. XXV), which specifies the number of patients per each group. Note that Figure 7C refers to the number of patients in each sub-population by calculating the corresponding fraction of patients (the total number of patients was 323).

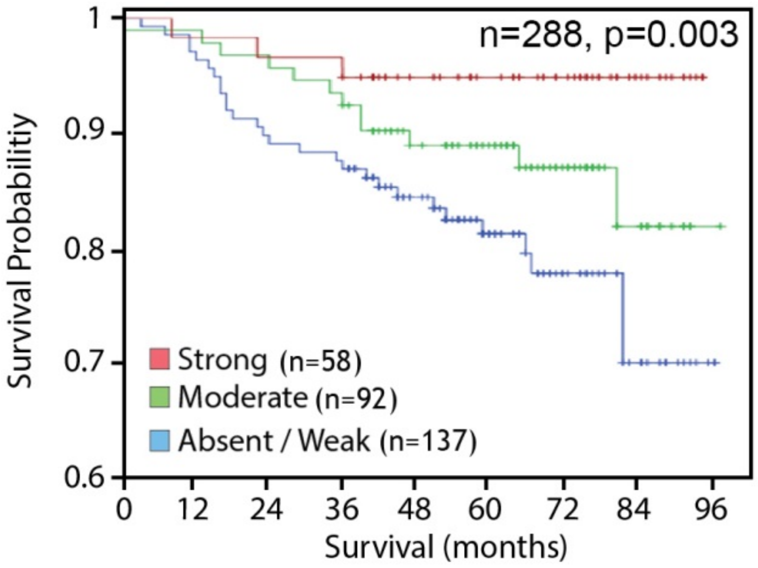

Figure XXV: ***Patient survival curves arranged according to NAV3 abundance, as determined using inmmunohistochemical analysis.***

Tissue microarrays were used to stratify 288 breast cancer patients for overall survival according to three groups categorized according to NAV3 abundance.

For the large study dataset, why was the study focused on ER+ patients?

We focused on the relatively large group of ER-positive patients because the association of NAV3 abundance with good prognosis reached statistical significance only within the ER-expressing group. This has been better explained in the revised manuscript.

According to the manuscript, 1471 patients were included in the study, but on Fig. 7D, n=200. A complete multivariate analysis should have been presented for both studies (as supplementary tables). The analyses suggest a correlation between loss of NAV3 and proliferation, as supported by some data.

In response to this comment, we revised the relevant text and legend to better explain that panels D and E of Figure 7 refer to two different clinical datasets of 200 (D) and 1471 (E) patients. As requested, we present below and in the revised manuscript a table that lists the available clinical data.

Disease parameters	NAV3 expression			p-Value
	Absent/Weak	Moderate	strong	
Molecular subtype				
n=323 (100%)				
Luminal A	84 (37%)	85 (37.4%)	58 (25.6%)	4.2 E-07
Luminal B	26 (72.2%)	8 (22.2%)	2 (5.6%)	
HER2+	19 (61.3%)	11 (35.5%)	1 (3.2%)	
Basal	23 (79.3%)	5 (17.2%)	1 (3.4%)	

Histological grade				
n=322 (99.7%)				
Low (I)	20 (28.6%)	23 (32.9%)	27 (38.6%)	2.1 E-07
Intermediate (II)	54 (40.6%)	53 (39.8%)	26 (19.5%)	
High (III)	78 (65.6%)	33 (27.7%)	8 (6.7%)	
Metastasis				
n=323 (100%)				
Without	135 (45.2%)	104 (34.8%)	60 (20%)	5.8 E-02
With	17 (70.8%)	5 (20.8%)	2 (8.4%)	
HER2				
n=323 (100%)				
no overexpression	107 (41.8%)	90 (35.2%)	59 (23%)	4.4 E-04
overexpression	45 (67.2%)	19 (28.4%)	3 (4.4%)	
Estrogen receptor				
n=323 (100%)				
negative	42 (70%)	16 (26.7%)	2 (3.3%)	1.8 E-05
positive	110 (41.8%)	93 (35.4%)	60 (22.8%)	
EGFR				
n=317				
Absent/Weak	132 (27.6%)	106 (35.7%)	59 (19.9%)	1.9 E-06
Moderate/strong	18 (90%)	1 (5%)	1 (5%)	

8. The description of experiments is not detailed enough and is sometimes confusing. To cite some examples: Which siRNA is used throughout the manuscript? In the methods section, 4 different siRNA are described, but only one is used in the study, which is not specified. What control is used in siRNA (or shRNA) experiments?

Following this comment, we extensively revised the Methods section of the manuscript. In reference to RNA interference, we already responded to some comments raised by this Referee in his/her first

specific question raised. In addition, it was clarified in the revised text that we made use of a mixture of 4 different siRNAs to NAV3, since the mixture exerted relatively effective knockdown. The four different siRNAs mentioned in the Methods section represent disassembly of the mixture (see Fig. XIII). The control siRNA was provided by the same vendor (Dharmacon), and it comprised scrambled ON-Target oligonucleotides, which are specific to no known RNA coding sequence. The same is relevant for the shRNAs we used: the control viral particles were produced from the plKO.1 plasmid, which encodes a hairpin loop specific to no known transcript sequence.

It is often unclear how cells have been challenged: starved, with FCS, with EGF? In the Transwell assays, it is not clear whether EGF has been added in the bottom chamber or in both chambers, and how cells were stained and migration quantified. It is mentioned at one point that control medium contains 5% serum? Does that mean that the EGF-treated cells are also in 5% serum?

As aforementioned, we reviewed and corrected all references to the incubation conditions. Notably, experiments in which EGF-stimulation was involved were preceded by culture starvation for 18 hours in order to synchronize all cells to the G_0-G_1 phase. This procedure is especially important for signalling studies, and it is routinely used in our lab. The full medium of MCF10A cells regularly contains 5% horse serum and other components (listed under Methods). Therefore, in the case of Transwell assays, either full medium supplemented with EGF, or EGF-free medium containing 5% serum were used, and wherever relevant EGF was added to the lower compartment. Cells in the upper compartment were grown in full medium without EGF. In case of RT-PCR for determining NAV3 mRNA levels, cells were first starved and this was followed by stimulation with either serum (5%) or EGF (10 ng/ml).

It is mentioned in the legend of Fig. S3 that the MDA-MB231 cells were starved? Why? Were the cells starved in all experiments? Was the Transwell assays performed for MDA-MB231 cells in medium with or without serum? This is not described. Why was the assay not performed in the presence of EGF, like the tracking assay (Fig. 5). Because the assays are not properly described, it is difficult for the reader to understand what events are EGF-dependent and what events are not.

MDA-MB231 cells, as opposed to MCF10A, do not require EGF in order to invade since they secrete their own growth factors through autocrine loops driven by mutant forms of RAS and p53. Therefore, most of the corresponding assays, including invasion assays, were performed in EGF-free serum-containing medium. Nevertheless, cells were stimulated with EGF in BrdU incorporation assays, chemotaxis assays and a few experiments in which cells were stimulated with EGF (e.g., Fig. 5). The legends of the relevant figures were corrected accordingly.

The way statistical analyses were performed is also confusing. Statistics must be presented for all experiments, including the supplementary data. If they are not presented, the authors must explain why. It seems that some experiments were not repeated (e.g. Fig. 1C-E, S3). This must be clearly stated.

The number of cells analysed per experiment must be specified for all experiments. And the number of times the experiments were repeated (e.g. Fig. 4B, only 8 or 9 cells analysed? From how many experiments?). For several experiments, it is not clear whether statistics were applied on cells within the same experiments or on independent biological replicates.

In response to these comments, we modified the legends of all relevant figures, in a way that clarifies the implemented statistical analysis. In general, qPCR analyses were repeated thrice or more (e.g., Fig 1C) and western blot analyses, for example Figure 1D, were usually repeated only twice. Figure 1E was replaced in order to satisfy another comment of this Referee. Among other changes, we modified the legend to Figure 4B, which refers now to "nine representative cells (3 of each experiment) were selected for analysis of MT's mean displacement and speed".

For example, for experimental metastasis, how many mice were included per data point? How many replicates? Were statistics applied on total mouse population or on independent biological replicates? The number of lung metastases is very different in the two tail-vein injection experiments: average of 2 in Fig. 3E and around 200 in Fig 6D? Is there an explication for the

large difference? The Y axis is labelled differently in each of the three metastasis experiments. Was the data quantified differently? In the metastasis from fat pad, do the indicated numbers (between 2000 and 4000) actually represent the number of metastases?

Each of the two different metastasis experiments was repeated twice, and each data point represents one animal. The summary statistics refers to all mice of the same experiment. To clarify the issue of statistics of metastasis assays, below we summarize all results (each data point corresponds to one animal). Note that the experimental metastasis data were quantified differently for the tail vain and for the fat pad settings. As indicated in the text relevant to Figure 4E, tail vain injections of MDA-MB-231 cells resulted in nodular metastases, and therefore the experiment was terminated after approximately 4 weeks. The observed range of nodules per lung was 0-30. By contrast, metastases originating in the fat pad involve a slower infiltration process of single cells or small clumps, hence this usually seeded hundreds to thousands micro-colonies. These differences are explained in the revised text.

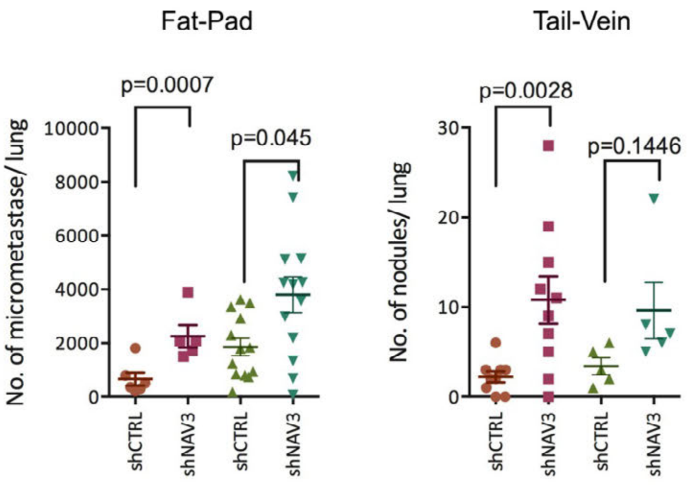

Figure XXVI: ***Integrated statistics of metastasis results obtained from four animal studies.***

The indicated RFP-expressing MDA-MB-231 derivatives were injected either into the mammary fat pad (left) or intravenously (tail vein; right) of SCID mice. Eight or four weeks later, respectively, lungs from injected mice were photographed, examined for RFP signals and the numbers of metastases or nodules per lung were quantified. Presented are two experiments of each kind, along with their respective *p*-values. Note that each dot represents an animal.

References

Akhmanova, A., Hoogenraad, C.C., Drabek, K., Stepanova, T., Dortland, B., Verkerk, T., Vermeulen, W., Burgering, B.M., De Zeeuw, C.I., Grosveld, F., *et al.* (2001). Clasps are CLIP-115 and -170 associating proteins involved in the regional regulation of microtubule dynamics in motile fibroblasts. Cell *104*, 923-935.

Akhmanova, A., and Steinmetz, M.O. (2008). Tracking the ends: a dynamic protein network controls the fate of microtubule tips. Nat Rev Mol Cell Biol *9*, 309-322.

Bleeker, F.E., Lamba, S., Rodolfo, M., Scarpa, A., Leenstra, S., Vandertop, W.P., and Bardelli, A. (2009). Mutational profiling of cancer candidate genes in glioblastoma, melanoma and pancreatic carcinoma reveals a snapshot of their genomic landscapes. Human mutation *30*, E451-459.

Debnath, J., and Brugge, J.S. (2005). Modelling glandular epithelial cancers in three-dimensional cultures. Nature reviews Cancer *5*, 675-688.

Deribe, Y.L., Wild, P., Chandrashaker, A., Curak, J., Schmidt, M.H., Kalaidzidis, Y., Milutinovic, N., Kratchmarova, I., Buerkle, L., Fetchko, M.J., *et al.* (2009). Regulation of epidermal growth factor receptor trafficking by lysine deacetylase HDAC6. Science signaling *2*, ra84.

Gao, Y.S., Hubbert, C.C., and Yao, T.P. (2010). The microtubule-associated histone deacetylase 6 (HDAC6) regulates epidermal growth factor receptor (EGFR) endocytic trafficking and degradation. The Journal of biological chemistry *285*, 11219-11226.

Guo, G., Gui, Y., Gao, S., Tang, A., Hu, X., Huang, Y., Jia, W., Li, Z., He, M., Sun, L.*, et al.* (2012). Frequent mutations of genes encoding ubiquitin-mediated proteolysis pathway components in clear cell renal cell carcinoma. Nat Genet *44*, 17-19.

Harris, T.H., Banigan, E.J., Christian, D.A., Konradt, C., Tait Wojno, E.D., Norose, K., Wilson, E.H., John, B., Weninger, W., Luster, A.D.*, et al.* (2012). Generalized Levy walks and the role of chemokines in migration of effector CD8+ T cells. Nature *486*, 545-548.

Honnappa, S., Gouveia, S.M., Weisbrich, A., Damberger, F.F., Bhavesh, N.S., Jawhari, H., Grigoriev, I., van Rijssel, F.J., Buey, R.M., Lawera, A.*, et al.* (2009). An EB1-binding motif acts as a microtubule tip localization signal. Cell *138*, 366-376.

Karenko, L., Hahtola, S., Paivinen, S., Karhu, R., Syrja, S., Kahkonen, M., Nedoszytko, B., Kytola, S., Zhou, Y., Blazevic, V.*, et al.* (2005). Primary cutaneous T-cell lymphomas show a deletion or translocation affecting NAV3, the human UNC-53 homologue. Cancer research *65*, 8101-8110.

Kumar, P., Lyle, K.S., Gierke, S., Matov, A., Danuser, G., and Wittmann, T. (2009). GSK3beta phosphorylation modulates CLASP-microtubule association and lamella microtubule attachment. The Journal of cell biology *184*, 895-908.

Lehmann, K., Janda, E., Pierreux, C.E., Rytomaa, M., Schulze, A., McMahon, M., Hill, C.S., Beug, H., and Downward, J. (2000). Raf induces TGFbeta production while blocking its apoptotic but not invasive responses: a mechanism leading to increased malignancy in epithelial cells. Genes & development *14*, 2610-2622.

Li, H., Duan, Z.W., Xie, P., Liu, Y.R., Wang, W.C., Dou, S.X., and Wang, P.Y. (2012). Effects of paclitaxel on EGFR endocytic trafficking revealed using quantum dot tracking in single cells. PloS one *7*, e45465.

Marzinke, M.A., Mavencamp, T., Duratinsky, J., and Clagett-Dame, M. (2013). 14-3-3epsilon and NAV2 interact to regulate neurite outgrowth and axon elongation. Archives of biochemistry and biophysics *540*, 94-100.

Nakaya, Y., Sukowati, E.W., Wu, Y., and Sheng, G. (2008). RhoA and microtubule dynamics control cell-basement membrane interaction in EMT during gastrulation. Nature cell biology *10*, 765-775.

Oksvold, M.P., Huitfeldt, H.S., and Langdon, W.Y. (2004). Identification of 14-3-3zeta as an EGF receptor interacting protein. FEBS Lett *569*, 207-210.

Pankov, R., Endo, Y., Even-Ram, S., Araki, M., Clark, K., Cukierman, E., Matsumoto, K., and Yamada, K.M. (2005). A Rac switch regulates random versus directionally persistent cell migration. The Journal of cell biology *170*, 793-802.

Petrie, R.J., Doyle, A.D., and Yamada, K.M. (2009). Random versus directionally persistent cell migration. Nat Rev Mol Cell Biol *10*, 538-549.

Sauer, L., Gitenay, D., Vo, C., and Baron, V.T. (2010). Mutant p53 initiates a feedback loop that involves Egr-1/EGF receptor/ERK in prostate cancer cells. Oncogene *29*, 2628-2637.

Taira, N., Yamaguchi, T., Kimura, J., Lu, Z.G., Fukuda, S., Higashiyama, S., Ono, M., and Yoshida, K. (2014). Induction of amphiregulin by p53 promotes apoptosis via control of microRNA biogenesis in response to DNA damage. Proceedings of the National Academy of Sciences of the United States of America *111*, 717-722.

van Haren, J., Draegestein, K., Keijzer, N., Abrahams, J.P., Grosveld, F., Peeters, P.J., Moechars, D., and Galjart, N. (2009). Mammalian Navigators are microtubule plus-end tracking proteins that can reorganize the cytoskeleton to induce neurite-like extensions. Cell Motil Cytoskeleton *66*, 824-838.

Wen, Y., Eng, C.H., Schmoranzer, J., Cabrera-Poch, N., Morris, E.J., Chen, M., Wallar, B.J., Alberts, A.S., and Gundersen, G.G. (2004). EB1 and APC bind to mDia to stabilize microtubules downstream of Rho and promote cell migration. Nature cell biology *6*, 820-830.

Wood, L.D., Parsons, D.W., Jones, S., Lin, J., Sjoblom, T., Leary, R.J., Shen, D., Boca, S.M., Barber, T., Ptak, J.*, et al.* (2007). The genomic landscapes of human breast and colorectal cancers. Science *318*, 1108-1113.

Yamazaki, D., Itoh, T., Miki, H., and Takenawa, T. (2013). srGAP1 regulates lamellipodial dynamics and cell migratory behavior by modulating Rac1 activity. Mol Biol Cell *24*, 3393-3405.

2nd Editorial Decision 12 September 2014

Thank you for the submission of your revised manuscript to EMBO Molecular Medicine. We have now heard back from the three Reviewers, whom we asked to re-evaluate your manuscript.

You will see that while Reviewers 1 and 2 are now globally positive, Reviewer 3 still has strong reservations.

Specifically, among other things, the main concerns remaining are 1) that the sensitivity and also specificity of the NAV3 antibody remain questionable and 2) Reviewer 3 still deems that the conclusion that NAV3 is a plus-end tracking protein is not fully supported by the data provided and 3) that the level of expression in the GOF experiments with GFP-NAV3 remains unclear.

Indeed, after our cross-commenting procedure, one of the more positive Reviewers admitted to the fact that, because so many experiments (and conclusions) rely on the antibody, perhaps more convincing controls are necessary. In fact, s/he suggested a number of experiments to do just that: 1) clarify, perhaps with microtubule staining if indeed Nav3 is found on ends; 2) show which bands are knocked down on a full blot with si and shRNA; 3) for IHC, confirm in cell pellets processed for IHC that the signal is decreased in knockdown cells vs. EGF-treated cells.

I agree that these issues are of great importance and thus we cannot offer publication at this stage.

It is EMBO Molecular Medicine policy to allow a single round of revision only but, after discussion with my colleagues, I have decided to allow you to submit a re-revised version that must include a rebuttal including the required experimental verification. Acceptance or rejection of the manuscript will depend on the completeness of your responses included in the next, final version of the manuscript.

I look forward to seeing a revised form of your manuscript as soon as possible.

***** Reviewer's comments *****

Referee #1 (Comments on Novelty/Model System):

The manuscript is in general convincing and the data are clearly presented. The involvement of Nav3 in breast cancer is novel and could be medically significant. The story is at times a bit confusing, likely because of the complexity of the systems being studied and the inability to actually relate in vitro and in vivo results. However, I think that there is enough new information here to make this a significant step forward in our understanding of the role of Nav3 in breast cancer. The mathematical modelling aspect doesn't really tell us much, but it is not wrong, just fairly trivial.

Referee #1 (Remarks):

This manuscript has been improved by revision and is now clearer and more convincing. The authors present a case that loss of Nav3 may inhibit apoptosis and enhance random migration of cancer cells. This may lead to increased recurrence or spread of tumours and thus worse outcome for patients. There are places where the story still seems quite unclear, but this first initial step

establishes that Nav3 may be an interesting protein to study in tumour progression.

Referee #2 (Comments on Novelty/Model System):

The manuscript has been considerably improved. The authors consider publishing some of the newer data as a follow-up paper.
I am favourable to this proposal.

Referee #2 (Remarks):

The revised manuscript and the rebuttal provide much more detailed mechanistic insights for Nav-3 function as a microtubule plus end stabilizer. They have clarified the counter-intuitive notion that directional migration promoted by high Nav-3 expression prevents invasive migration and metastasis.

Referee #3 (Remarks):

The authors have provided an extended and detailed reply to the comments of the reviewers. The authors have also provided some interesting preliminary data, even though these data do not actually address the points raised by the reviewers. Globally the manuscript itself has not changed much. Many of the responses do not appear in the manuscript (even when the authors say so) and thus will be of no help to the reader. More importantly, there was no significant improvement towards the major criticisms of the reviewers, that is, beyond the interesting observations made by the authors, the lack of mechanistic insights. We still don't know whether the various observations (e.g. the impact of NAV3 on microtubules, the anti-invasive phenotype...) are connected or not.
Many of the previous critiques are still valid and will not be reiterated. More importantly, the responses brought by the reviewers bring some new and important concerns:
- The sensitivity and specificity of antibody to NAV3 is questionable. Endogenous expression in various cell lines (at least as presented in Figure XIV, by WB) is barely detected unless EGF is added. The author did not mention which of the many bands stained with the NAV3 antibody correspond to NAV3 (which bands are extinguished with siRNAs). In immunofluorescence assays, the antibody does not stain endogenous NAV3 in COS7 cells (Fig. XXXIII, bottom panel) or does stain some aggregates (upper panel), which do not look like microtubules. Characterization of the antibody for immunohistochemistry, which is critical for the analysis of NAV3 expression levels in tumors, does not appear adequate, as validation of the antibody was only performed on cells overexpressing NAV3, by immunofluorescence and WB.
- NAV3 does not look like a plus-end tracking protein, except in very specific conditions (when overexpressed in COS7 cells). Endogenous NAV3 appears as aggregates in non-transfected COS7 cells (Figure XXIII), as a diffuse cytosolic staining (previous Figure 4C) or associated with the microtubule lattice in MDA-MB231 cells (Figure XX). This issue might be in part related to the quality of the antibody. A careful characterization of NAV3 localization in the various cell types used in the study is required. A systematic analysis of the impact of NAV3 overexpression/knockdown on cell morphology, formation of protrusions/lamellipodia, microtubules of the various cell types used in the study, would help understand what the actual impact of the protein on cell biology is.
- In gain of function experiments, the level of overexpression is still not shown except for the MDA-MB231 cells used for the experimental metastasis study. In that case it looks like GFP-NAV3 is expressed at much lower levels than endogenous NAV3 (at the protein level; Figure XVI). The authors do not explain why such low levels of GFP-NAV3 can have such a strong impact on metastasis formation.

Because of the points above, and the previously raised concerns, I do not recommend publication in EMBO Mol Med.

2nd Revision - authors' response 12 December 2014

List of Corrections and New Experiments Performed in Response to Requests Made by the Editor

Our responses, new data and respective corrections incorporated into the manuscript

1) Clarify, perhaps with microtubule staining if indeed Nav3 is found on ends

As requested, we co-stained COS-7 cells with antibodies to NAV3 and with antibodies to tubulin. The results shown in Figure I (below) clearly indicate that NAV3 localizes to the tips of microtubules. In addition, we obtained time-lapse images of live COS-7 cells co-expressing green NAV3 and red EB1, a protein that marks the growing tip of microtubules (see Figure II). The results confirm that NAV3 localizes just behind the EB1-decorated plus tips of dynamic microtubules. As a reminder, we show in Figure III a Supplementary panel from the original manuscript. The photos depict images from live COS-7 cells co-expressing GFP-NAV3 and mCherry-tubulin. Localization of NAV3 to the ends of microtubules is clearly demonstrated in this figure.

Altogether these findings indicate that NAV3 is localized to the ends of growing microtubules. This conclusion is consistent with the reported association of GFP-labelled mouse NAV1 with microtubule plus ends present in growth cones (Martinez-Lopez et al., 2005). Likewise, it has been reported that NAV2 is found in association with the microtubule cytoskeleton of SH-SY5Y cells (Muley et al., 2008). In addition, congruent with our observations, it has been shown that all three mammalian Navigators are +TIPs (van Haren et al., 2009). Note that Figures I and II were incorporated as Supplementary Figures in the revised manuscript.

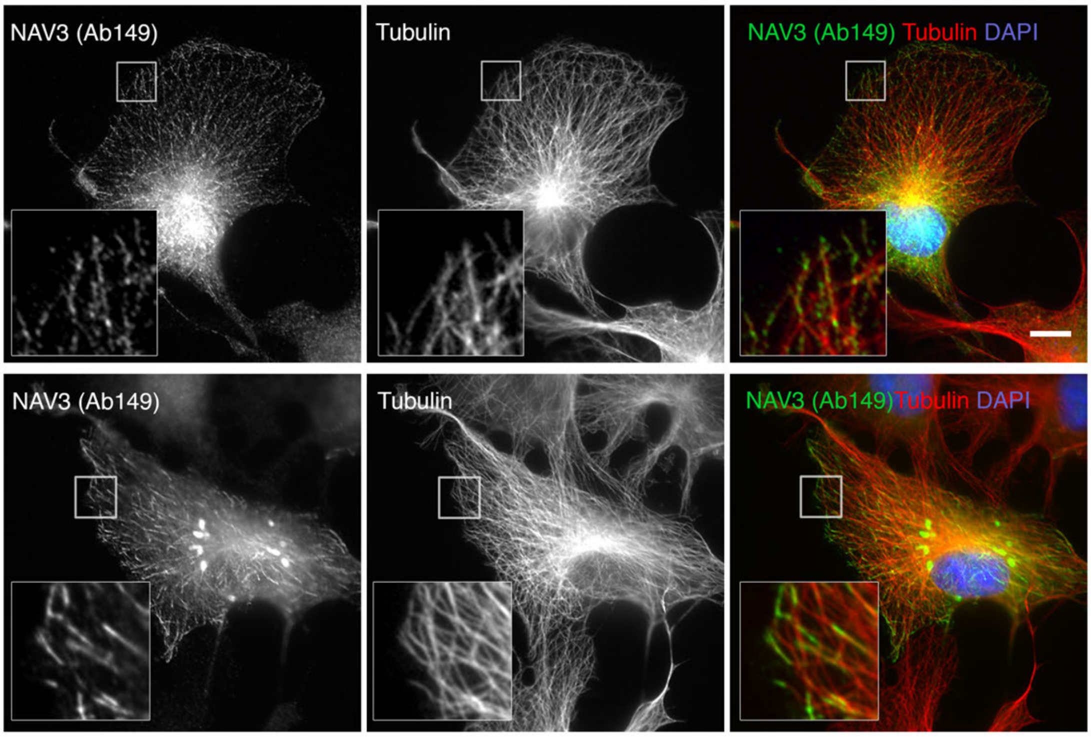

Figure I: NAV3 localizes to the growing ends of microtubules. Shown are images of COS-7 cells transiently expressing a FLAG-NAV3 protein. The cells were probed

using an anti-NAV3 antibody (monoclonal clone 149; generated in our laboratory) and an anti-tubulin antibody. Thereafter, cells were counterstained with DAPI, to visualize nuclei. Rectangles mark the enlarged areas, which are shown in the bottom left corners. Images of two cells are shown. Bar, 5 µm.

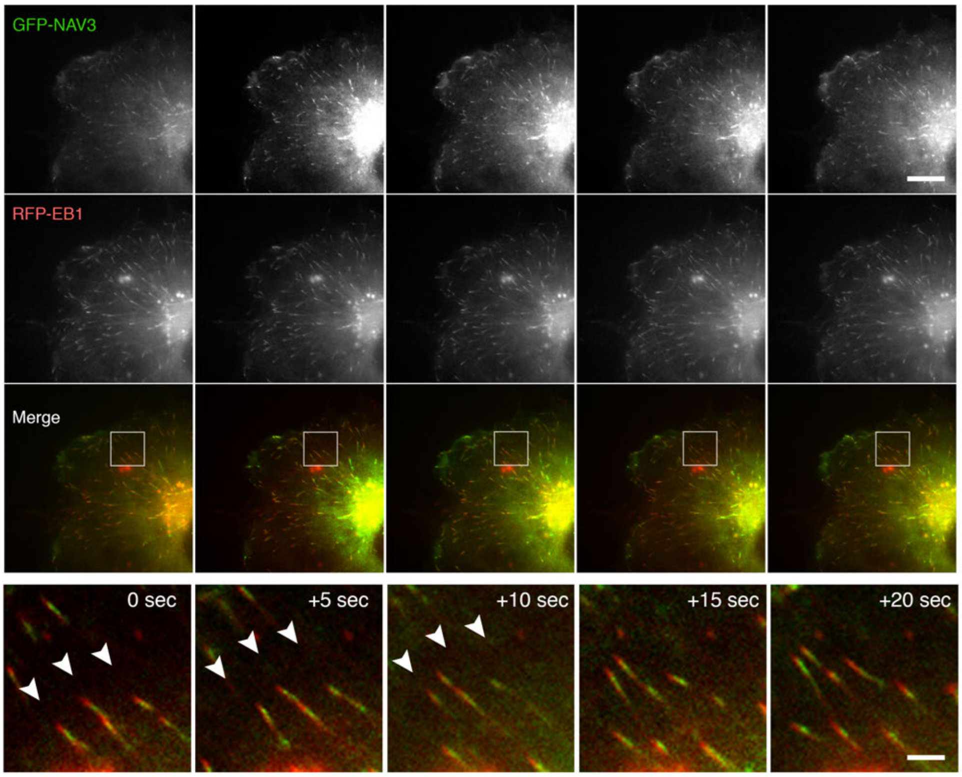

Figure II: NAV3 co-localizes with EB1 to the ends of microtubules. Shown are successive time-lapse images (5 seconds apart) of live COS-7 cells co-expressing GFP-NAV3 (green) and RFP-EB1 (red). The rectangles mark the enlarged areas depicted in the bottom row. The arrowheads mark individual microtubules. Note that the red channel was recorded first (for one second), followed by a short interval of 1.5 seconds and a similar recording from the green channel. For this reason, red-yellow-green triads represent co-localization. Bar, 10 µm.

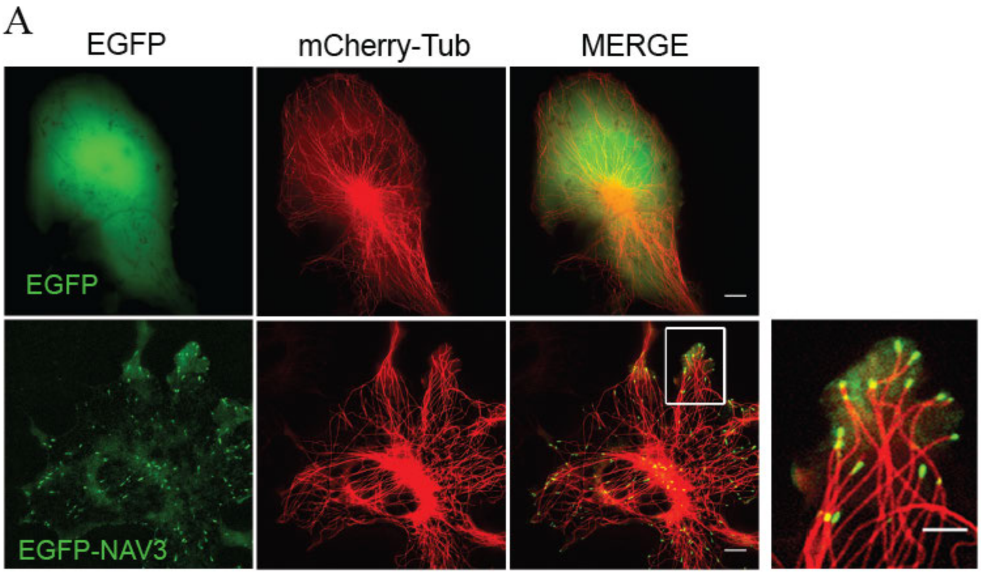

Figure III: NAV3 localizes to the ends of microtubules. Shown are images of live COS-7 cells expressing GFP-NAV3 (or GFP only; upper panels) along with mCherry-tubulin (lower panels). The rectangle marks the enlarged area depicted in the far right panel. Bars, 5 and 10 µm.

2) Show which bands are knocked down on a full blot with si and shRNA.

As requested, we present in Figure IV full blots of two different cell lines that were pre-treated with siRNA oligonucleotides specific to human NAV3. Whole extracts were prepared and probed for the NAV3 protein. Note that the Figure shows a loading control (Ras-GAP). It is evident that in both COS-7 and MDA-MB-231 cells the specific siRNAs depleted the high molecular weight band corresponding to NAV3. A lower, unidentified protein band was not affected, and this was consistent with the appearance of the loading control, namely Ras-GAP.

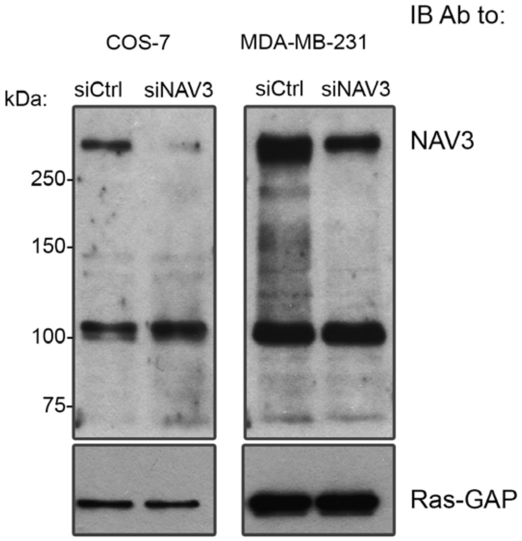

Figure IV: siRNA oligonucleotides specific to NAV3 diminish protein levels. COS-7 and MDA-MB-231 cells were transfected with the indicated siRNAs. Following 48 hours of incubation, whole cell lysates were prepared and subjected to immunoblotting analysis using anti-NAV3 (upper panel) and anti-Ras-GAP (lower panel; 120 kDa) antibodies.

The experiment presented in Figure V extends the full blot analysis to cells pre-treated with shRNAs. Whole extracts prepared from MDA-MB-231 cells stably expressing NAV3-specific shRNAs (or control shRNAs) were analysed before or after treatment with nocodazole. Similar to results obtained using siRNAs, we observed specific diminution of the NAV3 protein band. Note that other protein bands, including alpha-tubulin, were not affected.

A reciprocal approach complementing the use of siRNA and shRNA to specifically deplete a single protein band, is presented in Figure VI. Taking advantage of the ability of EGF to induce expression of NAV3 in mammary epithelial cells, the full blot we present depicts time-dependent up-regulation of the protein band corresponding to NAV3. Notably, other protein bands, including the loading control (alpha tubulin), displayed no similar response. Note, however, that a band migrating slightly below NAV3 shares inducibility with full length NAV3. This protein band might correspond to a degradation product of NAV3.

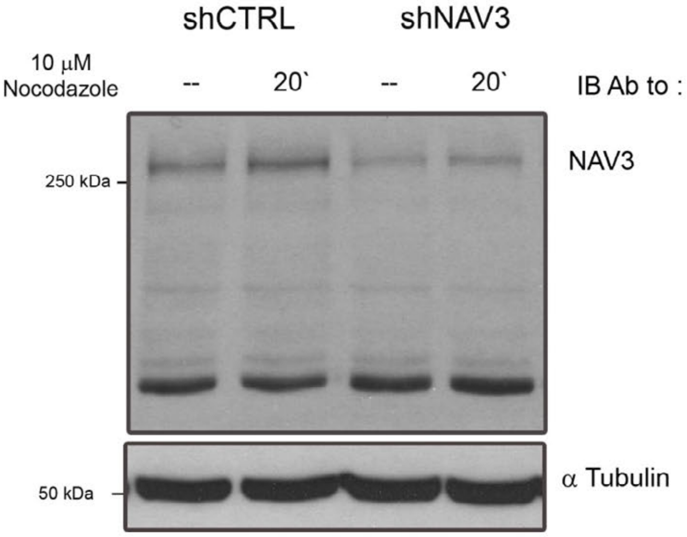

Figure V: NAV3 expression in MDA-MB-231 depleted cells treated with nocodazole. Stable derivatives of MDA-MB-231 cells ectopically expressing shRNAs corresponding to NAV3 (or control shRNAs) were serum starved, followed by treatment with nocadazole (10 mM) for 20 minutes. Thereafter, whole cell extracts were prepared and subjected to immunoblotting with anti-NAV3 and anti-alpha tubulin antibodies.

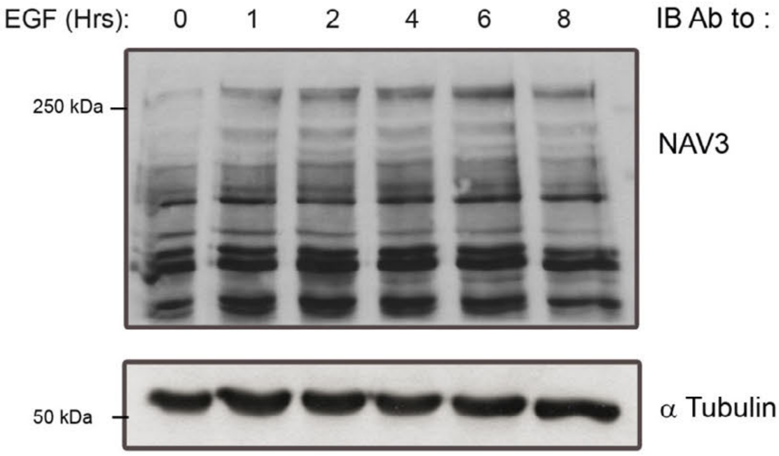

Figure VI: ***EGF stimulation enhances NAV3 levels in epithelial cells.*** MCF10A cells were serum-starved for 18 hours, followed by stimulation with EGF for the indicated time intervals. Thereafter, whole cell extracts were prepared and subjected to immunoblotting (IB) with anti-NAV3 and with anti-alpha tubulin antibodies.

3) For IHC, confirm in cell pellets processed for IHC that the signal is decreased in knockdown cells vs. EGF-treated cells

To enable the large-scale IHC analysis of breast cancer specimens, which we presented in Figure 7 of the original manuscript, we immunized mice with a recombinant fragment of human NAV3 (amino acids 192-599) and selected one monoclonal antibody, mAb 149. Additionally, in collaboration with Sigma Israel, a company that generates most antibodies for Sigma-Aldrich Inc., we designed three synthetic peptides and raised three different rabbit polyclonal antibodies (pAb 07, 08 and 020; see a scheme in Figure VII). Part of the quality control of the three antibodies was performed in our laboratory using mammalian cells ectopically expressing NAV3. These antibodies are currently available to Sigma-Aldrich's customers.

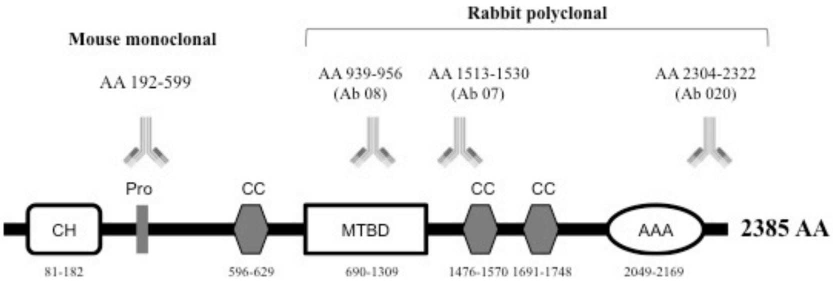

Figure VII: Schematic representation of the domain structure of NAV3 and the regions selected for generating murine and rabbit polyclonal antibodies. The following domains are indicated; CH; Calponin homology, Pro; Proline rich, CC; Coiled-coil, MTBD; Microtubule binding domain, AAA; ATPase domain.

The specificities of our monoclonal and polyclonal antibodies to NAV3 were established using the assays listed below:

(i) **Western blot assays** were performed on whole cell extracts prepared from HEK-293 cells expressing either a FLAG peptide tagged NAV3 or a GFP-NAV3 fusion protein. As control, we used FLAG only or GFP only expressing cells. Immunoprecipitation was performed using the three polyclonal antibodies or mAb 149. The results we obtained are presented in Figure VIII, along with the respective control IPs. As shown, all four antibodies were able to recognize NAV3.

(ii) **Immunofluorescence assays** of cells ectopically expressing GFP or a GFP fused to NAV3. As shown in Figure IX below, both anti-NAV3 antibodies we tested, mAb 149 and pAb 07, specifically recognized NAV3-expressing cells and showed no staining of cells expressing GFP alone.

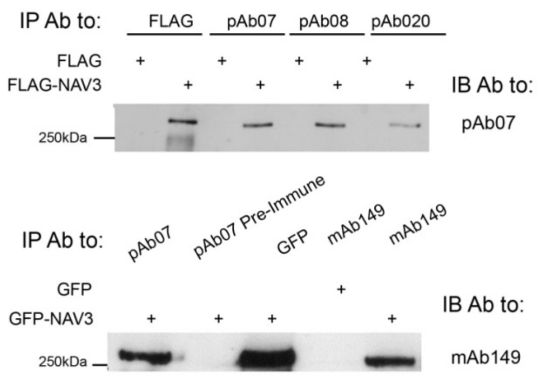

Figure VIII: Western blot analyses of the specificity of anti-NAV3 antibodies. Upper panel (polyclonal antibodies): Whole extracts of HEK-293 cells previously transfected with FLAG-NAV3 (or FLAG alone) were subjected to immunoprecipitation (IP) using the indicated polyclonal antibodies. Protein detection, following electrophoresis, was performed by using immunoblotting (IB) with pAb 07, a ploycIonal anti-NAV3 antibody. **Lower panel (a monoclonal antibody)** Whole extracts of HEK-293 cells previously transfected with plasmids encoding either GFP or GFP-NAV3 were subjected to immunoprecipitation (IP) with the indicated antibodies and this was followed by immunoblotting (IB) with an anti-NAV3 monoclonal antibody (mAb 149).

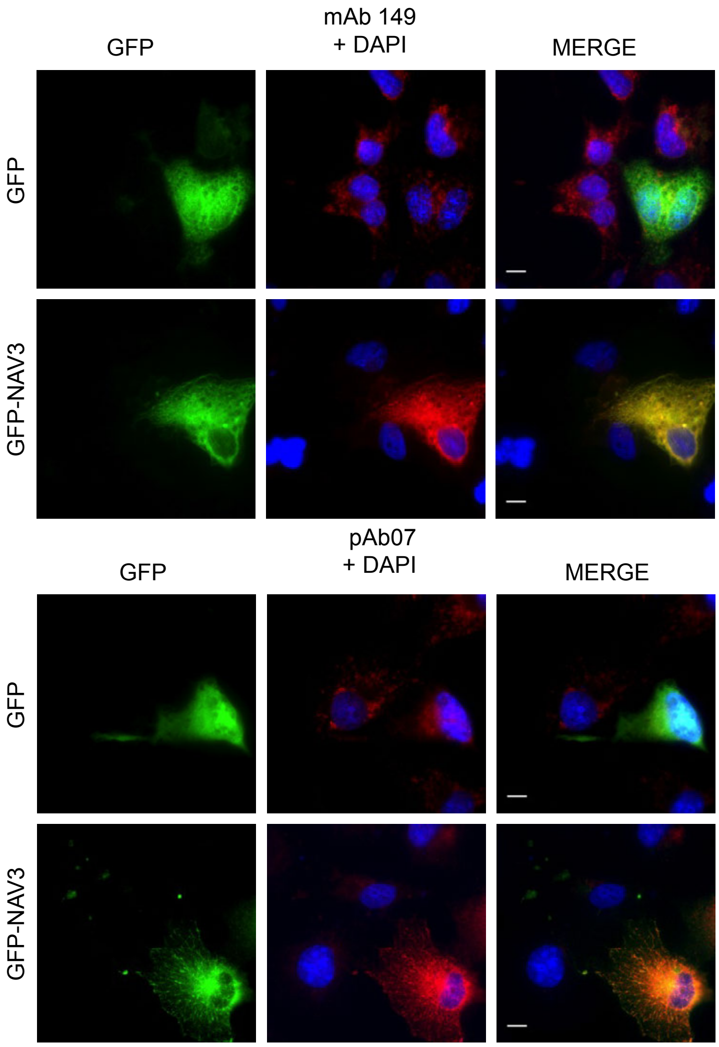

Figure IX: NAV3 is specifically recognized by monoclonal and polyclonal antibodies. COS-7 cells ectopically expressing either GFP or GFP-NAV3 were fixed and double stained with anti-NAV3 (red) polyclonal (pAb07) or monoclonal (mAb149) antibodies. DAPI was used for visualization of nuclei (blue). Bars, 10 mm.

As requested, we extended these control experiments to IHC. Both gain-of-function (cells ectopically overexpressing NAV3) and loss-of-function (cells pre-treated with NAV3-specific siRNA oligonucleotides) approaches were used. HEK-293 cells transiently overexpressing NAV3 were stained with mAb 149 and later fixed and embedded in paraffin, prior to processing for IHC analysis. The results presented in Figure X-A confirm stronger signals when testing the overexpressing cells. Likewise, comparison of MDA-MB-231 breast cancer cells, pre-treated with siRNAs against NAV3 (or with control siRNAs), confirmed weaker IHC signals after depletion of NAV3 expression (Fig. X-B).

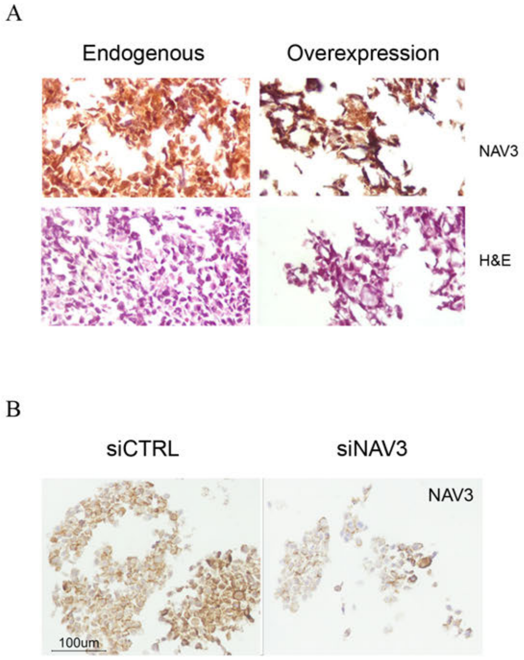

Figure X: Immunohistochemical analyses of cells overexpressing, or under-expressing NAV3. (**A**) HEK-293 cells were transfected with either GFP or GFP-NAV3-expression vectors. Two days later, the cells were fixed in formalin and embedded in paraffin blocks. Immunohistochemical analysis was performed with a monoclonal antibody to NAV3 (mAb149; upper panels). Hematoxylin and eosin staining was performed for histological examination. (**B**) MDA-MB-231 cells were transfected with siRNA oligonucleotides specific for NAV3 or with control siRNAs. Cells were processed for IHC analysis as in A.

To further confirm suitability of our monoclonal antibody, mAb 149, for IHC analysis, we applied it on paraffin blocks of representative human tissues (Figure XI). Tissues were selected on the basis of data listed in the Human Protein Atlas dataset (http://www.proteinatlas.org). In accordance with the dataset, we observed highest signals when analysing pancreatic tissues and very weak or no signals when analysing lymph nodes. Other tissues displayed intermediate IHC staining intensities.

© EMBO

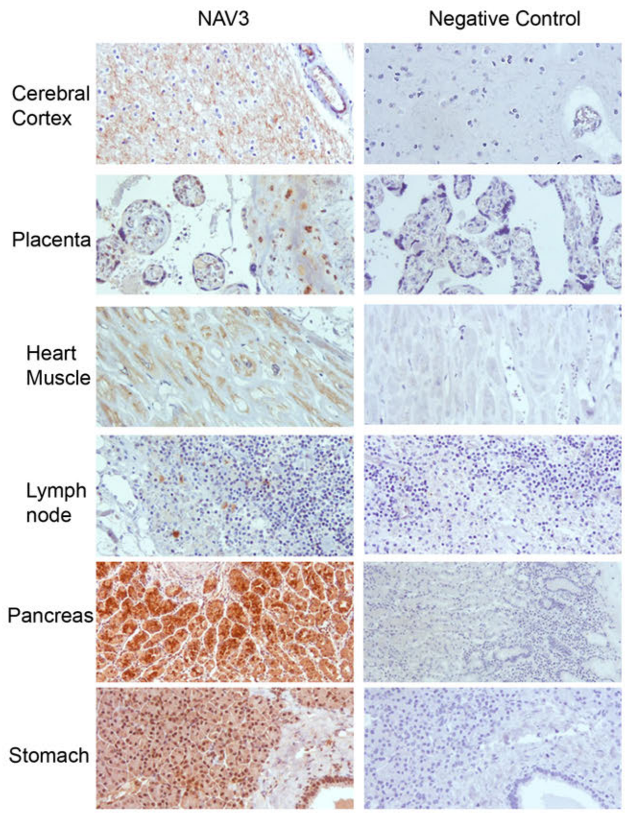

Figure XI: Immunohistochemistry analyses of human tissues for NAV3 expression. Representative human tissues from cerebral cortex, placenta, heart muscle, lymph node, pancreas and stomach were stained with either a monoclonal antibody to NAV3 (mAb 149) or with an isotype matched murine immunoglobulin (negative control). Original magnification, 200X.

In summary, our immunohistochemical assays made use of both human tissues and cultured cells genetically manipulated to overexpress, or under-express, NAV3. Altogether, the results we obtained lend strong evidence in favour of suitability of the antibodies we used and the tissue processing procedures we applied in our main study to multiple cancer specimens.

References

Martinez-Lopez, M.J., Alcantara, S., Mascaro, C., Perez-Branguli, F., Ruiz-Lozano, P., Maes, T., Soriano, E., and Buesa, C. (2005). Mouse neuron navigator 1, a novel microtubule-associated protein involved in neuronal migration. Mol Cell Neurosci *28*, 599-612.

Muley, P.D., McNeill, E.M., Marzinke, M.A., Knobel, K.M., Barr, M.M., and Clagett-Dame, M. (2008). The atRA-responsive gene neuron navigator 2 functions in neurite outgrowth and axonal elongation. Dev Neurobiol *68*, 1441-1453.

van Haren, J., Draegestein, K., Keijzer, N., Abrahams, J.P., Grosveld, F., Peeters, P.J.,

Moechars, D., and Galjart, N. (2009). Mammalian Navigators are microtubule plus-end tracking proteins that can reorganize the cytoskeleton to induce neurite-like extensions. Cell Motil Cytoskeleton *66*, 824-838.

3rd Editorial Decision 18 December 2014

Thank you for the submission of your revised manuscript to EMBO Molecular Medicine. We have now received the enclosed report from the Reviewer that was asked to re-assess it. As you will see, sh/e are now globally supportive and I am pleased to inform you that we will be able to accept your ismanuscript pending the following final amendments:

1) As per our Author Guidelines, the description of all reported data that includes statistical testing must state the name of the statistical test used to generate error bars and P values, the number (n) of independent experiments underlying each data point (not replicate measures of one sample), and the actual P value for each test (not merely 'significant' or 'P < 0.05'). We appreciate that this has been complied with in the majority of cases, but there appear to be some remaining ones.

2) Every published paper now includes a 'Synopsis' to further enhance discoverability. Synopses are displayed on the journal webpage and are freely accessible to all readers. They include a short standfirst (to be written by the editor) as well as 2-5 one sentence bullet points that summarise the paper (to be written by the author). Please provide the short list of bullet points that summarise the key NEW findings. The bullet points should be designed to be complementary to the abstract - i.e. not repeat the same text. We encourage inclusion of key acronyms and quantitative information. Please use the passive voice. Please attach these in a separate file or send them by email, we will incorporate them accordingly.

3) The manuscript must include a statement in the Materials and Methods identifying the institutional and/or licensing committee approving the experiments, including any relevant details (like how many animals were used, of which gender, at what age, which strains, if genetically modified, on which background, housing details, etc). We encourage authors to follow the ARRIVE guidelines for reporting studies involving animals. Please see the EQUATOR website for details: http://www.equator-network.org/reporting-guidelines/improving-bioscience-research-reporting-the-arrive-guidelines-for-reporting-animal-research/. Please make sure that all the above details are reported.

4) For experiments involving human subjects the authors must identify the committee approving the experiments and include a statement that informed consent was obtained from all subjects and that the experiments conformed to the principles set out in the WMA Declaration of Helsinki [http://www.wma.net/en/30publications/10policies/b3/] and the NIH Belmont Report [http://ohsr.od.nih.gov/guidelines/belmont.html]. Any restrictions on the availability or on the use of human data or samples should be clearly specified in the manuscript. Any restrictions that may detract from the overall impact of a study or undermine its reproducibility will be taken into account in the editorial decision. While not all these provisions may apply in this case, we do encourage you to move your statements concerning human samples to the Materials and Methods section of the manuscript.

5) We are now encouraging the publication of source data, particularly for electrophoretic gels and blots, with the aim of making primary data more accessible and transparent to the reader. Would you be willing to provide a PDF file per figure that contains the original, uncropped and unprocessed scans of all or at least the key gels used in the manuscript? The PDF files should be labeled with the appropriate figure/panel number, and should have molecular weight markers; further annotation may be useful but is not essential. The PDF files will be published online with the article as supplementary "Source Data" files. If you have any questions regarding this just contact me.

Please submit your revised manuscript within the first few days of 2015 so that we may proceed with formal acceptance and dispatch to production.

***** Reviewer's comments *****

Referee #1 (Comments on Novelty/Model System):

No new remarks

Referee #1 (Remarks):

The authors have satisfactorily answered all of the queries and provided all of the relevant controls requested.
